# Impaired Repopulating Ability of *Uhrf2*^−/−^ Hematopoietic Progenitor Cells in Mice

**DOI:** 10.3390/genes14081531

**Published:** 2023-07-27

**Authors:** Takahiro Sano, Koki Ueda, Keiji Minakawa, Tsutomu Mori, Yuko Hashimoto, Haruhiko Koseki, Yasuchika Takeishi, Kazuhiko Ikeda, Takayuki Ikezoe

**Affiliations:** 1 Department of Hematology, Fukushima Medical University School of Medicine, Fukushima 960-1295, Japan; 2 Department of Blood Transfusion and Transplantation Immunology, Fukushima Medical University School of Medicine, Fukushima 960-1295, Japan; 3 Department of Human Life Sciences; Fukushima Medical University School of Nursing, Fukushima 960-1295, Japan; 4 Department of Diagnostic Pathology, Fukushima Medical University School of Medicine, Fukushima 960-1295, Japan; 5 Laboratory for Developmental Genetics, RIKEN Center for Integrative Medical Sciences, Wako 351-0198, Japan; 6 Department of Cardiovascular Medicine, Fukushima Medical University School of Medicine, Fukushima 960-1295, Japan

**Keywords:** *UHRF2*, hematopoietic stem and progenitor cells, hematopoiesis

## Abstract

UHRF proteins catalyze the ubiquitination of target proteins and are involved in regulating gene expression. Some studies reported a reduced expression of UHRF2 in acute leukemia cells, but the role of UHRF2 in hematopoiesis remains unknown. Here, we generated *Uhrf2^−/−^* mice to clarify the role of *UHRF2* deletion in hematopoiesis. Compared to *Uhrf2^+/+^* mice, *Uhrf2^−/−^* mice showed no differences in complete blood counts, as well as bone marrow (BM) findings and spleen weights. Proportions of cells in progenitor fractions in BM were comparable between *Uhrf2^+/+^* mice and *Uhrf2^−/−^* mice. However, in competitive repopulation assays with BM transplants (BMT), the proportions of *Uhrf2^−/−^* cells were decreased relative to *Uhrf2^+/+^* cells in all lineages. After the second BMT, *Uhrf2^−/−^* neutrophils were few, while 20–30% of *Uhrf2*^−/−^ T cells and B cells were still detected. RNA sequencing showed downregulation of some genes associated with stem-cell function in *Uhrf2^−/−^* hematopoietic stem/progenitor cells (HSPCs). Interestingly, trimethylated histone H3 lysine 9 was increased in *Uhrf2^−/−^* HSPCs in a cleavage under targets and tagmentation assay. While *UHRF2* deletion did not cause hematologic malignancy or confer a growth advantage of HSPCs, our results suggest that *UHRF2* may play a role in the regulation of hematopoiesis.

## 1. Introduction

The E3 ubiquitin ligases, UHRF (ubiquitin-like with PHD and RING finger domains) proteins, including UHRF1 and UHRF2 proteins, contain functional domains consisting of ubiquitin-like plant homeodomain (PHD), really interesting new gene (RING), and methyl-DNA-binding SET and RING-associated (SRA) domains [1,2]. The SRA and PHD domains recognize various DNA or proteins, while the RING domain catalyzes the ubiquitination of target proteins. E3 ligase activity of the RING domain in UHRF1 mono-ubiquitylates histone H3 at K14, K18, and K23, and the RING domain of UHRF2 recognizes substrates for ubiquitylation of various proteins including PCNP and cyclin D1 [2]. The multiple domains of UHRF proteins provide a hub for the regulatory cell-cycle networks, DNA damage repair, and epigenetic modification. Thus, UHRF proteins play important roles in the pathogenesis of cancers.

UHRF1 and UHRF2 proteins share a highly similar structural homology with each other, but the expressions of these proteins are different in cancers. *UHRF1* is highly expressed in the many types of cancers and is implicated in tumorigenesis as an oncogene [2,3,4]. In contrast, *UHRF2* is downregulated in various cancers, suggesting that UHRF2 may play a role as a tumor suppressor [1,5,6,7,8]. Regarding hematologic cancers, UHRF2 is barely expressed in some leukemic cell lines, including K562, Jurkat, and RAJI cells, while it is significantly expressed in RS4:11, SEMK, and NALM6 cells [5]. However, primary cells from most acute myeloid leukemia patients showed low *UHRF2* mRNA levels [5,9]. On the other hand, it has been reported that *Uhrf2* deletion reduced progression of colon cancer with low *Apc* expression in mice [10].

Despite accumulating knowledge regarding various biological functions and low expression in leukemic cells of UHRF2, the role of UHRF2 in hematopoiesis remains unclear. Here, we investigated possible roles of UHRF2 in hematopoiesis using the *Uhrf2^−/−^* mice which we generated. Although there was no clear hematologic phenotype in peripheral blood or bone marrow (BM) of *Uhrf2^−/−^* mice, *Uhrf2^−/−^* hematopoietic stem/progenitor cells (HSPCs) showed impaired ability of hematopoietic reconstitution in competitive repopulation assays with BM transplants (BMT).

## 2. Materials and Methods

### 2.1. Gene Targeting and Mice

**Figure 1:**
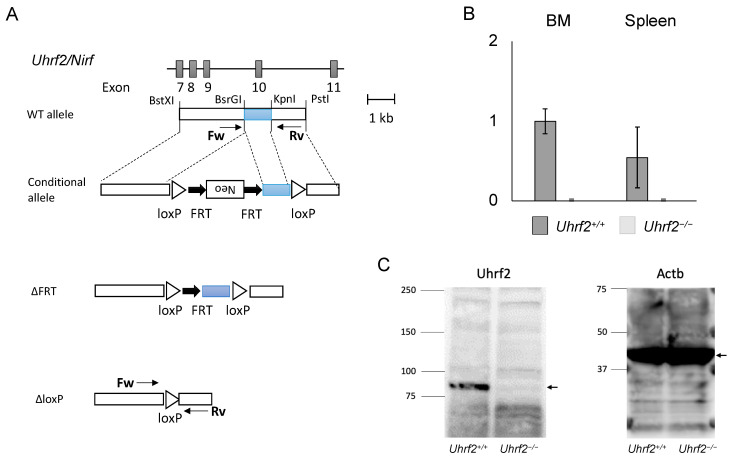
*Uhrf2* KO mice. (**A**) Schematic representations of *Uhrf2* genomic organization and its targeting strategy. (**B**) *Uhrf2* mRNA expressions in *Uhrf2*^+/+^ and *Uhrf2*^−/−^ mice by qRT-PCR. (**C**) Expression of Uhrf2 protein in spleen of *Uhrf2*^+/+^ and *Uhrf2*^−/−^ mice by Western blot. Actb protein was used as the control. Allows indicate predicted sizes (kDa) of Uhrf2 and Actb proteins.

All mice were on a C57BL6/J background. *Uhrf2* heterozygous knockout (KO) mice (*Uhrf2^−/+^* mice) were generated using R1 ESCs according to a standard protocol, as described previously [11]. In brief, as the conditional allele, FRT-Neo with loxP was inserted into BsrGI and KpnI sites at introns 9 and 10, respectively (*Uhrf2^fl/fl^*, *Neo^FRT/FRT^*). After Neo deletion with FLP (*Uhrf2^fl/fl^*), exon 10 of *Uhrf2*, which encodes part of the SRA domain that is essential for functions of Uhrf2 because it binds to various proteins and epigenetically modified DNA [1,2], was removed by the Cre–loxP system (*Uhrf2^∆/∆^*). Then, mice were backcrossed with C57BL6/J mice over 10 generations. Mice were identified by polymerase chain reaction (PCR) of tail genomic DNA. Sequences of PCR primers were in introns 9 (forward: ACCTGAGTGTACCATGGATAG) and 10 (reverse: GAGCGTGTGGTAAGACTGATG). The sizes of PCR products were 1075 bp and 252 bp for *Uhrf2* wildtype and KO alleles, respectively (Figure 1A). *Uhrf2^−/−^* homozygous KO mice and *Uhrf2^+/+^* wild type (WT) littermate mice born from *Uhrf2^−/+^* parents were used for experiments. For the competitive repopulation assay with BMT, C57BL/6-Ly-5.1 WT mice were purchased from the RIKEN-BRC (Tsukuba, Japan).

### 2.2. Cell Preparations and Counts

Mouse hematopoietic cells were prepared, as described previously [12,13]. Briefly, peripheral blood cells of mice were drawn from the tail vein and counted by XT-1800i (Sysmex, Kobe, Japan). Peripheral white blood cells (WBCs) were obtained by lysing samples with BD Pharm Lyse (BD, Franklin Lakes, NJ, USA). BM mononuclear cells were collected by grinding bones and separated with centrifugation through Ficoll (Histopaque-1083; Sigma-Aldrich, St. Louis, MO, USA).

### 2.3. Quantitative RT-PCR

The total RNA was used for reverse transcription (RT) with RevarTra Ace qPCR RT Master Mix (TOYOBO, Osaka, Japan). cDNA was subjected to quantitative RT-PCR (qRT-PCR) with THUNDERBIRD qPCR Mix (TOYOBO) and TaqMan gene expression assay for *Uhrf2* and *Actb* (assay ID: Mm00520043_m1 and Mm00607939_s1, Thermo Fisher Scientific, Waltham, MA, USA) performed using a Quantstudio3 (Thermo Fisher Scientific) with the comparative CT (ddCT) method.

### 2.4. Histopathology

Hematoxylin and eosin stain was performed for paraffin-embedded samples with a standard protocol. Pictures were taken and digitized by a BX53 microscope (Olympus, Tokyo, Japan).

### 2.5. Flow Cytometry and Cell Sorting

Flow cytometry and sorting of cells were performed using a FACSCanto II and FACSAria II (both from BD) [12]. The results of flow cytometry were analyzed using FLOWJO software (BD). Cells were stained with eFluora450-B220, PerCP/Cy5.5-TCRβ, and APC-Gr1 or perCP/Cy5.5-Sca-I, PE/Cy7-cKit, Alexa647-CD34, PE-CD16/32, and Biotin-Lineage (CD3e, CD4, CD5, CD8a, CD11, B220, Ter119, and Gr1) followed by visualization with APC/Cy7-streptavidin. For chimerism analyses, eFluora450-B220, PerCP/Cy5.5-TCRβ, APC-CD45.1, and PE-CD45.2 (all from eBioscience, San Diego, CA, USA) were used. HSPCs and hematopoietic committed progenitor cells were determined as described previously [14]. In brief, fractions of lineage^−^Sca1^+^c-Kit^+^ (LSK) cells enriched with HSPCs and lineage^−^Sca1^−^c-Kit^+^ cells containing committed progenitor cells were identified in the gate determined according to the forward scatter and side scatter. Common myeloid progenitor (CMP), granulocyte–macrophage progenitor, and megakaryocyte–erythroid progenitor cells, in the lineage^−^Sca1^−^c-Kit^+^-committed progenitor fraction, were identified using fluorescence of CD34 and CD16/CD32 (Figure S1).

### 2.6. Competitive Repopulation Assay with Bone Marrow Transplantation 

To study the repopulating ability of *Uhrf2*^−/−^ HSPCs, the standard competitive repopulation assay using BMT, without immune attack between donor and recipient cells, was performed as described previously [12,14]. In brief, the hematopoiesis was reconstituted in lethally irradiated C57BL/6-Ly-5.1 recipient mice with mixtures of bulk BM cells from two types of donors with the same allotype each other at the ratio of 1:1—*Uhrf2^−/−^* mice with WBCs that express CD45.2 and C57BL/6-Ly-5.1 mice (same as recipient mice) with CD45.1^+^ WBCs. Then, 2.5 × 10^6^ BM cells of each genotype were injected into recipient mice conditioned with 9.0 Gy 24 h before BMT. Proportions of CD45.2^+^ *Uhrf2*^−/−^ cells and CD45.1^+^ *WT* cells in fractions of Gr1^+^ granulocytes, B220^+^ B cells, and TCR^+^ T cells were evaluated to determine the chimerism by flow cytometry in the recipients. 

### 2.7. RNA Sequencing (RNAseq)

RNAseq for HSPCs was performed as described previously [12,15]. In brief, total RNA obtained from each sample of flow-sorted primary LSK cells, which represent HSPCs, from BM of three mice in each genotype was subjected to a sequencing library construction using the Ovation Single-Cell RNA-Seq System (TECAN, Männedorf, Switzerland) according to the manufacturer’s protocol. The quality of the libraries was assessed with an Agilent 2200 TapeStation High Sensitivity D1000 (Agilent Technologies, Santa Clara, CA, USA). The equally pooled libraries of the samples were sequenced using the HiSeq system (Illumina, San Diego, CA, USA) in 51 bp single-end reads. Sequencing adaptors, low-quality reads, and bases were trimmed with the Trimmomatic-0.32 tool [16]. The sequence reads were aligned to the mouse reference genome (mm10) using Tophat 2.0.13 (bowtie2-2.2.3) [17], which can adequately align reads onto the location including splice sites in the genome sequence. The aligned reads were subjected to downstream analyses using StrandNGS 3.0 software (Agilent Technologies). The read counts allocated for each gene and transcript (RefSeq version 2013.4.1) were quantified using the reads per kilobase of exon per million reads mapped (RPKM) method. 

Affected pathways or gene set enrichment were evaluated using the comparison analysis in IPA™ (Ingenuity Pathways Analysis, Qiagen, Hilden, Germany) or gene set enrichment analysis (GSEA, Broad Institute, Cambridge, MA, USA), according to the RPKM value for each gene [15].

### 2.8. Cleavage under Targets and Tagmentation Assay (CUT&Tag)

Cryopreserved mouse flow-sorted primary BM LSK cells from six mice in each genotype were sent to Active Motif (Carlsbad, CA, USA) for CUT&Tag. Briefly, cells were incubated overnight with Concanavalin A beads and 1 µL of the primary anti-H3K9me3 antibody per reaction (Active Motif, catalog number 39161). After incubation with the secondary anti-rabbit antibody (1:100), cells were washed, and tagmentation was performed at 37 °C using protein-A-Tn5. Tagmentation was halted by the addition of EDTA, SDS, and proteinase K at 55 °C, after which DNA extraction and ethanol purification were performed, followed by PCR amplification and barcoding (as described in the Active Motif CUT&Tag kit, catalog number 53160). Following SPRI bead cleanup (Beckman Coulter, Brea, CA, USA), the resulting DNA libraries were quantified and sequenced on Illumina’s NextSeq 550 (8 million reads, 38 paired end). Reads were aligned using the BWA algorithm (mem mode; default settings) [18]. Duplicate reads were removed, and only reads that mapped uniquely (mapping quality ≥ 1) and as matched pairs were used for further analysis. Alignments were extended in silico at their 3′-ends to a length of 200 bp and assigned to 32 nt bins along the genome. The resulting histograms (genomic “signal maps”) were stored in bigWig files. Peaks were identified using the MACS 2.1.0 algorithm at a cutoff of *p* = 1 × 10^−7^, without a control file, and with the -nomodel option. Peaks that were on the ENCODE blacklist of known false ChIP-Seq peaks were removed. Signal maps and peak locations were used as input data to Active Motif’s proprietary analysis program, which creates Excel tables containing detailed information on sample comparison, peak metrics, peak locations, and gene annotations. For differential analysis, reads were counted in all merged peak regions (using Subread), and the replicates for each condition were compared using DESeq2 [19].

### 2.9. Statistical Analysis

The statistical significance was determined using an unpaired two-tailed Student’s *t*-test. All *p*-values were two-sided, with *p*-values < 0.05 considered significant.

## 3. Results

### 3.1. Uhrf2 KO Mice and Hematologic Findings

We first obtained heterozygous *Uhrf2* KO (*Uhrf2^−/+^*) mice by crossing a founder *Uhrf2*^−/−^ mouse with a WT *Uhrf2^+/+^* mouse. We next crossed *Uhrf2^−/+^* mice with each other. From *Uhrf2^−/+^* parents, *Uhrf2^+/+^*, *Uhrf2^−/+^*, and *Uhrf2^−/−^* mice were born at expected Mendelian ratios. *Uhrf2* mRNA was not detectable in the BM and spleen of *Uhrf2^−/−^* mice (Figure 1B). Then, *Uhrf2^+/+^* mice and *Uhrf2^−/−^* mice after the fifth generation were used for further analyses. There were no differences in body weight and survival rate during observation between *Uhrf2^+/+^* mice and *Uhrf2^−/−^* mice until 1 year after birth.

**Figure 2:**
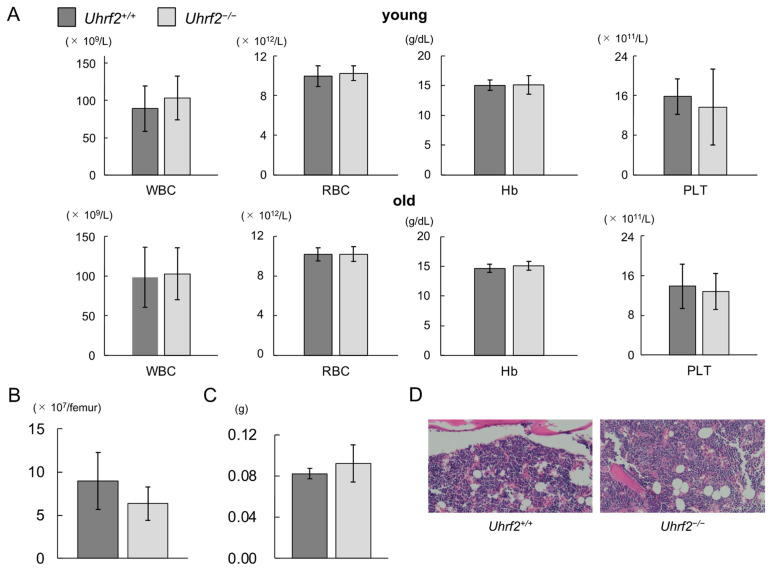
Hematopoiesis of *Uhrf2* KO mice. (**A**) Complete blood counts in young (3 months old, N = 11 and 14 in *Uhrf2*^+/+^ and *Uhrf2*^−/−^ mice, respectively) and old (12 months old, N = 17 in each genotype) mice. Peripheral blood counts of white blood cells (WBC), red blood cells (RBC), platelets (PLT), and hemoglobin levels (Hb) are shown. (**B**) The total bone marrow (BM) nuclear cell numbers from the ground right femur (N = 5 and 8 in *Uhrf2*^+/+^ and *Uhrf2*^−/−^ mice, respectively). (**C**) Spleen weights (N = 5 and 3 in *Uhrf2*^+/+^ and *Uhrf2*^−/−^ mice, respectively). (**D**) Sternum bone marrow histology with hematoxylin and eosin stain (×100). (**A**–**C**) Data are shown as means ± standard deviations (SDs).

Peripheral WBC counts, red blood cell counts, hemoglobin levels, and platelet counts were similar between *Uhrf2^+/+^* mice and *Uhrf2^−/−^* mice at young and old ages (Figure 2A). There was no significant difference between *Uhrf2^−/−^* mice and *Uhrf2^+/+^* mice in BM cellularity (Figure 2B,D) or spleen weight (Figure 2C) at 3 months old. Histopathological study also showed no specific findings in BM of *Uhrf2^−/−^* mice compared with *Uhrf2^+/+^* mice (Figure 2D). 

### 3.2. Impairment of HSPC Repopulating Capacity in Uhrf2^–/–^ Mice

**Figure 3:**
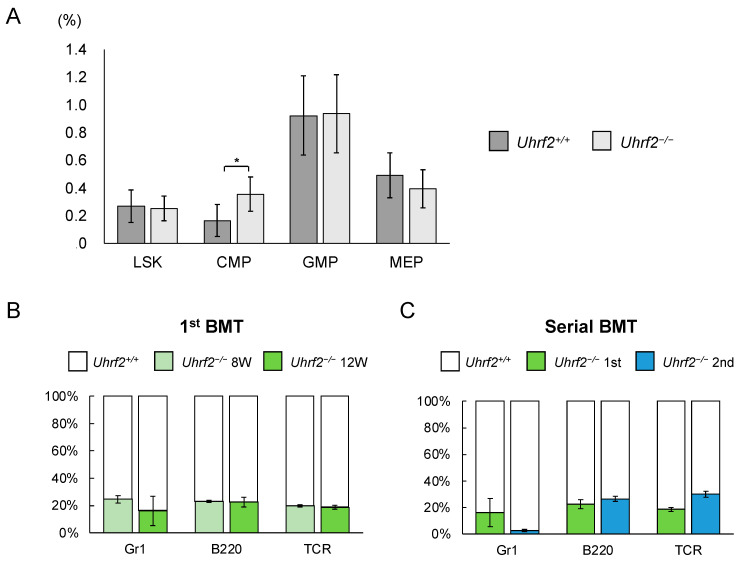
Hematopoietic stem and progenitor cells in *Uhrf2* KO mice. (**A**) The ratio of each fraction in BM by flow cytometry (percentage in all gated cells) (N = 5 and 10 in *Uhrf2*^+/+^ and *Uhrf2^−/−^* mice, respectively). (**B**,**C**) The competitive repopulation assay using bone marrow transplant (BMT) was performed. The mixture of cells consisting of CD45.2^+^ *Uhrf2^−/−^* and CD45.1^+^ wild-type BM cells, at the ratio of 1:1, was injected into each lethally irradiated wildtype recipient mice with CD45.1^+^ BM cells. The proportion of CD45.2^+^ *Uhrf2*^−/−^ cells and CD45.1^+^ *WT* cells in each fraction was evaluated as chimerism using flow cytometry in the recipients. The proportions of CD45.2^+^ *Uhrf2^−/−^* or CD45.1^+^ wildtype BM-derived Gr1^+^ myeloid cells, B220^+^ B cells and TCR^+^ T cells after the first ((**B**), N = 4) and serial ((**C**), N = 3) BMTs are shown. LSK, lineage^−^Sca1^+^cKit^+^ hematopoietic stem/progenitor cell; CMP, common myeloid progenitor cells; GMP, granulocyte–macrophage progenitor cells; MEP, megakaryocyte–erythroid progenitor cells. Data are shown as means ± SDs. * *p* < 0.05.

We evaluated ratios and functions of HSPC fractions of *Uhrf2*^−/−^ mice. There was a significant difference in the proportion of CMP cells between *Uhrf2*^−/−^ and *Uhrf2*^+/+^ mice, but not in the proportions of LSK cells, granulocyte–macrophage progenitor cells, or megakaryocyte–erythrocyte progenitor cells (Figure 3A). However, the absolute number of CMP cells in BM did not significantly differ between *Uhrf2*^−/−^ and *Uhrf2*^+/+^ mice (2.21 ± 1.11 vs. 1.28 ± 0.90 × 10^5^ cells per femur, *p* = 0.13). Next, we performed competitive repopulation assay with BMT. Chimerism of *Uhrf2*^−/−^ cells was reduced relative to *Uhrf2^+/+^* cells in all lineages after the first BMT (Figure 3B), suggesting the impairment of poor proliferative capacity of *Uhrf2*^−/−^ hematopoietic progenitors. Then, engraftment of *Uhrf2^−/−^* granulocytes almost failed, while B cells and T cells remained at minor ratios, after the second BMT (Figure 3C). This finding may indicate a limited self-renewal capacity of hematopoietic stem cells (HSCs) of *Uhrf2*^−/−^ mice. 

### 3.3. Gene Expression in HSPCs of Uhrf2^–/–^ Mice

**Figure 4:**
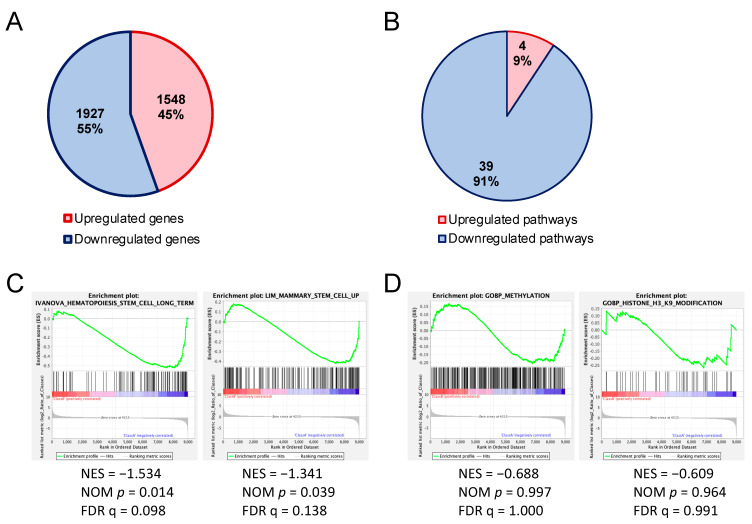
Altered gene expressions in hematopoietic stem and progenitor cells of *Uhrf2* KO mice. (**A**,**B**) Numbers and proportions of upregulated and downregulated genes (>2-fold) (**A**) and canonical pathways (**B**) in LSK cells from *Uhrf2*^−/−^ mice compared to those from *Uhrf2*^+/+^ mice in RNA sequencing. (**A**) Individual upregulated and downregulated genes are shown in the Supplementary Tables. (**B**) Pathway analysis was performed using the comparison analysis in IPA™ (Ingenuity Pathways Analysis, Qiagen, Hilden, Germany). Individual pathways are shown in the Supplementary Tables. (**C**,**D**) Gene set enrichment analysis (GSEA) for genes associated with stem-cell capacity (**C**) and gene ontology for methylation and H3K9 modification (**D**).

We sought the cause of impairment of the HSPC reconstitution ability of *Uhrf2^−/−^* mice by comparing the gene expression of HSPC-enriched LSK cells of *Uhrf2^−/−^* mice with those from *Uhrf2^+/+^* mice using RNAseq. Regarding the differentially expressed genes (>2-fold), there was a slightly greater number of downregulated genes than upregulated genes in LSK cells of *Uhrf2^−/−^* mice (Figure 4A). These downregulated genes included *Gfi1b* (6.2-fold), *Flt3* (2.2-fold), and *Stat5a* (2.3-fold), which are important for the maintenance and proliferation of HSPCs (Tables S1 and S2).

Pathway analysis with IPA^TM^ of RNAseq results exhibited a greater number of downregulated [z-score < −2.0, −log(*p*-value) > 1.30] canonical pathways than upregulated canonical pathways [z-score > 2.0, −log(*p*-value) > 1.30] in *Uhrf2*^−/−^ LSK cells compared to *Uhrf2*^+/+^ WT cells (Figure 4B). The 39 downregulated pathways included “CXCR4 signaling”, “HIF1α signaling”, “Ephrin receptor signaling”, “acute myeloid leukemia signaling”, and “UVB-induced MAPK signaling” pathways, whereas there were only four upregulated pathways, including the “oxidative phosphorylation” and “cyclins and cell-cycle regulation” pathways (Tables S3–S5). GSEA of RNAseq results showed significant enrichments (FDR q < 0.25) of downregulated genes that are associated with stem-cell capacity in *Uhrf2*^−/−^ LSK cells (Figure 4C).

### 3.4. Enrichment of H3K9me3 in HSPCs of Uhrf2^−/−^ Mice

**Figure 5:**
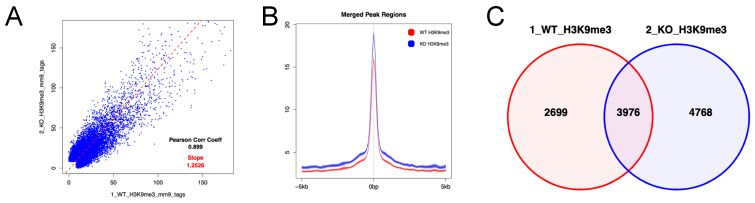
H3K9me3 in HSPCs of *Uhrf2* KO mice. CUT&Tag analysis was performed for LSK cells from *Uhrf2*^+/+^ mice (WT) and *Uhrf2*^−/−^ mice (KO). (**A**) Peak correlation scatter plot. For each pairwise comparison, a scatter plot was generated plotting the tag numbers of sample 1 against sample 2 for each merged region. The slope (dotted line) is a measure for the average ratio in tag numbers between the two samples. (**B**) Average plots. Tag distributions across all peak regions spanning 5 kb of gene bodies are presented. (**C**) Venn diagram for the overlap between peaks.

We next analyzed enrichment of histone H3 trimethylated at lysine 9 (H3K9me3) in the region encompassing 5 kb upstream and downstream of gene body regions in LSK cells using CUT&Tag, because it has been reported that the tandem Tudor domain (TTD) of *Uhrf2* interacts with H3K9me3 [2,20,21,22]. CUT&Tag showed that the increases in H3K9me3 was more frequent, but not universal, in *Uhrf2^−/−^* LSK cells compared to *Uhrf2^+/+^* LSK cells (Figure 5). The increase in H3K9me3 level was not limited to the promotor region (Figure S2). Methylation- or H3K9 modification-related genes were not significantly enriched (FDR q > 0.25) in RNAseq, although many involved genes that were differentially expressed, as shown in Figure 4D.

## 4. Discussion

Expressions of *UHRF2* are reduced in many types of human cancers, including acute myeloid leukemia, gastric cancer, pancreatic cancer, lung cancer, and head and neck cancer [1,2,5,9]. Loss of *UHRF2* is associated with progression of cancer [6,8] and *UHRF2* is a negative regulator of epithelial–mesenchymal transition [7], suggesting the role of *UHRF2* as a tumor suppressor. By contrast, Li et al. [10] recently reported that *UHRF2* was predominantly expressed in human samples of colorectal adenocarcinoma, and high *UHRF2* expression was associated with shortened survival. In addition, KO of *Uhrf2* extended survival time by suppressing the progression of colon cancer associated with minimum *Apc* expression in mice [10]. In other lines of *Uhrf2* KO mice, growth defects were not detected, while seizure at advanced age or a partial impairment in spatial memory acquisition was observed [23,24]. Our *Uhrf2*^−/−^ mice did not show any hematologic disease or solid tumor spontaneously, during the observation period of 1 year after birth, although neurological dysfunctions cannot be ruled out as we did not investigate them. 

The UHRF proteins show different expression patterns in normal cell differentiation. Usually, *UHRF2* is poorly expressed in undifferentiated cells and its expression level rises as these cells differentiate, whereas *UHRF1* is expressed more abundantly in undifferentiated cells than in differentiated cells [1,2]. However, *UHRF2* expression in undifferentiated cells has also been reported in retinal progenitor cells [25], the stem/progenitor compartment of intestinal tumors, and intestinal crypt cells [10]. In these progenitor fractions, *UHRF2* contributes to both proliferation and differentiation. In hematopoietic cells, expression of *UHRF2* is greater in lymphoid lineages than in myeloid lineages at both progenitor and mature levels [2]. In the present study, deletion of *Uhrf2* resulted in an increased proportion of BM CMP cells, but not all other stem/progenitor fractions in BM. Considering that *Uhrf1* deletion was reported to lead to erythroid-biased differentiation of HSCs [26], *Uhrf2* might contribute to commitment of HSCs. However, the number of mature blood cells in peripheral blood was comparable between *Uhrf2*^−/−^ mice and *Uhrf2^+/+^* mice. On the other hand, serial BMT experiments exhibited reduced repopulating ability of CD45.2^+^ *Uhrf2^−/−^* cells, especially in myeloid cells, relative to CD45.1^+^ cells with WT *Uhrf2* from C57BL/6-Ly-5.1 mice. Inversely, competitive BMT of CD45.2^+^ cells from WT C57BL6/J mice vs. CD45.1^+^ C57BL/6-Ly-5.1 mice in our laboratory, under the same condition as the present study, showed a rather higher proportion of C57BL6/J-derived CD45.2^+^ cells in the fraction of granulocytes (around 40%) compared to that of T cells or B cells (around 15% or 20%, respectively), after the second BMT [12]. There is still a limitation concerning our use of WT C57BL6/J mice instead of the *Uhrf2^+/+^* WT littermates of *Uhrf2^−/−^* mice in these transplants.

Functional impairment of HSPCs associated with *Uhrf2* deletion may be compatible with the roles of *Uhrf2* in proliferation of other progenitors rather than with tumor suppressive roles. In fact, there were several genes that play important roles in HSC functions among downregulated genes in *Uhrf2*^−/−^ LSK cells. For example, *Gfi1b* can control the functional integrity of HSCs via the Wnt/β-catenin signaling pathway [27] or act as a metabolic regulator controlling stemness and differentiation potential of HSCs [28]. Furthermore, genes involving long-term HSCs were globally downregulated in GSEA of *Uhrf2*^−/−^ LSK cells. Furthermore, pathway analysis with IPA™ showed downregulation of “acute myeloid leukemia signaling”, as well as important pathways known for HSC maintenance and differentiation in *Uhrf2*^−/−^ HSPCs, including “CXCR4 signaling” [29], “HIF-1α signaling” [30], and “Ephrin receptor signaling” [31]. In addition, UHRF2 has been reported to be essential for maintaining cell viability after UV irradiation [32], and our pathway analysis also showed downregulation of the “UVB-induced MAPK signaling” pathway. On the other hand, the “oxidative phosphorylation pathway” was also upregulated in *Uhrf2*^−/−^ LSK cells. Considering that alteration of metabolic profiles takes place during hematopoietic differentiation [33,34], upregulation of this pathway may partly explain the mechanism of increase in the proportion of CMP cells differentiated from HSCs due to a rewiring toward differentiation rather than self-renewal of HSCs. Upregulated pathways in *Uhrf2*^−/−^ LSK cells also included “cyclins and cell-cycle regulation”. Interestingly, it was previously reported that E3 ubiquitylation activity of UHRF2 involves cyclins D1 and E1, leading to G1 arrest [1]. 

UHRF2 has been implicated in epigenetic modifications, including DNA methylation and recognition of H3K9me3 [2], which contribute to regulations of gene expressions. CUT&Tag showed a general enrichment of H3K9me3 in HSPCs of *Uhrf2^−/−^* mice compared to those of *Uhrf2^+/+^* mice. Because the TTD domain of UHRF2 protein, with activity of a ubiquitin ligase, recognizes H3K9me3 [2,20,21,22], the alteration of H3K9me3 status in *Uhrf2*^−/−^ LSK cells may be associated with deletion of UHRF2 protein containing the TTD domain. However, we could not find a factor leading to HSPC proliferation that is regulated by H3K9me3. Moreover, there were a few genes that were differentially expressed and altered in H3K9 methylation in *Uhrf2*^−/−^ LSK cells, and we did not examine the relationship between *Uhrf2* deletion and DNA methylation profile. These are the main limitations of the present study, and in-depth studies will unveil how Uhrf2 affects DNA methylation and regulates HSPC proliferation. Another limitation of the present study was that we did not study the HSC populations in more detail, using SLAM markers such as CD150 and CD48 [35], although we found global downregulation of genes associated with long-term HSCs.

## 5. Conclusions

The present study showed that *Uhrf2* may play a role in HSPC ability to reconstitute hematopoiesis. Despite some alteration of gene expressions, deletion of UHRF2 did not cause hematologic malignancy or confer a growth advantage of HSPCs. However, our results suggest that *UHRF2* may play a role in the regulation of hematopoiesis.

## Data Availability

The RNAseq and CUT&Tag data were deposited in the Gene Expression Omnibus database (GSE230415, GSE230833).

## Supplementary Materials

The following supporting information can be downloaded at: https://www.mdpi.com/article/10.3390/genes14081531/s1: Figure S1: Gating of committed hematopoietic progenitor cells; Figure S2: Pie charts of genomic features in *Uhrf2*^−/−^ LSK cells in CUT&Tag; Table S1. Upregulated genes in *Uhrf2*^−/−^ LSK cells in RNA sequencing; Table S2. Downregulated genes in *Uhrf2*^−/−^ LSK cells in RNA sequencing; Table S3. Upregulated canonical pathways in *Uhrf2^−/−^* LSK cells in RNA sequencing determined by IPA™ (Ingenuity Pathways Analysis, Qiagen); Table S4. Downregulated canonical pathways in *Uhrf2*^−/−^ LSK cells in RNA sequencing determined by IPA™; Table S5. Enriched canonical pathways with undetermined regulation (|z score| ≤ 2.0 or uncalculatable) in *Uhrf2*^−/−^ LSK cells in RNA sequencing determined by IPA™.
