# Supplementary material for: Impaired Repopulating Ability of Uhrf2−/− Hematopoietic Progenitor Cells in Mice
Source: Genes (Basel). 2023 Jul 27;14(8):1531. doi: 10.3390/genes14081531 (PMC10454722; doi:10.3390/genes14081531)
Supplement: Supplementary file 1 [file genes-14-01531-s001.zip › Supplemental materials/Figure S1.pdf]

Figure S1. Gating of committed hematopoietic progenitor cells.

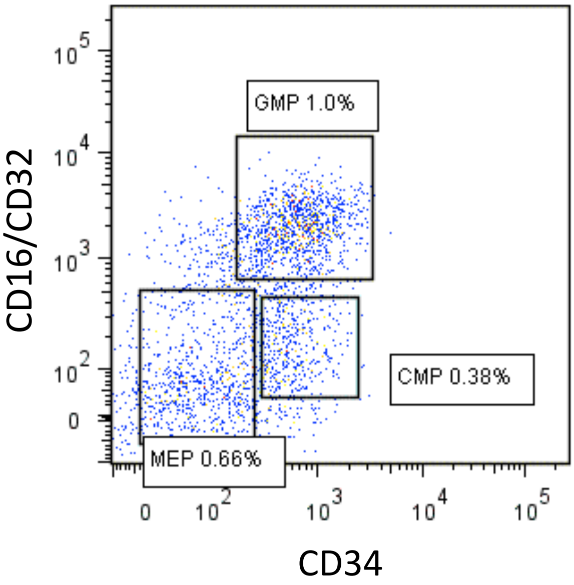

Representative flow cytometry for bone marrow cells of a *Uhrf2*$^{-/-}$ mouse. Cells were treated with Biotin-Lineage (CD3e, CD4, CD5, CD8a, CD11, B220, Ter119, and Gr1) followed by visualization with APC/Cy7-streptavidin and staining with perCP/Cy5.5-Sca-1, PE/Cy7-cKit, Alexa647-CD34, and PE-CD16/32. Using a FACSCanto II (BD), lineage$^-$Sca-1$^+$c-kit$^+$ (LSK) cells with hematopoietic stem and multipotent progenitor cells as well as the lineage$^-$Sca-1$^-$c-kit$^+$ fraction, containing committed progenitor cells, was identified. Common myeloid progenitor (CMP), granulocyte-macrophage progenitor (GMP), and megakaryocyte-erythroid progenitor (MEP) cells in the committed progenitor fraction were identified using fluorescence of CD34 and CD16/CD32.

Figure S2. Pie charts of genomic features in *Uhrf2*$^{-/-}$ LSK cells in CUT&Tag.

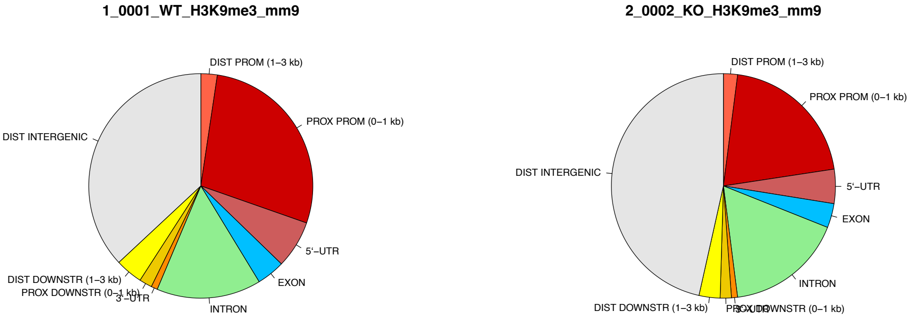

The location of peaks relative to genomic annotations is presented. As a control, randomly located “peaks” are run against the same genomic features database.
