# Supplementary material for: Impaired Repopulating Ability of Uhrf2−/− Hematopoietic Progenitor Cells in Mice
Source: Genes (Basel). 2023 Jul 27;14(8):1531. doi: 10.3390/genes14081531 (PMC10454722; doi:10.3390/genes14081531)
Supplement: Supplementary file 1 [file genes-14-01531-s001.zip › Supplemental materials/Figure S2.pdf]

Figure S2. Pie charts of genomic features in *Uhrf2*$^{-/-}$ LSK cells in CUT&Tag.

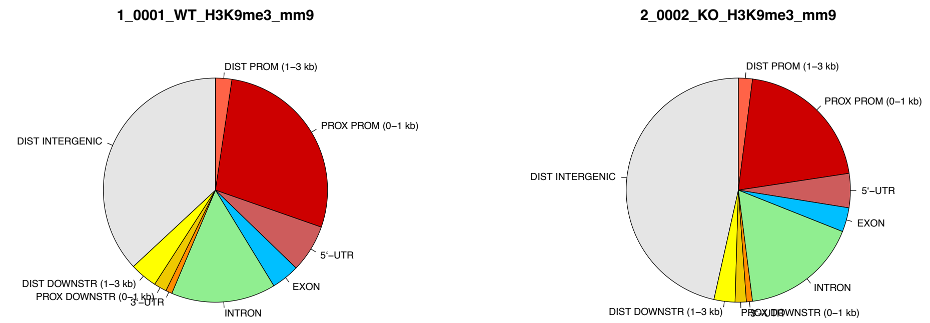

The location of peaks relative to genomic annotations is presented. As a control, randomly located “peaks” are run against the same genomic features database.
