# Supplementary material for: Impaired Repopulating Ability of Uhrf2−/− Hematopoietic Progenitor Cells in Mice
Source: Genes (Basel). 2023 Jul 27;14(8):1531. doi: 10.3390/genes14081531 (PMC10454722; doi:10.3390/genes14081531)
Supplement: Supplementary file 1 [file genes-14-01531-s001.zip › Supplemental materials/Table S1.pdf]

Table S1. Upregulated genes in *Uhrf2*$^{-/-}$ LSK cells in RNA sequencing.

No.	Gene Symbol	Entrez ID	Chromosome	Fold change (KO/WT)
1	Mybl1	17864	chr1	2.205687
2	1700034P13Rik	73331	chr1	2.6390388
3	Snhg6	73824	chr1	17.53908
4	Tram2	170829	chr1	7.548194
5	Kcnq5	226922	chr1	3.4004583
6	B3gat2	280645	chr1	3.729018
7	Arid5a	214855	chr1	2.0434375
8	Lman2l	214895	chr1	2.0404997
9	Cnnm3	94218	chr1	2.8392153
10	Ankrd39	109346	chr1	2.1982772
11	Unc50	67387	chr1	2.0538163
12	Mrpl30	107734	chr1	3.3629642
13	Hspd1	15510	chr1	2.0774598
14	Satb2	212712	chr1	2.9654424
15	Ppil3	70225	chr1	3.6097267
16	Nif3l1	65102	chr1	2.120928
17	Carf	241066	chr1	2.467191
18	Cyp20a1	77951	chr1	2.0783029
19	9530026F06Rik	77341	chr1	3.5678685
20	2810408I11Rik	69941	chr1	4.255011
21	Plekhm3	241075	chr1	2.1702394
22	Fn1	14268	chr1	2.6463652
23	Mreg	381269	chr1	2.0759504
24	Smarcal1	54380	chr1	3.0244858
25	Ttll4	67534	chr1	3.8326678
26	Cyp27a1	104086	chr1	2.0632362
27	Nhej1	75570	chr1	5.484591
28	Fbxo36	66153	chr1	5.750382
29	Snord82	80828	chr1	2.6575923
30	Ugt1a7c	394432	chr1	2.944653
31	Gbx2	14472	chr1	3.021903
32	Rnpepl1	108657	chr1	5.3754053
33	9430060I03Rik	100037260	chr1	3.1325345

34	D1Ertd622e	52392	chr1	2.0895526
35	Tmem185b	226351	chr1	2.8574748
36	Steap3	68428	chr1	2.713058
37	Ccnt2	72949	chr1	2.1044636
38	Yod1	226418	chr1	5.689087
39	Eif2d	16865	chr1	2.4402454
40	Srgap2	14270	chr1	2.0465786
41	Tmcc2	68875	chr1	2.1385703
42	Tmem183a	57439	chr1	16.218536
43	Ube2t	67196	chr1	5.7887907
44	Tnni1	21952	chr1	4.805928
45	Ddx59	67997	chr1	9.932252
46	Kif14	381293	chr1	3.0553591
47	Zbtb41	226470	chr1	2.072684
48	Tsen15	66637	chr1	8.986067
49	Ncf2	17970	chr1	2.1525297
50	Npl	74091	chr1	4.771479
51	Soat1	20652	chr1	2.262162
52	2810025M15Rik	69953	chr1	13.152842
53	4930523C07Rik	67647	chr1	5.2312994
54	4930562F07Rik	75255	chr1	8.005697
55	Serpinc1	11905	chr1	2.8077948
56	Snord47	100217446	chr1	10.215969
57	Mettl13	71449	chr1	3.3558986
58	Slc19a2	116914	chr1	6.196881
59	Blzf1	66352	chr1	3.3469505
60	Creg1	433375	chr1	2.1850054
61	Sh2d1b2	545378	chr1	3.1308796
62	Sh2d1b1	26904	chr1	2.7397928
63	Nos1ap	70729	chr1	2.002257
64	Dusp12	80915	chr1	6.701411
65	Ufc1	66155	chr1	5.540037
66	Pfdn2	18637	chr1	5.6397696
67	Alyref2	56009	chr1	4.345848
68	Cd48	12506	chr1	10.356822

69	Slamf6	30925	chr1	4.1638265
70	Ncstn	59287	chr1	2.212942
71	Pea15a	18611	chr1	2.4341457
72	Ccdc19	71870	chr1	2.546632
73	BC094916	545384	chr1	2.002699
74	Ifi204	15951	chr1	3.9528165
75	Mnda	381308	chr1	3.0536284
76	Ifi205	226695	chr1	2.0135372
77	Opn3	13603	chr1	3.6314564
78	Sccpdh	109232	chr1	17.094337
79	Adck3	67426	chr1	3.2378724
80	Lin9	72568	chr1	2.4921012
81	Cnih4	98417	chr1	4.7338467
82	Aida	108909	chr1	2.0237596
83	Taf1a	21339	chr1	3.1347156
84	Tatdn3	68972	chr1	4.55401
85	Nsl1	381318	chr1	2.2333174
86	Atf3	11910	chr1	2.5102124
87	Dtl	76843	chr1	2.0519118
88	Nek2	18005	chr1	2.412413
89	Traf5	22033	chr1	5.845829
90	Hsd11b1	15483	chr1	11.029
91	Suv39h2	64707	chr2	3.825286
92	Mcm10	70024	chr2	3.2322738
93	Nudt5	53893	chr2	6.721563
94	Sec61a2	57743	chr2	2.0821977
95	Fam188a	66960	chr2	2.3866658
96	Pter	19212	chr2	2.6898928
97	Nebl	74103	chr2	2.0960085
98	Gm13375	433408	chr2	6.1849003
99	Acbd5	74159	chr2	2.4963808
100	Spopl	76857	chr2	2.14403
101	Il1f9	215257	chr2	2.3874147
102	Mrpl41	107733	chr2	2.169895
103	Rnf208	68846	chr2	2.0081787

104	Ptgds	19215	chr2	4.877172
105	Fbxw5	30839	chr2	3.2635903
106	Traf2	22030	chr2	5.549851
107	Tmem141	51875	chr2	3.7214434
108	Card9	332579	chr2	2.2816126
109	Sdccag3	68112	chr2	12.353291
110	Surf4	20932	chr2	2.6826863
111	Fcnb	14134	chr2	2.1990263
112	6530402F18Rik	76220	chr2	2.8355207
113	Med27	68975	chr2	5.4768276
114	Coq4	227683	chr2	2.1555328
115	Wdr34	71820	chr2	2.2084577
116	Zdhhc12	66220	chr2	7.9111724
117	Fam73b	108958	chr2	3.0236647
118	Cstad	78617	chr2	9.174206
119	Exosc2	227715	chr2	2.1615894
120	Uck1	22245	chr2	2.8458626
121	Lcn2	16819	chr2	8.787018
122	Ptges2	96979	chr2	7.551383
123	Fpgs	14287	chr2	2.173485
124	Mir2861	100499514	chr2	5.572066
125	Tor2a	30933	chr2	3.073109
126	Hspa5	14828	chr2	2.17574
127	Zbtb26	320633	chr2	3.9733844
128	Ppp6c	67857	chr2	2.0763793
129	Gm13476	433424	chr2	7.973846
130	Arl5a	75423	chr2	3.2224128
131	Pkp4	227937	chr2	2.0381134
132	Gca	227960	chr2	3.3525128
133	Atp5g3	228033	chr2	2.1543365
134	Agps	228061	chr2	2.4922745
135	Plekha3	83435	chr2	3.7608323
136	Gm14461	329436	chr2	2.1222675
137	Cerkl	228094	chr2	11.532891
138	Tmx2	66958	chr2	10.561519

139	Timm10	30059	chr2	3.5085928
140	Slc39a13	68427	chr2	3.9120197
141	Acp2	11432	chr2	3.2344594
142	Ddb2	107986	chr2	5.9293194
143	Arfgap2	77038	chr2	2.5969348
144	Gyltl1b	228366	chr2	3.2393756
145	Pex16	18633	chr2	9.583265
146	1700029I15Rik	75641	chr2	5.2904115
147	Prdm11	100042784	chr2	5.9945383
148	2810002D19Rik	66457	chr2	2.0471137
149	Ldlrad3	241576	chr2	2.4239988
150	Cd59b	333883	chr2	4.259727
151	Depdc7	211896	chr2	9.407362
152	Dnajc24	99349	chr2	11.17238
153	Mettl15	76894	chr2	4.5299244
154	Kif18a	228421	chr2	3.6892514
155	Bahd1	228536	chr2	4.702327
156	Ccdc32	269336	chr2	3.1238956
157	Zfyve19	72008	chr2	2.5261347
158	Oip5	70645	chr2	9.742525
159	Nusap1	108907	chr2	2.89931
160	Ndufaf1	69702	chr2	2.1298776
161	Ganc	76051	chr2	2.3607018
162	4931402G19Rik	70933	chr2	3.0302584
163	Serinc4	574418	chr2	2.6428957
164	Bambi-ps1	81913	chr2	4.3452234
165	Gm14085	381417	chr2	2.2706013
166	Gatm	67092	chr2	2.8439403
167	AA467197	433470	chr2	11.711447
168	Galk2	69976	chr2	2.7688403
169	Atp8b4	241633	chr2	2.973513
170	Hdc	15186	chr2	3.689429
171	Ap4e1	108011	chr2	2.4247024
172	Polr1b	20017	chr2	3.1881948
173	Chchd5	66170	chr2	4.7973156

174	Sirpa	19261	chr2	2.2597852
175	4930473A02Rik	321014	chr2	3.0633397
176	Itpa	16434	chr2	7.87294
177	Rnf24	51902	chr2	2.5654647
178	Mkks	59030	chr2	3.2799075
179	Ndufaf5	69487	chr2	2.0309894
180	Snord17	100313519	chr2	5.342383
181	Gm561	228715	chr2	10.108854
182	Slc24a3	94249	chr2	2.1903348
183	Entpd6	12497	chr2	3.7064114
184	Nanp	67311	chr2	2.2629945
185	Sox12	20667	chr2	5.4430194
186	Id1	15901	chr2	3.3302112
187	Pdrg1	68559	chr2	2.1105306
188	Hck	15162	chr2	2.640617
189	Aar2	68295	chr2	2.5895867
190	Manbal	69161	chr2	3.4639754
191	Blcap	53619	chr2	6.349063
192	Ctnnbl1	66642	chr2	2.9547215
193	Slc32a1	22348	chr2	2.481264
194	Fam83d	71878	chr2	3.55341
195	Mybl2	17865	chr2	3.670039
196	Fitm2	228859	chr2	2.063751
197	Slpi	20568	chr2	3.9080083
198	Dnttip1	76233	chr2	2.0553634
199	Acot8	170789	chr2	5.737629
200	Neurl2	415115	chr2	6.020714
201	Elmo2	140579	chr2	2.1277611
202	5031425F14Rik	319684	chr2	3.9269955
203	B4galt5	56336	chr2	2.2885365
204	Slc9a8	77031	chr2	2.7598343
205	Tmem189	407243	chr2	3.3858345
206	Cebpb	12608	chr2	2.9664252
207	Mocs3	69372	chr2	3.1903224
208	Pfdn4	109054	chr2	2.8647993

209	Rae1	66679	chr2	2.078567
210	Rbm38	56190	chr2	3.400197
211	Ctsz	64138	chr2	2.1317837
212	Atp5e	67126	chr2	2.173344
213	Gm14326	665211	chr2	2.5451941
214	Zfp931	353208	chr2	5.694735
215	Ss18l1	269397	chr2	2.5957694
216	Gm6307	622283	chr2	3.0162737
217	Samd10	229011	chr2	3.1457956
218	Mrps28	66230	chr3	18.043753
219	Fabp5	16592	chr3	5.9747186
220	Car13	71934	chr3	4.3470054
221	Gm9733	751864	chr3	6.0641794
222	Cyp7b1	13123	chr3	2.0597892
223	Mtfr1	67472	chr3	2.5749273
224	Fndc3b	72007	chr3	2.035635
225	Cldn11	18417	chr3	2.1926193
226	4930429B21Rik	67576	chr3	5.1250095
227	Mrpl47	74600	chr3	2.5043607
228	Ccna2	12428	chr3	7.3445663
229	Bbs12	241950	chr3	4.9144387
230	Nudt6	229228	chr3	2.4971104
231	Gm5148	381438	chr3	13.61096
232	Hspa4l	18415	chr3	2.6797955
233	Gm7977	666202	chr3	3.340163
234	Ccrn4l	12457	chr3	2.8060288
235	Ndufc1	66377	chr3	6.5200343
236	Commd2	52245	chr3	2.8704827
237	Lekr1	624866	chr3	2.504118
238	Ift80	68259	chr3	2.3380606
239	Gm6634	625901	chr3	3.9569876
240	Ppid	67738	chr3	3.2584212
241	4930579G24Rik	75939	chr3	2.539547
242	Sh3d19	27059	chr3	3.390417
243	Rrnad1	229503	chr3	2.4998083

244	Tsacc	76927	chr3	30.794556
245	Smg5	229512	chr3	2.0957537
246	Slc25a44	229517	chr3	2.2203345
247	Sema4a	20351	chr3	11.105786
248	Ssr2	66256	chr3	2.1618044
249	1500004A13Rik	319830	chr3	2.1260467
250	Msto1	229524	chr3	3.1707942
251	Rusc1	72296	chr3	2.5942612
252	Scamp3	24045	chr3	2.17137
253	Krtcap2	66059	chr3	5.0645943
254	Dpm3	68563	chr3	7.509462
255	Efna4	13639	chr3	3.1308796
256	Adam15	11490	chr3	2.6309302
257	Zbtb7b	22724	chr3	3.4291847
258	Lenep	57275	chr3	13.357795
259	Flad1	319945	chr3	2.5862792
260	Pmvk	68603	chr3	3.7252295
261	4933434E20Rik	99650	chr3	2.2877886
262	Jtb	23922	chr3	4.584328
263	S100a1	20193	chr3	2.133574
264	S100a4	20198	chr3	4.564022
265	Gm128	229588	chr3	2.3066278
266	BC028528	229600	chr3	3.335976
267	Vps45	22365	chr3	2.477613
268	Sv2a	64051	chr3	2.2780206
269	Hist2h3c1	15077	chr3	6.488137
270	Hist2h2bb	319189	chr3	2.8398364
271	Gm15441	100038464	chr3	12.770718
272	Pex11b	18632	chr3	2.5844467
273	Wars2	70560	chr3	2.1921751
274	Gdap2	14547	chr3	8.888818
275	A130049A11Rik	100125931	chr3	2.0734715
276	I830077J02Rik	433638	chr3	3.3290524
277	Cept1	99712	chr3	2.24942
278	Gstm5	14866	chr3	5.485054

279	Gstm3	14864	chr3	7.336243
280	Gstm2	14863	chr3	2.3862865
281	Ampd2	109674	chr3	2.0256667
282	Atxn7l2	72522	chr3	3.7339718
283	Clcc1	229725	chr3	3.0468817
284	Gpsm2	76123	chr3	3.479587
285	Slc35a3	229782	chr3	2.4760847
286	Usp53	99526	chr3	2.2674308
287	Synpo2	118449	chr3	3.5060024
288	5730508B09Rik	70617	chr3	8.572321
289	Pitx2	18741	chr3	2.2088258
290	Hadh	15107	chr3	2.2112975
291	Sgms2	74442	chr3	3.0993402
292	Ppa2	74776	chr3	2.4361253
293	4930539J05Rik	319587	chr3	2.5325587
294	Pdlim5	56376	chr3	2.219339
295	Hs2st1	23908	chr3	3.0173264
296	Bcl10	12042	chr3	2.0139656
297	2410004B18Rik	66421	chr3	3.2650313
298	Cryz	12972	chr3	2.906564
299	Depdc1a	76131	chr3	3.7593167
300	Chd7	320790	chr4	2.2964244
301	Plekhf2	71801	chr4	2.5362492
302	Tmem64	100201	chr4	2.4493592
303	Fbxl4	269514	chr4	2.103419
304	Epha7	13841	chr4	3.38353
305	Nudt2	66401	chr4	7.576139
306	Stoml2	66592	chr4	2.702477
307	Msmp	100039672	chr4	5.261806
308	Grhpr	76238	chr4	2.329007
309	Tomm5	68512	chr4	2.3483975
310	Xpa	22590	chr4	11.56219
311	Tbc1d2	381605	chr4	2.2809427
312	Sec61b	66212	chr4	2.3018558
313	Invs	16348	chr4	2.390679

314	Tmem38b	52076	chr4	3.6052494
315	Gm12505	100415914	chr4	4.125565
316	Ptgr1	67103	chr4	2.8086298
317	Snx30	209131	chr4	2.2373295
318	Whrn	73750	chr4	2.8971624
319	Rraga	68441	chr4	2.3171194
320	Scarna8	100217448	chr4	15.405115
321	Pgm2	72157	chr4	3.238986
322	Sgip1	73094	chr4	2.2390747
323	Tctex1d1	67344	chr4	2.1301608
324	Yipf1	230584	chr4	5.100967
325	Tmem48	72787	chr4	3.4091465
326	Lrp8	16975	chr4	2.2657964
327	Magoh	17149	chr4	2.6830943
328	0610037L13Rik	74098	chr4	3.3089168
329	Orc1	18392	chr4	2.4932747
330	Kti12	100087	chr4	2.6623466
331	Uqcrh	66576	chr4	3.420363
332	Rad54l	19366	chr4	3.5259278
333	Tmem69	230657	chr4	2.7775652
334	C530005A16Rik	654318	chr4	3.739642
335	Tesk2	230661	chr4	4.355558
336	Btbd19	78611	chr4	3.6028283
337	Plk3	12795	chr4	3.0594134
338	Snord38a	100217424	chr4	34.397705
339	Dph2	67728	chr4	2.8235383
340	Ipo13	230673	chr4	3.400197
341	Hyi	68180	chr4	13.780291
342	Med8	80509	chr4	2.5119817
343	Elovl1	54325	chr4	2.918558
344	Lepre1	56401	chr4	2.936078
345	Ppih	66101	chr4	2.4806333
346	Ppcs	106564	chr4	2.0011485
347	Mfsd2a	76574	chr4	2.075118
348	Ndufs5	595136	chr4	2.8048747

349	Akirin1	68050	chr4	3.8779652
350	Mycbp	56309	chr4	7.3306866
351	1110065P20Rik	68920	chr4	10.538985
352	Cdca8	52276	chr4	2.0181053
353	Oscp1	230751	chr4	2.3385577
354	Lsm10	116748	chr4	2.4183192
355	Ncdn	26562	chr4	3.1142743
356	Yars	107271	chr4	2.0677152
357	Zbtb8os	67106	chr4	2.2393703
358	Eif3i	54709	chr4	2.0569305
359	Tmem39b	230770	chr4	2.980877
360	Sdc3	20970	chr4	2.3689399
361	Snora16a	100310813	chr4	17.669024
362	Snora61	100217440	chr4	23.56264
363	Rcc1	100088	chr4	2.2523403
364	Med18	67219	chr4	3.0505428
365	Rpa2	19891	chr4	2.450346
366	Lin28a	83557	chr4	2.6153643
367	Paqr7	71904	chr4	2.380348
368	Nipal3	74552	chr4	3.2404559
369	Lypla2	26394	chr4	3.8503516
370	E2f2	242705	chr4	2.3619955
371	Ephb2	13844	chr4	3.55498
372	Pqlc2	212555	chr4	2.266714
373	Rsg1	76166	chr4	3.9407604
374	Plekhm2	69582	chr4	3.1097238
375	Gm13154	433804	chr4	3.9754972
376	Zfp933	242747	chr4	8.909854
377	Miip	28010	chr4	8.36686
378	Plod1	18822	chr4	4.170656
379	Clcn6	26372	chr4	6.0433493
380	Masp2	17175	chr4	2.251216
381	Pex14	56273	chr4	2.1298773
382	Apitd1	69928	chr4	4.605423
383	Pgd	110208	chr4	2.0975866

384	Gpr157	269604	chr4	2.7398157
385	Dnajc11	230935	chr4	3.9346972
386	Acot7	70025	chr4	2.3947036
387	Wdr8	59002	chr4	2.2950506
388	Rer1	67830	chr4	2.351561
389	Ssu72	68991	chr4	3.1695454
390	Mrpl20	66448	chr4	5.216026
391	Tnfrsf4	22163	chr4	3.708263
392	Agrn	11603	chr4	2.6603355
393	Klhl17	231003	chr4	7.6184072
394	1700109H08Rik	77036	chr5	5.8857894
395	Mterf	545725	chr5	4.7987103
396	Slc25a40	319653	chr5	2.2347975
397	Abcb1b	18669	chr5	2.9961455
398	1700003C15Rik	69345	chr5	3.7756026
399	Tmem60	212090	chr5	2.5068061
400	6030443J06Rik	320719	chr5	2.7721028
401	Mir3096b	100628569	chr5	4.925555
402	Snord93	100217436	chr5	9.960567
403	Atg9b	213948	chr5	2.0581
404	1700096K18Rik	73571	chr5	3.571666
405	Emilin1	100952	chr5	2.391291
406	Cad	69719	chr5	2.2918167
407	Mpv17	17527	chr5	2.1065013
408	Tnip2	231130	chr5	2.063257
409	Sh3bp2	24055	chr5	2.3857393
410	Sh3tc1	231147	chr5	2.519339
411	Lyar	17089	chr5	2.8635564
412	9230114K14Rik	414108	chr5	2.0644958
413	Sepsecs	211006	chr5	5.4246674
414	C330024D21Rik	320479	chr5	5.8731766
415	Nfxl1	100978	chr5	2.4586828
416	Dcun1d4	100737	chr5	3.0426817
417	Scfd2	212986	chr5	2.415028
418	Chic2	74277	chr5	5.1787686

419	Clock	12753	chr5	4.606479
420	Exoc1	69940	chr5	3.1074202
421	Aasdh	231326	chr5	2.448226
422	Cenpc1	12617	chr5	2.4955778
423	Rufy3	52822	chr5	3.680718
424	Stbd1	52331	chr5	2.6971307
425	Anxa3	11745	chr5	2.0261073
426	Enoph1	67870	chr5	4.2119412
427	Cds1	74596	chr5	3.0997314
428	D930016D06Rik	100662	chr5	2.120062
429	Gbp8	76074	chr5	2.2498026
430	Btbd8	100503185	chr5	9.442334
431	Rpap2	231571	chr5	2.249411
432	Atp5k	11958	chr5	2.9388022
433	Pcgf3	69587	chr5	2.8059707
434	Crlf2	57914	chr5	2.1169405
435	Gtpbp6	107999	chr5	3.8675573
436	Fbrsl1	381668	chr5	3.1732657
437	Noc4l	100608	chr5	2.7730772
438	Srrd	70118	chr5	4.3684916
439	Hps4	192232	chr5	3.716535
440	2210016L21Rik	72357	chr5	2.2820113
441	Coq5	52064	chr5	2.2946894
442	Triap1	69076	chr5	3.0622332
443	Prkab1	19079	chr5	2.4714491
444	Oas1b	23961	chr5	2.0433385
445	Mapkapk5	17165	chr5	2.1411471
446	Acad12	338350	chr5	2.808629
447	Hvcn1	74096	chr5	3.819366
448	Tctn1	654470	chr5	2.261208
449	Vps33a	77573	chr5	3.0937479
450	Atp6v0a2	21871	chr5	3.5228567
451	Dhx37	208144	chr5	3.175178
452	Psph	100678	chr5	6.7808304
453	2410018M08Rik	71970	chr5	2.20509

454	Vkorc1l1	69568	chr5	14.667682
455	Cldn13	57255	chr5	5.8374815
456	Abhd11	68758	chr5	2.301028
457	Wbscr22	66138	chr5	2.3442478
458	Dnajc30	66114	chr5	4.0333643
459	Nsun5	100609	chr5	2.5862792
460	Tmem120a	215210	chr5	5.1666865
461	Mdh2	17448	chr5	2.8224883
462	Lrwd1	71735	chr5	2.1572542
463	Cldn15	60363	chr5	5.6724277
464	Ap1s1	11769	chr5	3.6005642
465	Slc12a9	83704	chr5	5.1142435
466	6330418K02Rik	69004	chr5	14.639058
467	Stag3	50878	chr5	3.011572
468	Fam20c	80752	chr5	2.1408079
469	Adap1	231821	chr5	3.7903996
470	Cox19	68033	chr5	5.862685
471	D830046C22Rik	320197	chr5	2.5743084
472	C130050O18Rik	319772	chr5	2.1351163
473	Mad1l1	17120	chr5	2.2288117
474	Snx8	231834	chr5	2.9021156
475	Gm3925	100042605	chr5	9.259724
476	E130309D02Rik	231868	chr5	3.742499
477	Kdelr2	66913	chr5	2.6080134
478	Aimp2	231872	chr5	4.5879016
479	Bhlha15	17341	chr5	2.0611737
480	Mtif3	76366	chr5	3.1503696
481	Gm8615	667410	chr5	3.716119
482	Hsph1	15505	chr5	2.4537377
483	Pon3	269823	chr6	3.651218
484	Slc25a13	50799	chr6	3.412731
485	Acn9	71238	chr6	8.111825
486	C1galt1	94192	chr6	2.361433
487	Mios	252875	chr6	2.1466646
488	Met	17295	chr6	4.1245246

489	Ing3	71777	chr6	3.1035366
490	Rbm28	68272	chr6	2.4444797
491	Zc3hc1	232679	chr6	2.6895137
492	Akr1b10	67861	chr6	3.9250593
493	3110062M04Rik	78412	chr6	3.5452518
494	Creb3l2	208647	chr6	2.1316614
495	Ubn2	320538	chr6	2.012723
496	1110001J03Rik	66117	chr6	2.0284545
497	Clec2l	665180	chr6	2.2479239
498	Hipk2	15258	chr6	2.15993
499	4930599N23Rik	75379	chr6	6.3243933
500	Ndufb2	68198	chr6	2.4540565
501	Mrps33	14548	chr6	2.4972951
502	Clec5a	23845	chr6	6.87288
503	Mgam	232714	chr6	7.1791153
504	Ephb6	13848	chr6	2.633689
505	Kel	23925	chr6	2.9723063
506	Pdia4	12304	chr6	2.0476449
507	Zfp746	69228	chr6	2.0361729
508	Hoxa4	15401	chr6	2.068636
509	Plekha8	231999	chr6	3.266199
510	Lsm5	66373	chr6	5.1663194
511	Mad2l1	56150	chr6	5.5550833
512	Gadd45a	13197	chr6	4.0270786
513	E230016M11Rik	320172	chr6	3.1892276
514	Il12rb2	16162	chr6	2.4828172
515	Dok1	13448	chr6	5.5746264
516	Mogs	57377	chr6	2.0008404
517	Mthfd2	17768	chr6	2.7992702
518	Npm3-ps1	108176	chr6	5.0856256
519	Pradc1	73327	chr6	13.770072
520	Pcyox1	66881	chr6	2.4609537
521	Pcbp1	23983	chr6	2.041698
522	1600020E01Rik	72012	chr6	4.978982
523	Gm5577	434064	chr6	2.0342505

524	Rpn1	103963	chr6	2.7101812
525	Eefsec	65967	chr6	2.9236207
526	Mcm2	17216	chr6	2.0424936
527	Aldh1l1	107747	chr6	3.1826549
528	Lsm3	67678	chr6	2.6623476
529	Slc25a26	67582	chr6	15.158414
530	Shq1	72171	chr6	4.035531
531	Sumf1	58911	chr6	2.3181622
532	Bhlhe40	20893	chr6	3.4110067
533	Edem1	192193	chr6	2.0921955
534	Ttll3	101100	chr6	2.3337643
535	Creld1	171508	chr6	8.191065
536	Emc3	66087	chr6	2.1332948
537	Atg7	74244	chr6	6.150747
538	Tsen2	381802	chr6	4.4262943
539	Snora7a	100217451	chr6	4.1502357
540	Rassf4	213391	chr6	4.289304
541	Pex26	74043	chr6	6.289344
542	Usp18	24110	chr6	7.255296
543	Clec4b1	69810	chr6	3.7558572
544	Clec4b2	381809	chr6	3.2478368
545	Clec4e	56619	chr6	2.639732
546	Cd163	93671	chr6	2.4989321
547	Leprel2	14789	chr6	3.7222247
548	Lpar5	381810	chr6	6.4787507
549	Scnn1a	20276	chr6	4.5915985
550	9630033F20Rik	319801	chr6	3.687049
551	Clec2g	70809	chr6	2.2641258
552	Klrb1f	232408	chr6	32.390312
553	Clec7a	56644	chr6	9.627078
554	Olr1	108078	chr6	4.1213503
555	Magohb	66441	chr6	3.716419
556	Loh12cr1	67774	chr6	4.690174
557	Recql	19691	chr6	2.1263595
558	B230216G23Rik	319552	chr6	3.5782616

559	Far2	330450	chr6	2.7256923
560	Rps4y2	66184	chr6	11.568618
561	Amn1	232566	chr6	4.865515
562	Mir294	100049712	chr7	2.1245255
563	Prpf31	68988	chr7	2.3458443
564	Mir3572	100628594	chr7	2.3164105
565	Leng1	69757	chr7	2.608013
566	Tsen34	66078	chr7	2.7046068
567	Hspbp1	66245	chr7	4.4626
568	Tmem150b	330460	chr7	4.3857965
569	Suv420h2	232811	chr7	2.5691035
570	Cox6b2	333182	chr7	4.5027733
571	Tmem238	664968	chr7	2.5122533
572	Isoc2a	664994	chr7	4.9552245
573	Zfp784	654801	chr7	8.315944
574	Zfp787	67109	chr7	2.332753
575	Zfp773	76373	chr7	2.0192788
576	Zfp418	232854	chr7	3.1726248
577	Zfp128	243833	chr7	2.7469733
578	Ube2m	22192	chr7	2.19947
579	Pla2g4c	232889	chr7	2.9122813
580	Tmem160	69094	chr7	3.012255
581	Ptgir	19222	chr7	3.4108117
582	Irf2bp1	272359	chr7	3.0605803
583	Qpctl	67369	chr7	2.7384138
584	Ercc1	13870	chr7	7.080799
585	Ercc2	13871	chr7	4.0924053
586	Ppp1r37	232947	chr7	3.5438294
587	Gemin7	69731	chr7	7.5306373
588	Tomm40	53333	chr7	3.0208294
589	Bcl3	12051	chr7	3.6512182
590	Zfp112	57745	chr7	2.239021
591	Smg9	71997	chr7	2.0011485
592	Cd177	68891	chr7	2.3671944
593	Atp1a3	232975	chr7	3.2374146

594	Dedd2	67379	chr7	2.3410375
595	Erf	13875	chr7	2.9531915
596	Lipe	16890	chr7	2.8725693
597	B3gnt8	232984	chr7	2.361882
598	Blvrb	233016	chr7	6.592476
599	Fbl	14113	chr7	2.369456
600	Eid2	386655	chr7	2.1907308
601	Timm50	66525	chr7	3.2078571
602	Med29	67224	chr7	2.282011
603	Paf1	54624	chr7	2.1170335
604	Rinl	320435	chr7	19.230421
605	Rasgrp4	233046	chr7	2.4668357
606	Kcnk6	52150	chr7	2.8762157
607	Yif1b	77254	chr7	2.1964362
608	Zfp383	73729	chr7	2.452124
609	Zfp568	243905	chr7	2.3771975
610	Wdr62	233064	chr7	2.129877
611	Nfkbid	243910	chr7	2.5942886
612	Lin37	75660	chr7	4.82808
613	Wbp7	75410	chr7	2.105591
614	Cox6b1	110323	chr7	2.3921778
615	Tmem147	69804	chr7	22.582447
616	Pdcd5	56330	chr7	4.2652826
617	1600014C10Rik	72244	chr7	2.3069055
618	AW146154	101835	chr7	2.5903993
619	Siglecg	243958	chr7	3.16113
620	Nkg7	72310	chr7	2.9796312
621	Zfp719	210105	chr7	3.5742073
622	Siglece	83382	chr7	4.1684737
623	Emc10	69683	chr7	2.1375802
624	Atf5	107503	chr7	2.3582392
625	Fuz	70300	chr7	5.4691057
626	Ap2a1	11771	chr7	2.669169
627	Rcn3	52377	chr7	2.0064898
628	Grwd1	101612	chr7	2.1758504

629	Tmem143	70209	chr7	2.7384136
630	Ccdc114	211535	chr7	4.656114
631	Saal1	78935	chr7	2.4752624
632	Nav2	78286	chr7	2.7156186
633	Htatip2	53415	chr7	2.4412215
634	Fancf	100040608	chr7	4.432481
635	Nipa2	93790	chr7	3.5433407
636	Mtmr10	233315	chr7	6.37725
637	Tarsl2	272396	chr7	2.1827939
638	Lysmd4	75099	chr7	3.4816344
639	Lrrc28	67867	chr7	2.0103467
640	Fam169b	434197	chr7	3.2703612
641	Det1	76375	chr7	2.8978643
642	Pex11a	18631	chr7	3.6544743
643	Anpep	16790	chr7	2.8153245
644	Sema4b	20352	chr7	2.5586314
645	Cib1	23991	chr7	13.006055
646	Hddc3	68695	chr7	4.034416
647	Fes	14159	chr7	2.515581
648	3110040N11Rik	67290	chr7	21.865023
649	Stard5	170460	chr7	3.0745065
650	Fam108c	70178	chr7	2.170795
651	I7Rn6	67669	chr7	2.3190174
652	Ndufc2	68197	chr7	2.548246
653	Myo7a	17921	chr7	3.6602397
654	Dgat2	67800	chr7	10.385731
655	Kcne3	57442	chr7	2.5969172
656	Lrrc51	69358	chr7	4.2335424
657	Fam160a2	74349	chr7	2.3759527
658	Arfip2	76932	chr7	2.7131104
659	Rrp8	101867	chr7	3.8875837
660	Taf10	24075	chr7	5.0164676
661	Ppfibp2	19024	chr7	2.898675
662	Lmo1	109594	chr7	5.8320312
663	Tmem9b	56786	chr7	2.555853

664	Zfp143	20841	chr7	2.4547038
665	Rras2	66922	chr7	3.922201
666	Plekha7	233765	chr7	2.6553502
667	Itpripl2	319622	chr7	2.1670983
668	Coq7	12850	chr7	2.9095645
669	Dcun1d3	233805	chr7	2.2364962
670	Lyrm1	73919	chr7	3.4964762
671	BC030336	233812	chr7	2.1440291
672	Igsf6	80719	chr7	2.891379
673	Plk1	18817	chr7	2.1531043
674	Nupr1	56312	chr7	6.389632
675	Bola2	66162	chr7	2.65185
676	Prr14	233895	chr7	3.4291844
677	Phkg2	68961	chr7	2.7149534
678	Gm166	233899	chr7	2.251863
679	4931431B13Rik	70973	chr7	2.0356338
680	Nsmce4a	67872	chr7	2.0504398
681	2310057M21Rik	68277	chr7	4.0634527
682	Lhpp	76429	chr7	7.8846726
683	Fam53b	77938	chr7	4.977249
684	Uros	22276	chr7	2.666596
685	Ppp2r2d	52432	chr7	2.5529866
686	Pwwp2b	101631	chr7	3.785518
687	Zfp511	69752	chr7	3.194816
688	Fuom	69064	chr7	7.1384053
689	Cyp2e1	13106	chr7	3.2240222
690	Ifitm6	213002	chr7	2.6389668
691	Sigirr	24058	chr7	2.3705668
692	Tmem80	71448	chr7	3.5923276
693	Slc25a22	68267	chr7	3.173452
694	Ctsd	13033	chr7	2.306682
695	Gm14492	677289	chr7	3.9062133
696	Mrgpre	244238	chr7	2.1860518
697	Nadsyn1	78914	chr7	2.3740423
698	Mcoln1	94178	chr8	4.8713827

699	C330021F23Rik	546049	chr8	9.5413685
700	Cd209c	170776	chr8	3.325863
701	Gm14378	100044509	chr8	7.68662
702	Shcbp1	20419	chr8	3.430885
703	Gm15350	100504249	chr8	2.067106
704	Mcf2l	17207	chr8	2.4162474
705	F10	14058	chr8	2.5210936
706	Pcid2	234069	chr8	2.0170026
707	Zfp828	101994	chr8	3.0793407
708	Erich1	234086	chr8	3.525502
709	Thsd1	56229	chr8	2.1851988
710	Ido2	209176	chr8	2.026615
711	5430421F17Rik	74489	chr8	2.402797
712	Rnf170	77733	chr8	3.1118336
713	Thap1	73754	chr8	2.458734
714	Gm8096	666422	chr8	2.2217932
715	Nrg1	211323	chr8	2.2945428
716	D8Ertd82e	244418	chr8	2.4640942
717	Tusc3	80286	chr8	2.1045194
718	Zdhhc2	70546	chr8	2.20147
719	Mtus1	102103	chr8	5.3380804
720	Ufsp2	192169	chr8	2.5015013
721	Mlf1ip	71876	chr8	5.3797097
722	Cpe	12876	chr8	2.6419601
723	Tma16	66282	chr8	2.1298776
724	Naf1	234344	chr8	7.838213
725	Sh2d4a	72281	chr8	2.579836
726	Atp13a1	170759	chr8	2.5579755
727	Pbx4	80720	chr8	2.3327224
728	2310045N01Rik	72368	chr8	2.1064723
729	Armc6	76813	chr8	2.3545697
730	Il12rb1	16161	chr8	3.1690345
731	Snora68	104370	chr8	22.771685
732	Ocel1	77090	chr8	3.3488283
733	Abhd8	64296	chr8	8.802044

734	Ano8	382014	chr8	2.5327106
735	Klf2	16598	chr8	3.4756784
736	Calr3	73316	chr8	5.364341
737	Il15	16168	chr8	4.166385
738	Ndufb7	66916	chr8	2.5148692
739	Ptger1	19216	chr8	9.536374
740	Lphn1	330814	chr8	2.1516106
741	Prkaca	18747	chr8	2.6243114
742	Gadd45gip1	102060	chr8	4.5907445
743	2310036O22Rik	68544	chr8	2.812424
744	Fbxw9	68628	chr8	5.09472
745	Orc6	56452	chr8	3.4840221
746	Rpgrip1l	244585	chr8	2.8680637
747	Bbs2	67378	chr8	2.7098866
748	Zfp319	79233	chr8	2.7992673
749	Gins3	78833	chr8	2.1150994
750	Fbxl8	50788	chr8	3.6612024
751	E2f4	104394	chr8	3.0810354
752	Atp6v0d1	11972	chr8	2.1055362
753	Mir1966	100316712	chr8	11.56686
754	Slc12a4	20498	chr8	2.1632218
755	Pla2g15	192654	chr8	2.7547138
756	Slc7a6os	66432	chr8	4.2972054
757	Nip7	66164	chr8	6.6356177
758	Hp	15439	chr8	4.854827
759	Mtss1l	244654	chr8	2.1859264
760	Mlycd	56690	chr8	2.3088586
761	Klhl36	234796	chr8	5.6654005
762	Foxl1	14241	chr8	2.2307472
763	Klhdc4	234825	chr8	4.699252
764	Slc7a5	20539	chr8	2.0621176
765	Cyba	13057	chr8	2.2254338
766	Mvd	192156	chr8	3.0435665
767	Cdk10	234854	chr8	2.166466
768	Urb2	382038	chr8	2.4318001

769	2810004N23Rik	66523	chr8	2.081237
770	Ntpcr	66566	chr8	2.5558524
771	Jrkl	77532	chr9	2.357705
772	Phxr4	18689	chr9	2.586379
773	Endod1	71946	chr9	2.4501593
774	Fut4	14345	chr9	2.2908452
775	Panx1	55991	chr9	5.435383
776	Zfp846	244721	chr9	3.4331715
777	5730577I03Rik	66662	chr9	2.945287
778	Mrpl4	66163	chr9	3.944618
779	Raver1	71766	chr9	2.0056973
780	Atg4d	235040	chr9	4.854915
781	Mir199a-1	387194	chr9	3.2608998
782	Tmem205	235043	chr9	2.0664
783	Ecsit	26940	chr9	2.6884885
784	Zfp599	235048	chr9	5.1024437
785	Ncapd3	78658	chr9	2.2767792
786	Prdm10	382066	chr9	2.408793
787	St3gal4	20443	chr9	12.734438
788	Rpusd4	71989	chr9	2.7150083
789	2610203C20Rik	100042464	chr9	2.2151763
790	Nlrx1	270151	chr9	7.7571864
791	Hinfp	102423	chr9	6.0092974
792	Hmbs	15288	chr9	11.181856
793	Trappc4	60409	chr9	8.215243
794	Amica1	270152	chr9	2.251585
795	Bud13	215051	chr9	2.312438
796	Gm4791	215467	chr9	3.3210018
797	Gm5617	434402	chr9	5.356624
798	Zbtb16	235320	chr9	2.220931
799	Timm8b	30057	chr9	9.242149
800	Pih1d2	72614	chr9	2.8349507
801	Fdxacb1	382137	chr9	7.3457923
802	Gm6981	629557	chr9	2.3642352
803	Npat	244879	chr9	2.1313543

804	AY074887	246735	chr9	4.640077
805	Fbxo22	71999	chr9	2.3203356
806	Rcn2	26611	chr9	2.4928572
807	Snupn	66069	chr9	3.9559038
808	Commd4	66199	chr9	2.5688736
809	Cox5a	12858	chr9	3.0069358
810	Mpi	110119	chr9	3.0956004
811	Senp8	71599	chr9	2.328583
812	2300009A05Rik	69478	chr9	17.607267
813	Zwilch	68014	chr9	3.22806
814	Snapc5	330959	chr9	3.3108501
815	Clpx	270166	chr9	3.8231966
816	Spg21	27965	chr9	2.7483354
817	Snx22	382083	chr9	6.4308352
818	Pigb	55981	chr9	9.80311
819	Rsl24d1	225215	chr9	2.6522794
820	Gcm1	14531	chr9	2.2579246
821	Ooep	67968	chr9	4.694372
822	Slc17a5	235504	chr9	4.9461274
823	Cox7a2	12866	chr9	3.7134552
824	Sh3bgrl2	212531	chr9	4.079089
825	Ttk	22137	chr9	2.6639514
826	Rwdd2a	69519	chr9	2.0538104
827	4922501C03Rik	382090	chr9	2.3422897
828	Tbc1d2b	67016	chr9	2.377235
829	Rnf7	19823	chr9	2.0155835
830	Rasa2	114713	chr9	2.6524942
831	Nmnat3	74080	chr9	3.1274774
832	Mrps22	64655	chr9	2.5724492
833	Pik3cb	74769	chr9	2.4296412
834	Cep70	68121	chr9	2.6613657
835	Dbr1	83703	chr9	2.158242
836	Nphp3	74025	chr9	2.1733441
837	Alas1	11655	chr9	2.132351
838	Poc1a	70235	chr9	12.880689

839	Dusp7	235584	chr9	5.063991
840	Acy1	109652	chr9	3.9535637
841	Mapkapk3	102626	chr9	2.1801376
842	Hyal3	109685	chr9	2.186055
843	Ifrd2	15983	chr9	2.088547
844	Nicn1	66257	chr9	4.9218683
845	Ndufaf3	66706	chr9	2.0264974
846	Slc25a20	57279	chr9	2.6951838
847	Uqcrc1	22273	chr9	2.2906044
848	Trex1	22040	chr9	2.3540752
849	Nme6	54369	chr9	11.8555
850	Ngp	18054	chr9	12.450708
851	Prss50	235631	chr9	2.6983664
852	Als2cl	235633	chr9	3.4451838
853	Mlh1	17350	chr9	2.583895
854	Fbxl2	72179	chr9	2.8415446
855	Stmn1-rs1	111186	chr9	7.5649605
856	Vill	22351	chr9	5.612506
857	Acaa1b	235674	chr9	2.1919909
858	Exog	208194	chr9	3.3469505
859	Cx3cr1	13051	chr9	2.6185424
860	Slc25a38	208638	chr9	7.059023
861	Higd1a	56295	chr9	3.4528377
862	Snrk	20623	chr9	4.6042347
863	Ano10	102566	chr9	5.045047
864	2010315B03Rik	630836	chr9	5.9833612
865	Lats1	16798	chr10	2.5669878
866	Shprh	268281	chr10	3.1259358
867	Stx11	74732	chr10	6.703428
868	Pex3	56535	chr10	9.275484
869	Adat2	66757	chr10	2.5017607
870	Reps1	19707	chr10	3.1511612
871	Ahi1	52906	chr10	2.1793401
872	C920009B18Rik	606736	chr10	2.2213645
873	Snord100	100529075	chr10	31.309866

874	Akap7	432442	chr10	2.7829409
875	Zufsp	72580	chr10	2.213428
876	Traf3ip2	103213	chr10	2.3258524
877	AA474331	213332	chr10	2.285021
878	Smpd2	20598	chr10	2.5236363
879	Bend3	331623	chr10	2.1875834
880	Mcm9	71567	chr10	2.6277275
881	Gm16998	100502920	chr10	3.6272385
882	Msl3l2	73390	chr10	3.3703554
883	Gja1	14609	chr10	2.8213956
884	Hsf2	15500	chr10	6.6675925
885	Sept10	103080	chr10	2.2189844
886	Sowahc	268301	chr10	2.2940354
887	P4ha1	18451	chr10	2.8279054
888	Micu1	216001	chr10	2.1769738
889	Pcbd1	13180	chr10	9.694131
890	2510003E04Rik	72320	chr10	11.189855
891	Ddx21	56200	chr10	2.0187984
892	Ado	211488	chr10	3.480694
893	Arid5b	71371	chr10	3.0068865
894	Cdk1	12534	chr10	2.5993783
895	Ube2d1	216080	chr10	4.9291444
896	Zwint	52696	chr10	3.4421597
897	Ddt	13202	chr10	7.32595
898	Mif	17319	chr10	2.5917115
899	Chchd10	103172	chr10	2.9209743
900	Vpreb3	22364	chr10	4.1502357
901	1700094J05Rik	67523	chr10	2.485146
902	Pcbp3	59093	chr10	3.6922786
903	Fstl3	83554	chr10	2.6499457
904	Prss57	73106	chr10	5.1200285
905	BC005764	216152	chr10	3.2550955
906	Prtn3	19152	chr10	2.1387727
907	Elane	50701	chr10	5.4163632
908	Polr2e	66420	chr10	7.3024364

909	Stk11	20869	chr10	2.210542
910	2310011J03Rik	66374	chr10	3.3469505
911	Mex3d	237400	chr10	2.0690238
912	Uqcr11	66594	chr10	4.1623874
913	Btbd2	208198	chr10	2.2312999
914	Timm13	30055	chr10	2.6340902
915	Thop1	50492	chr10	4.535498
916	Pias4	59004	chr10	2.344963
917	Dapk3	13144	chr10	5.772566
918	4930404N11Rik	432479	chr10	2.0542176
919	Tle6	114606	chr10	3.8083308
920	Zfp938	237411	chr10	2.0745559
921	1190007I07Rik	544717	chr10	3.9015057
922	Tdg	21665	chr10	2.302915
923	Eid3	66341	chr10	3.4230173
924	Ric8b	237422	chr10	2.9992151
925	AI597468	103266	chr10	2.0686452
926	Hsp90b1	22027	chr10	2.0565267
927	Nt5dc3	103466	chr10	2.1343398
928	Pmch	110312	chr10	2.3858309
929	Ccdc53	67282	chr10	10.870668
930	Dram1	71712	chr10	6.8027196
931	Arl1	104303	chr10	2.2519183
932	Ccdc38	237465	chr10	3.998077
933	Snrpf	69878	chr10	4.886189
934	Usp44	327799	chr10	2.1101828
935	Mrpl42	67270	chr10	2.0901904
936	4930430F08Rik	68281	chr10	4.4264607
937	Bbs10	71769	chr10	3.7597852
938	Lyz1	17110	chr10	2.163683
939	Gm15910	100504616	chr10	2.025899
940	Tmem5	216395	chr10	2.238544
941	Mirlet7i	387251	chr10	2.0995312
942	Cdk4	12567	chr10	2.0310118
943	Shmt2	108037	chr10	2.010325

944	Spryd4	66701	chr10	8.308413
945	Timeless	21853	chr10	2.4908912
946	Pan2	103135	chr10	3.7514043
947	Cs	12974	chr10	2.2326996
948	Nabp2	69917	chr10	6.1798277
949	Rnf41	67588	chr10	4.089029
950	Myl6b	216459	chr10	3.4828005
951	Zc3h10	103284	chr10	9.361557
952	8430429K09Rik	71523	chr11	6.079943
953	Sec14l2	67815	chr11	2.4341447
954	Osm	18413	chr11	2.1363034
955	Ap1b1	11764	chr11	2.5771291
956	Mrps24	64660	chr11	23.363447
957	Pold2	18972	chr11	2.8373024
958	Ddx56	52513	chr11	2.6222067
959	Myo1g	246177	chr11	2.017713
960	Tbrg4	21379	chr11	2.268101
961	Abca13	268379	chr11	2.1289043
962	Zpbp	53604	chr11	2.3987653
963	Pno1	66249	chr11	8.455454
964	Spred2	114716	chr11	3.1140366
965	Wdpcp	216560	chr11	3.5114787
966	B3gnt2	53625	chr11	2.2172513
967	Zrsr1	22183	chr11	2.4631631
968	Chac2	68044	chr11	2.1501222
969	Bod1	69556	chr11	3.9079437
970	Hba-a1	15122	chr11	4.6291094
971	Hba-a2	110257	chr11	4.2792397
972	Fndc9	320116	chr11	2.3144488
973	Rmnd5b	66089	chr11	2.1864858
974	Zcchc10	67966	chr11	13.629415
975	Uqcrq	22272	chr11	2.1741343
976	Gm12216	622459	chr11	3.0982666
977	Lyrm7	75530	chr11	3.883504
978	Slc36a2	246049	chr11	4.2099876

979	Sap30l	50724	chr11	3.8171828
980	Mrpl22	216767	chr11	7.357634
981	Gm12250	631323	chr11	6.736837
982	Guk1	14923	chr11	2.7522438
983	1700007J10Rik	69320	chr11	2.6909957
984	Srebf1	20787	chr11	2.2920816
985	Smcr7	237781	chr11	3.286096
986	Top3a	21975	chr11	2.3841217
987	Shmt1	20425	chr11	3.9678278
988	Tnfrsf13b	57916	chr11	5.4727783
989	Pigl	327942	chr11	4.8465333
990	Cenpv	73139	chr11	5.1777596
991	Zfp286	192651	chr11	3.5027106
992	Cox10	70383	chr11	4.7044473
993	Tmem220	338369	chr11	3.8897154
994	Cyb5d1	327951	chr11	11.675533
995	Lsmd1	78304	chr11	11.75366
996	Chrnb1	11443	chr11	4.1484365
997	Gps2	56310	chr11	2.343896
998	Phf23	78246	chr11	2.1926632
999	Mgl2	216864	chr11	9.057332
1000	0610010K14Rik	104457	chr11	2.140941
1001	Alox15	11687	chr11	4.4356284
1002	Nup88	19069	chr11	2.192875
1003	Mis12	67139	chr11	2.1643424
1004	Tax1bp3	76281	chr11	5.0376887
1005	Smg6	103677	chr11	2.478985
1006	Fam101b	76566	chr11	2.1749985
1007	Vps53	68299	chr11	3.9294057
1008	Nxn	18230	chr11	4.3830023
1009	Timm22	56322	chr11	4.2864027
1010	Git1	216963	chr11	2.0341985
1011	Eral1	57837	chr11	2.3158746
1012	Snord42a	100529076	chr11	100.7124
1013	Unc119	22248	chr11	5.186388

1014	Tmem199	195040	chr11	11.630301
1015	Tmem97	69071	chr11	2.2479148
1016	Rab11fip4	268451	chr11	2.2697635
1017	Rnf135	71956	chr11	3.3001392
1018	5730455P16Rik	70591	chr11	2.1876006
1019	Slfn4	20558	chr11	5.252806
1020	AA465934	613254	chr11	4.128898
1021	Ccl5	20304	chr11	2.0164988
1022	1100001G20Rik	66107	chr11	4.0744324
1023	Tada2a	217031	chr11	3.0162237
1024	Acaca	107476	chr11	2.1093712
1025	Pigw	70325	chr11	5.4157925
1026	Znhit3	448850	chr11	6.535671
1027	Bcas3	192197	chr11	2.4439607
1028	Tubd1	56427	chr11	2.1553166
1029	Rad51c	114714	chr11	2.7384138
1030	Bzrap1	207777	chr11	3.1203094
1031	Mpo	17523	chr11	2.9222393
1032	Mmd	67468	chr11	2.6826026
1033	Slc35b1	110172	chr11	9.904058
1034	Abi3	66610	chr11	4.7027917
1035	Gm11517	629750	chr11	5.095128
1036	Psmb3	26446	chr11	4.159778
1037	Mien1	103742	chr11	2.0538144
1038	Nr1d1	217166	chr11	2.4440103
1039	Gm12359	100037262	chr11	4.3575997
1040	Hsd17b1	15485	chr11	3.2521214
1041	Ifi35	70110	chr11	2.3161488
1042	Brca1	12189	chr11	2.2843409
1043	Rdm1	66599	chr11	2.6732132
1044	Lsm12	268490	chr11	2.8601174
1045	BC030867	217216	chr11	3.0426817
1046	Slc25a39	68066	chr11	2.0456717
1047	Nmt1	18107	chr11	2.6639566
1048	Nsf	18195	chr11	2.377642

1049	Cd79b	15985	chr11	2.707987
1050	Icam2	15896	chr11	3.3226085
1051	LOC100503496	100503496	chr11	3.1638587
1052	Abca9	217262	chr11	4.014499
1053	Nat9	66176	chr11	2.6969228
1054	Fdxr	14149	chr11	16.258425
1055	Ict1	68572	chr11	2.636991
1056	Nup85	445007	chr11	2.730913
1057	Mrps7	50529	chr11	9.264642
1058	1810032O08Rik	66293	chr11	2.0385969
1059	2810008D09Rik	76972	chr11	5.056371
1060	Pgs1	74451	chr11	10.165321
1061	Cant1	76025	chr11	3.7152746
1062	Chmp6	208092	chr11	2.3080916
1063	Azi1	12009	chr11	6.2169394
1064	1810043H04Rik	208501	chr11	7.8501186
1065	2310003H01Rik	71885	chr11	2.6417723
1066	Ccdc137	67291	chr11	2.399958
1067	Fam195b	192173	chr11	3.0216937
1068	Pycr1	209027	chr11	3.093693
1069	Myadml2	68515	chr11	2.0112543
1070	Dcxr	67880	chr11	14.969992
1071	Rfng	19719	chr11	5.8530483
1072	Fasn	14104	chr11	2.146914
1073	Slc16a3	80879	chr11	2.3545382
1074	B3gntl1	210004	chr11	4.6946726
1075	1700012B15Rik	74173	chr12	4.985698
1076	Adcy3	104111	chr12	2.6980915
1077	Ptrhd1	69709	chr12	4.8250465
1078	Gen1	209334	chr12	2.038376
1079	Mir3066	100526470	chr12	38.70221
1080	5730507C01Rik	236366	chr12	2.58587
1081	Taf1b	21340	chr12	3.3469493
1082	Cmpk2	22169	chr12	9.56936
1083	Acp1	11431	chr12	2.0622602

1084	Sh3yl1	24057	chr12	4.6259828
1085	4933406C10Rik	74076	chr12	8.0869875
1086	Ahr	11622	chr12	3.6705506
1087	Ankmy2	217473	chr12	2.8971639
1088	Immp2l	93757	chr12	3.6512182
1089	Gm2027	100039062	chr12	2.3210354
1090	Fam177a	73385	chr12	6.607971
1091	1700047I17Rik2	100101807	chr12	6.607971
1092	Mbip	217588	chr12	2.1034207
1093	Slc25a21	217593	chr12	3.6904976
1094	Gm527	217648	chr12	3.1715405
1095	Rps29	20090	chr12	2.8013656
1096	Lrr1	69706	chr12	6.8958616
1097	9330151L19Rik	414085	chr12	3.294433
1098	L2hgdh	217666	chr12	2.2856596
1099	Nin	18080	chr12	2.122713
1100	Dhrs7	66375	chr12	6.97378
1101	4930447C04Rik	75801	chr12	6.3535557
1102	Ppp1r36	210762	chr12	3.4390116
1103	Fntb	110606	chr12	5.866268
1104	Pigh	110417	chr12	3.5341916
1105	Vti1b	53612	chr12	4.0315547
1106	Cox16	66272	chr12	2.2611196
1107	Psen1	19164	chr12	2.178497
1108	Coq6	217707	chr12	4.230439
1109	Abcd4	19300	chr12	2.6402235
1110	Fcf1	73736	chr12	5.483412
1111	Acyp1	66204	chr12	6.885036
1112	0610007P14Rik	58520	chr12	2.019469
1113	Ahsa1	217737	chr12	2.758673
1114	Sptlc2	20773	chr12	4.5714693
1115	Slirp	380773	chr12	2.5555153
1116	Gm4027	100042776	chr12	2.4140413
1117	Adck1	72113	chr12	3.2838228
1118	Dio2	13371	chr12	2.2244818

1119	Spata7	104871	chr12	2.0317261
1120	Psmc1	19179	chr12	2.0580893
1121	D130020L05Rik	319760	chr12	2.2712255
1122	Lgmn	19141	chr12	2.5672615
1123	Itpk1	217837	chr12	2.5440288
1124	Ifi27l1	52668	chr12	2.312794
1125	Cdc42bpb	217866	chr12	2.4917104
1126	Pld4	104759	chr12	3.6439545
1127	Pfkp	56421	chr13	2.1937745
1128	Wdr37	207615	chr13	2.2181547
1129	Ero1lb	67475	chr13	2.44076
1130	Mir466i	100316665	chr13	3.6512182
1131	Gpr141	353346	chr13	8.265412
1132	Mir1896	100316752	chr13	2.3056867
1133	Hist1h4k	319160	chr13	3.9757707
1134	Hist1h1b	56702	chr13	2.2637553
1135	Hist1h3i	319153	chr13	3.0993094
1136	Zfp184	193452	chr13	2.9458692
1137	Hist1h2ag	319167	chr13	2.048569
1138	Hist1h2af	319173	chr13	2.0494432
1139	Hist1h3e	319151	chr13	5.088072
1140	Hist1h2bf	319180	chr13	3.9011497
1141	Hist1h2be	319179	chr13	4.14839
1142	Hist1h4c	319155	chr13	10.613547
1143	Hist1h2bb	319178	chr13	3.31497
1144	Hist1h3b	319150	chr13	3.458622
1145	Hist1h4a	326619	chr13	24.913239
1146	Lrrc16a	68732	chr13	3.3906736
1147	Aldh5a1	214579	chr13	2.6243114
1148	E2f3	13557	chr13	2.9106412
1149	Serpinb1c	380839	chr13	2.8484263
1150	Serpinb1b	282663	chr13	3.365469
1151	Cdyl	12593	chr13	5.54814
1152	Ly86	17084	chr13	4.957781
1153	Eef1e1	66143	chr13	7.723731

1154	Hivep1	110521	chr13	2.482049
1155	Sirt5	68346	chr13	9.565044
1156	Tpmt	22017	chr13	4.351611
1157	Fam120aos	68128	chr13	3.2980313
1158	1110007C09Rik	68480	chr13	3.2708871
1159	Sfxn1	14057	chr13	2.111499
1160	Arl10	56795	chr13	2.7852242
1161	Hk3	212032	chr13	2.3649776
1162	Zfp346	26919	chr13	2.5579028
1163	Mxd3	17121	chr13	2.8587055
1164	Dbn1	56320	chr13	2.0499196
1165	Fam193b	212483	chr13	2.0147536
1166	B4galt7	218271	chr13	10.510815
1167	4930528D03Rik	75223	chr13	4.8612022
1168	Etohd2	13996	chr13	3.6683586
1169	BC052688	432812	chr13	4.290965
1170	Zfp934	77117	chr13	2.2410777
1171	2010111I01Rik	72061	chr13	3.4414856
1172	Habp4	56541	chr13	2.3637075
1173	Cdc14b	218294	chr13	2.5185416
1174	Uqcrb	67530	chr13	2.9585238
1175	Zfp708	432769	chr13	3.6699278
1176	Zfp759	268670	chr13	2.163685
1177	Zfp748	212276	chr13	3.1681972
1178	Zfp71-rs1	235907	chr13	2.7344236
1179	Mtrr	210009	chr13	2.0490277
1180	Mrpl36	94066	chr13	6.1333313
1181	Glrx	93692	chr13	2.1155653
1182	Arsk	77041	chr13	2.4618082
1183	Lysmd3	80289	chr13	2.2045724
1184	Dhfr	13361	chr13	3.6776838
1185	Gfm2	320806	chr13	5.779662
1186	Hexb	15212	chr13	2.3449638
1187	Smn1	20595	chr13	2.868812
1188	Taf9	108143	chr13	2.3341665

1189	Zswim6	67263	chr13	4.387351
1190	Ndufaf2	75597	chr13	2.2820117
1191	Depdc1b	218581	chr13	2.0669253
1192	Nim1	245269	chr13	3.0881476
1193	Gm10406	100038847	chr14	2.7717345
1194	Il3ra	16188	chr14	3.3285265
1195	Nkiras1	69721	chr14	2.2086937
1196	Ube2e2	218793	chr14	4.156277
1197	1700112E06Rik	76633	chr14	8.22758
1198	Il17rb	50905	chr14	7.2734914
1199	Gnl3	30877	chr14	2.4324324
1200	Nt5dc2	70021	chr14	5.774017
1201	Mettl6	67011	chr14	2.0474162
1202	Dph3	105638	chr14	6.2534842
1203	Arhgap22	239027	chr14	2.6102717
1204	Ear10	93725	chr14	3.7889621
1205	Ear2	13587	chr14	9.969834
1206	Cdkn3	72391	chr14	15.237217
1207	Gm6498	624367	chr14	2.574606
1208	Tep1	21745	chr14	2.6143692
1209	Arhgef40	268739	chr14	2.008172
1210	Haus4	219072	chr14	3.4078038
1211	1700123O20Rik	58248	chr14	2.4215138
1212	Cebpe	110794	chr14	6.198535
1213	Ppp1r3e	105651	chr14	4.7787995
1214	Thtpa	105663	chr14	2.0420473
1215	Tm9sf1	74140	chr14	4.4411993
1216	Dhrs1	52585	chr14	7.764411
1217	Sdr39u1	654795	chr14	7.1098576
1218	Xpo4	57258	chr14	2.4644766
1219	Cab39l	69008	chr14	3.2188356
1220	Shisa2	219134	chr14	2.495946
1221	Arl11	219144	chr14	4.472742
1222	Dleu2	668253	chr14	8.797816
1223	Bin3	57784	chr14	2.2931433

1224	Sorbs3	20410	chr14	2.0673056
1225	Polr3d	67065	chr14	3.4800673
1226	Rb1	19645	chr14	2.687197
1227	Med4	67381	chr14	3.7819388
1228	Slc25a30	67554	chr14	3.2231371
1229	Nufip1	27275	chr14	2.8565407
1230	Dnajc15	66148	chr14	30.018763
1231	Gm5088	328451	chr14	9.105673
1232	Commd6	66200	chr14	2.0081692
1233	Fbxl3	50789	chr14	2.3354557
1234	Spry2	24064	chr14	2.5963492
1235	Ubac2	68889	chr14	4.392666
1236	Ptger4	19219	chr15	2.378096
1237	Ranbp3l	223332	chr15	2.5140777
1238	Fam134b	66270	chr15	2.3869028
1239	Fam105a	223433	chr15	2.2805195
1240	Ctnnd2	18163	chr15	2.4911187
1241	Fam173b	68073	chr15	14.846171
1242	9430069I07Rik	77358	chr15	7.8578763
1243	BC030476	239368	chr15	2.6370735
1244	Utp23	78581	chr15	2.3283834
1245	Wdyhv1	76773	chr15	7.441586
1246	Tatdn1	69694	chr15	10.693623
1247	E430025E21Rik	223593	chr15	3.9772196
1248	Asap1	13196	chr15	2.027214
1249	Tmem71	213068	chr15	2.6435246
1250	Zfat	380993	chr15	2.5201344
1251	Chrac1	93696	chr15	13.211503
1252	Bop1	12181	chr15	2.0042138
1253	Adck5	268822	chr15	3.9682775
1254	Recql4	79456	chr15	2.2420049
1255	Csf2rb	12983	chr15	2.6958368
1256	Tst	22117	chr15	9.606888
1257	Lgals1	16852	chr15	2.4741802
1258	Ankrd54	223690	chr15	2.205944

1259	Tmem184b	223693	chr15	2.724984
1260	Cby1	73739	chr15	3.5751512
1261	Dnalc4	54152	chr15	6.649937
1262	Snord43	100302600	chr15	12.619708
1263	Tab1	66513	chr15	3.540393
1264	Rangap1	19387	chr15	2.121813
1265	Pmm1	29858	chr15	6.6652436
1266	Ccdc134	76457	chr15	8.987613
1267	Tnfrsf13c	72049	chr15	2.5714989
1268	Cenpm	66570	chr15	5.3753057
1269	Rnu12	104307	chr15	21.415215
1270	A4galt	239559	chr15	3.658082
1271	1700001L05Rik	69291	chr15	3.3295913
1272	Cdpf1	72355	chr15	2.7019012
1273	Gtse1	29870	chr15	5.0107136
1274	Creld2	76737	chr15	2.1197824
1275	Abcd2	26874	chr15	4.7130365
1276	Yaf2	67057	chr15	5.6479783
1277	E330033B04Rik	319722	chr15	2.2478604
1278	Rpap3	71919	chr15	7.9947124
1279	Tmem106c	380967	chr15	2.477614
1280	Senp1	223870	chr15	2.1314015
1281	Rnd1	223881	chr15	2.3481266
1282	Fkbp11	66120	chr15	9.47035
1283	Rhebl1	69159	chr15	15.738739
1284	Mcrs1	51812	chr15	2.8387077
1285	6030408B16Rik	77717	chr15	3.0192788
1286	Espl1	105988	chr15	2.2748795
1287	Map3k12	26404	chr15	7.7238827
1288	Tarbp2	21357	chr15	3.551446
1289	Itga5	16402	chr15	2.9926138
1290	Zfp597	71063	chr16	3.8659952
1291	Vasn	246154	chr16	3.30301
1292	Nmral1	67824	chr16	2.245876
1293	Nagpa	27426	chr16	2.9183462

1294	Mettl22	239706	chr16	4.188167
1295	Nubp1	26425	chr16	2.4051068
1296	Gm4262	100043147	chr16	2.7351918
1297	3110001I22Rik	66598	chr16	5.562104
1298	2310008H04Rik	224008	chr16	2.3070183
1299	Igll1	16136	chr16	4.307659
1300	Thap7	69009	chr16	4.5640235
1301	Car15	80733	chr16	3.7145555
1302	Ranbp1	19385	chr16	2.3553288
1303	Comt	12846	chr16	4.1205826
1304	Sept5	18951	chr16	2.097557
1305	Cdc45	12544	chr16	2.4924638
1306	2510002D24Rik	72307	chr16	2.4050443
1307	Abcc5	27416	chr16	2.3088589
1308	Dvl3	13544	chr16	5.3014584
1309	Camk2n2	73047	chr16	2.5056806
1310	Tctex1d2	66061	chr16	3.9230165
1311	Tfrc	22042	chr16	2.2290673
1312	Iqcg	69707	chr16	3.851361
1313	Umps	22247	chr16	2.143726
1314	Ccdc14	239839	chr16	2.9073567
1315	Pdia5	72599	chr16	3.7607555
1316	Hspbap1	66667	chr16	2.2895675
1317	Parp9	80285	chr16	4.982844
1318	Slc15a2	57738	chr16	5.157817
1319	Polq	77782	chr16	3.937157
1320	Rabl3	67657	chr16	3.3149219
1321	Timmdc1	76916	chr16	5.25537
1322	Arhgap31	12549	chr16	2.610817
1323	Upk1b	22268	chr16	4.4388027
1324	BC002163	170658	chr16	2.8553624
1325	C330027C09Rik	224171	chr16	2.4958463
1326	5330426P16Rik	68190	chr16	2.67756
1327	Nit2	52633	chr16	2.5473588
1328	Filip1l	78749	chr16	2.7801654

1329	Crybg3	224273	chr16	2.90147
1330	Btg3	12228	chr16	2.296734
1331	Usp16	74112	chr16	2.429472
1332	Cct8	12469	chr16	2.4052083
1333	Ifngr2	15980	chr16	2.6523
1334	Mrps6	121022	chr16	8.236622
1335	Rcan1	54720	chr16	2.2820115
1336	Psmg1	56088	chr16	5.6230655
1337	Prdm15	114604	chr16	3.1777492
1338	Tiam2	24001	chr17	3.8218594
1339	Tfb1m	224481	chr17	9.899589
1340	Ldhal6b	106557	chr17	3.3345494
1341	Synj2	20975	chr17	2.1380358
1342	Acat3	224530	chr17	2.2545085
1343	Unc93a	381058	chr17	2.439654
1344	1600012H06Rik	67912	chr17	3.0976143
1345	Zfp51	22709	chr17	3.1620924
1346	2210404O09Rik	70081	chr17	3.5389004
1347	Zfp944	319615	chr17	2.8380876
1348	Zfp758	224598	chr17	2.0042052
1349	Hcfc1r1	353502	chr17	8.864743
1350	Tceb2	67673	chr17	19.78715
1351	Atp6v0c	11984	chr17	2.6283267
1352	Mlst8	56716	chr17	5.8850493
1353	Slc9a3r2	65962	chr17	3.0046458
1354	Ndufb10	68342	chr17	33.162674
1355	Hagh	14651	chr17	4.472742
1356	Narfl	67563	chr17	3.231375
1357	Haghl	68977	chr17	2.2952788
1358	Wdr24	268933	chr17	3.506053
1359	Stub1	56424	chr17	2.0179965
1360	Wdr90	106618	chr17	2.2285807
1361	Gm10012	100043160	chr17	7.8356123
1362	Nme4	56520	chr17	2.9716864
1363	Kifc5b	16580	chr17	2.0831602

1364	Taf11	68776	chr17	2.8513064
1365	Zfp523	224656	chr17	3.0471852
1366	Fance	72775	chr17	2.4261382
1367	BC004004	80748	chr17	3.858674
1368	Ftsjd2	74157	chr17	2.0658557
1369	Glo1	109801	chr17	2.0667272
1370	1700097N02Rik	67522	chr17	5.8078747
1371	Rrp1b	72462	chr17	2.0177464
1372	Zfp414	328801	chr17	2.0227304
1373	March2	224703	chr17	3.8531523
1374	Angptl4	57875	chr17	3.1730824
1375	Kank3	80880	chr17	2.1790557
1376	Kifc1	100502766	chr17	2.5918126
1377	AA388235	433100	chr17	3.5743577
1378	Slc39a7	14977	chr17	2.338524
1379	H2-DMb2	15000	chr17	5.222116
1380	Gpsm3	106512	chr17	2.0994503
1381	Lsm2	27756	chr17	4.198901
1382	Aif1	11629	chr17	2.651408
1383	Mrps18b	66973	chr17	3.6680229
1384	A930015D03Rik	77810	chr17	2.5503953
1385	Gm6623	625785	chr17	4.861453
1386	Rpp21	67676	chr17	7.4953265
1387	Aars2	224805	chr17	2.7426586
1388	Slc29a1	63959	chr17	4.8226504
1389	Mrps18a	68565	chr17	13.219274
1390	Tjap1	74094	chr17	4.615407
1391	Mrpl2	27398	chr17	3.7816188
1392	Rrp36	224823	chr17	2.001853
1393	Tbcc	72726	chr17	3.5438304
1394	Mrps10	64657	chr17	3.035605
1395	Tfeb	21425	chr17	3.02129
1396	Zfp959	224893	chr17	2.198246
1397	Ccdc94	72886	chr17	2.293713
1398	D17Wsu104e	28106	chr17	11.515382

1399	Dpp9	224897	chr17	4.9885817
1400	Kdm4b	193796	chr17	2.0877976
1401	Ndufa11	69875	chr17	2.2911294
1402	Alkbh7	66400	chr17	2.8629968
1403	Gtf2f1	98053	chr17	2.0261188
1404	Tnfsf9	21950	chr17	3.19197
1405	C3	12266	chr17	2.1329818
1406	Emr1	13733	chr17	2.0898256
1407	Lclat1	225010	chr17	2.0774176
1408	Xdh	22436	chr17	7.597756
1409	Dpy30	66310	chr17	4.0848036
1410	Slc30a6	210148	chr17	2.094081
1411	Thumpd2	72167	chr17	2.2378445
1412	Thada	240174	chr17	2.4793198
1413	Mettl4	76781	chr17	4.5588536
1414	Rbbp8	225182	chr18	4.837864
1415	Ttc39c	72747	chr18	2.4061708
1416	Psma8	73677	chr18	6.9571
1417	Cdh2	12558	chr18	2.0742247
1418	B4galt6	56386	chr18	4.7444053
1419	A830052D11Rik	402767	chr18	8.686113
1420	Pkd2l2	53871	chr18	2.0587661
1421	Kif20a	19348	chr18	3.0302994
1422	Nrg2	100042150	chr18	6.5582523
1423	Ndufa2	17991	chr18	6.174857
1424	Rell2	225392	chr18	2.1514523
1425	Lars	107045	chr18	2.1323178
1426	Stk32a	269019	chr18	2.1845276
1427	Ticam2	225471	chr18	2.8296936
1428	Ap3s1	11777	chr18	2.251584
1429	G630055G22Rik	414127	chr18	2.2725031
1430	Gm5506	433182	chr18	2.2810743
1431	9430076G02Rik	77433	chr18	2.162668
1432	Slc26a2	13521	chr18	2.0909595
1433	Grpel2	17714	chr18	2.0603533

1434	1500015A07Rik	68982	chr18	2.3935106
1435	Txnl1	53382	chr18	2.525563
1436	Fech	14151	chr18	2.150162
1437	Malt1	240354	chr18	2.142699
1438	Pmaip1	58801	chr18	4.746584
1439	Stard6	170461	chr18	4.455141
1440	Me2	107029	chr18	2.2129712
1441	Cxxc1	74322	chr18	2.1335995
1442	Haus1	225745	chr18	2.1356184
1443	Pqlc1	66943	chr18	4.5590715
1444	Mrpl21	353242	chr19	2.8224888
1445	1810055G02Rik	72056	chr19	2.086144
1446	Tcirg1	27060	chr19	2.2350411
1447	Ndufs8	225887	chr19	2.627096
1448	Unc93b1	54445	chr19	2.2991683
1449	Acy3	71670	chr19	3.6808133
1450	Doc2g	60425	chr19	4.2757716
1451	Cdk2ap2	52004	chr19	10.186529
1452	Ssh3	245857	chr19	2.0465102
1453	Ankrd13d	68423	chr19	2.0439544
1454	Rce1	19671	chr19	4.537031
1455	Rbm14	56275	chr19	2.3075695
1456	Mrpl11	66419	chr19	2.5102134
1457	Rab1b	76308	chr19	2.8294756
1458	D330050I16Rik	414115	chr19	4.193912
1459	Mus81	71711	chr19	2.1851988
1460	Sssca1	56390	chr19	15.728783
1461	Vps51	68505	chr19	6.76878
1462	Sac3d1	66406	chr19	3.194815
1463	Gpr137	107173	chr19	9.351905
1464	Dnajc4	57431	chr19	8.710555
1465	Macrod1	107227	chr19	4.6307397
1466	Snhg1	83673	chr19	3.7583055
1467	Tmem223	66836	chr19	2.1298754
1468	Taf6l	225895	chr19	2.396577

1469	B3gat3	72727	chr19	2.6862528
1470	Fads2	56473	chr19	2.0622633
1471	Tmem216	68642	chr19	7.6945906
1472	Dak	225913	chr19	2.12811
1473	Ms4a4c	64380	chr19	3.9394717
1474	Ms4a6d	68774	chr19	4.2304554
1475	Ms4a3	170813	chr19	2.7346413
1476	Oosp1	170834	chr19	2.657482
1477	Dtx4	207521	chr19	2.6270173
1478	Cntf	12803	chr19	5.25754
1479	E030024N20Rik	595139	chr19	4.7955313
1480	Mir1192	100316672	chr19	11.52462
1481	Fxn	14297	chr19	2.1620424
1482	Cbwd1	226043	chr19	2.9795332
1483	Ppapdc2	74411	chr19	3.7153397
1484	1700018L02Rik	67329	chr19	3.8640127
1485	Rcl1	59028	chr19	2.0326881
1486	Insl6	27356	chr19	8.652613
1487	Minpp1	17330	chr19	3.0577002
1488	Papss2	23972	chr19	2.1910853
1489	Rnls	67795	chr19	2.193587
1490	Stambpl1	76630	chr19	3.9356725
1491	Arhgap19	71085	chr19	2.2087746
1492	Mms19	72199	chr19	2.365013
1493	Ubtd1	226122	chr19	4.9085307
1494	Entpd7	93685	chr19	2.3556244
1495	Cox15	226139	chr19	2.0516162
1496	Mrpl43	94067	chr19	3.6953242
1497	Npm3	18150	chr19	2.652395
1498	Pprc1	226169	chr19	2.7047126
1499	Gbf1	107338	chr19	2.6103306
1500	Sfxn2	94279	chr19	3.6683488
1501	2010012O05Rik	66439	chr19	4.8187575
1502	Taf5	226182	chr19	4.014593
1503	Obfc1	108689	chr19	3.801068

1504	Nhlrc2	66866	chr19	2.0600455
1505	A630007B06Rik	213993	chr19	4.8682914
1506	D19Ertd737e	76539	chr19	4.490998
1507	Grk5	14773	chr19	2.1412964
1508	Csf2ra	12982	chr19	3.8335903
1509	Suv39h1	20937	chrX	2.029565
1510	Ftsj1	54632	chrX	2.446589
1511	Xk	22439	chrX	2.1136327
1512	2900008C10Rik	72937	chrX	2.0995312
1513	Atp6ap2	70495	chrX	2.5757587
1514	Gm5124	331392	chrX	4.390156
1515	Ndufa1	54405	chrX	9.235437
1516	C1galt1c1	59048	chrX	6.9438343
1517	6030498E09Rik	77883	chrX	5.5768795
1518	Aifm1	26926	chrX	3.49773
1519	Xlr	22441	chrX	4.8419385
1520	Mtap7d3	320923	chrX	4.0225363
1521	Mtm1	17772	chrX	4.5640798
1522	Nsdhl	18194	chrX	3.4462318
1523	Xlr4c	72891	chrX	6.1565814
1524	Ssr4	20832	chrX	3.2307
1525	Ubl4	27643	chrX	2.6103008
1526	Mtcp1	17763	chrX	3.1700497
1527	5430427O19Rik	71398	chrX	2.140591
1528	Klhl15	236904	chrX	6.926536
1529	Apoo	68316	chrX	3.3773742
1530	Las1l	76130	chrX	2.889014
1531	Gm14812	100038584	chrX	3.8795683
1532	Cxcr3	12766	chrX	2.2196536
1533	Pin4	69713	chrX	2.6427178
1534	Hdac8	70315	chrX	2.1430643
1535	Uprt	331487	chrX	2.9122813
1536	Itm2a	16431	chrX	2.041635
1537	2810403D21Rik	69964	chrX	4.439307
1538	Sytl4	27359	chrX	3.354514

1539	Nxt2	237082	chrX	2.0233834
1540	Ribc1	66611	chrX	3.5549831
1541	Sat1	20229	chrX	7.231999
1542	Map3k15	270672	chrX	3.0272572
1543	Rai2	24004	chrX	2.2770033
1544	Ap1s2	108012	chrX	2.595444
1545	Car5b	56078	chrX	2.9436061
1546	Siah1b	20438	chrX	2.7384136
1547	Fancb	237211	chrX	6.8824286
1548	Mid1	17318	chrX	2.7978764
