# Supplementary material for: Impaired Repopulating Ability of Uhrf2−/− Hematopoietic Progenitor Cells in Mice
Source: Genes (Basel). 2023 Jul 27;14(8):1531. doi: 10.3390/genes14081531 (PMC10454722; doi:10.3390/genes14081531)
Supplement: Supplementary file 1 [file genes-14-01531-s001.zip › Supplemental materials/Table S2.pdf]

Table S2. Downregulated genes in *Uhrf2*$^{-/-}$ LSK cells in RNA sequencing.

No.	Gene Symbol	Entrez ID	Chromosome	Fold change (KO/WT)
1	Atp6v1h	108664	chr1	-2.2679286
2	Pcmtd1	319263	chr1	-3.985097
3	Lactb2	212442	chr1	-2.987795
4	Rdh10	98711	chr1	-5.4091535
5	D030040B21Rik	329101	chr1	-3.0656853
6	Crispld1	83691	chr1	-6.6455493
7	Tmem14a	75712	chr1	-16.175089
8	Fam135a	68187	chr1	-3.758557
9	Lmbrd1	68421	chr1	-3.1465876
10	Gm20172	100504323	chr1	-3.0504358
11	1700001G17Rik	67503	chr1	-5.2585187
12	Dst	13518	chr1	-2.8449838
13	Ptpn18	19253	chr1	-5.996556
14	Hs6st1	50785	chr1	-6.2848525
15	Neurl3	214854	chr1	-2.650127
16	Cnnm4	94220	chr1	-2.3378723
17	4930594C11Rik	77633	chr1	-2.8882585
18	Lipt1	623661	chr1	-16.596134
19	Mitd1	69028	chr1	-4.2135572
20	Txndc9	98258	chr1	-2.3687425
21	Aff3	16764	chr1	-2.1146047
22	Pdcl3	68833	chr1	-2.1533055
23	Il1r2	16178	chr1	-3.3457386
24	Bivm	246229	chr1	-7.0881
25	Nabp1	109019	chr1	-3.2469513
26	Tmem194b	227094	chr1	-2.0582533
27	Inpp1	16329	chr1	-2.7338269
28	4930558J18Rik	75323	chr1	-3.2181044
29	1700066M21Rik	73467	chr1	-3.1445615
30	9430016H08Rik	68115	chr1	-8.410511
31	Stradb	227154	chr1	-2.1333873
32	Ctla4	12477	chr1	-12.352106
33	Gm11602	100380944	chr1	-4.1228

34	Ikzf2	22779	chr1	-3.2301235
35	Pnkd	56695	chr1	-2.4019494
36	Tmbim1	69660	chr1	-2.0627317
37	Zfp142	77264	chr1	-3.5061567
38	Bcs1l	66821	chr1	-5.4309626
39	Stk11ip	71728	chr1	-4.968084
40	Ap1s3	252903	chr1	-4.33346
41	Serpine2	20720	chr1	-2.1312947
42	Fam124b	241128	chr1	-3.1423287
43	Pid1	98496	chr1	-3.2401319
44	Gm7609	665378	chr1	-2.3009558
45	Snora75	100303740	chr1	-2.7388117
46	Sag	20215	chr1	-2.2759895
47	Cops8	108679	chr1	-2.1010525
48	Rab17	19329	chr1	-5.514309
49	Otos	260301	chr1	-6.1096096
50	Dusp28	67446	chr1	-6.9496293
51	Capn10	23830	chr1	-3.6517491
52	Pam	18484	chr1	-5.1461873
53	Phlpp1	98432	chr1	-2.1141708
54	2900060B14Rik	68204	chr1	-2.817063
55	Insig2	72999	chr1	-2.3799326
56	Daf2	13137	chr1	-3.6916072
57	Cd55	13136	chr1	-4.876078
58	Il10	16153	chr1	-6.850036
59	Dyrk3	226419	chr1	-2.7177444
60	Ctse	13034	chr1	-5.3685994
61	Rab7l1	226422	chr1	-4.300948
62	Tmem81	74626	chr1	-2.3127747
63	Snrpe	20643	chr1	-2.997829
64	Lax1	240754	chr1	-2.0595868
65	Btg2	12227	chr1	-2.07537
66	Klhl12	240756	chr1	-5.332986
67	Syt2	20980	chr1	-6.390402
68	Shisa4	77552	chr1	-5.9145126

69	Zfp281	226442	chr1	-2.6730804
70	Cfhr1	50702	chr1	-2.7467382
71	Rgs2	19735	chr1	-2.1819117
72	Pla2g4a	18783	chr1	-2.0821786
73	Swt1	66875	chr1	-2.46044
74	Rnf2	19821	chr1	-2.599194
75	Fam129a	63913	chr1	-5.173311
76	Rgl1	19731	chr1	-2.684742
77	Shcbp1l	71836	chr1	-2.8170636
78	Rgs8	67792	chr1	-2.3043494
79	Glul	14645	chr1	-2.660857
80	Ier5	15939	chr1	-3.0684836
81	Mr1	15064	chr1	-5.6104183
82	Xpr1	19775	chr1	-2.186786
83	BC026585	226527	chr1	-2.9256947
84	Rabgap1l	29809	chr1	-2.5737472
85	Zbtb37	240869	chr1	-2.265732
86	Suco	226551	chr1	-2.4700592
87	Vamp4	53330	chr1	-3.859816
88	Mettl18	69962	chr1	-3.7560844
89	Selp	20344	chr1	-2.064643
90	F5	14067	chr1	-2.296672
91	Dcaf6	74106	chr1	-4.7180624
92	Mpzl1	68481	chr1	-2.9043984
93	Tada1	27878	chr1	-2.4737656
94	Hsd17b7	15490	chr1	-2.7038484
95	Gm7694	665574	chr1	-3.044379
96	Fcgr2b	14130	chr1	-2.4627388
97	Pcp4l1	66425	chr1	-2.2269683
98	B4galt3	57370	chr1	-3.2200105
99	Nit1	27045	chr1	-3.9712765
100	Tstd1	226654	chr1	-7.286251
101	Kcnj10	16513	chr1	-2.6641502
102	Chml	12663	chr1	-2.3709118
103	Hmga2-ps1	15365	chr1	-2.2906425

104	Cdc42bpa	226751	chr1	-3.302927
105	Lefty1	13590	chr1	-3.7785118
106	Fbxo28	67948	chr1	-2.965663
107	Dusp10	63953	chr1	-2.1626937
108	C130074G19Rik	226777	chr1	-2.006698
109	Lyplal1	226791	chr1	-3.8432043
110	Kctd3	226823	chr1	-2.2074
111	Nenf	66208	chr1	-5.6967287
112	Slc30a1	22782	chr1	-3.030952
113	Fam171a1	269233	chr2	-2.1863549
114	Prpf18	67229	chr2	-2.3235166
115	Optn	71648	chr2	-19.931566
116	Camk1d	227541	chr2	-2.4483204
117	Echdc3	67856	chr2	-3.5605671
118	Kin	16588	chr2	-2.7895575
119	Prkcq	18761	chr2	-15.992312
120	Il15ra	16169	chr2	-2.999651
121	Trdmt1	13434	chr2	-2.0345457
122	Ptpla	30963	chr2	-4.3364573
123	Mrc1	17533	chr2	-2.4877539
124	Nsun6	74455	chr2	-4.1754117
125	Plxdc2	67448	chr2	-3.1332018
126	Armc3	70882	chr2	-2.3984673
127	Msrb2	76467	chr2	-2.4506824
128	Enkur	71233	chr2	-2.1127977
129	Mastl	67121	chr2	-5.270362
130	Hnmt	140483	chr2	-3.149634
131	Wdr85	67228	chr2	-2.2272456
132	Tprn	97031	chr2	-2.0496917
133	BC029214	227622	chr2	-3.204797
134	Phpt1	75454	chr2	-2.700251
135	Fcna	14133	chr2	-5.789515
136	Snapc4	227644	chr2	-4.2996407
137	Inpp5e	64436	chr2	-2.2171328
138	Cacfd1	381356	chr2	-2.7124507

139	Olfm1	56177	chr2	-3.140508
140	Gfi1b	14582	chr2	-6.22362
141	Ddx31	227674	chr2	-2.1383228
142	Ttf1	22130	chr2	-2.38116
143	Ntng2	171171	chr2	-3.2221305
144	Slc27a4	26569	chr2	-2.4866674
145	Dolk	227697	chr2	-3.7703707
146	Sh3glb2	227700	chr2	-3.1051497
147	Pomt1	99011	chr2	-2.0914538
148	Fam102a	98952	chr2	-9.318713
149	Eng	13805	chr2	-2.5047429
150	Stxbp1	20910	chr2	-2.6605604
151	Snora65	104367	chr2	-13.139156
152	Rabepk	227746	chr2	-6.3153787
153	Gsn	227753	chr2	-2.601872
154	Rabgap1	227800	chr2	-2.1177082
155	Dennd1a	227801	chr2	-2.6429536
156	Acvr2a	11480	chr2	-8.073645
157	Rnd3	74194	chr2	-5.048478
158	Fmnl2	71409	chr2	-3.2110217
159	Rprm	67874	chr2	-3.213822
160	Nr4a2	18227	chr2	-3.7877467
161	Galnt5	241391	chr2	-4.87715
162	Gm13546	100042926	chr2	-3.0788584
163	Acvr1	11477	chr2	-4.920293
164	Tanc1	66860	chr2	-2.5730073
165	Wdsub1	72137	chr2	-2.4577858
166	Cd302	66205	chr2	-4.8215857
167	Dpp4	13482	chr2	-2.6250246
168	Ifih1	71586	chr2	-4.6843133
169	Mettl8	228019	chr2	-2.0222354
170	Gpr155	68526	chr2	-3.2701375
171	Chrna1	11435	chr2	-2.316372
172	Fkbp7	14231	chr2	-4.3542876
173	Ube2e3	22193	chr2	-2.0694368

174	Pde1a	18573	chr2	-4.0213847
175	Nckap1	50884	chr2	-4.213773
176	Med19	381379	chr2	-3.1249397
177	Slc43a1	72401	chr2	-3.3602562
178	Prg3	53856	chr2	-9.476655
179	Tnks1bp1	228140	chr2	-3.1058688
180	Ptprj	19271	chr2	-2.1073778
181	Zfp408	381410	chr2	-2.0622942
182	Atg13	51897	chr2	-2.815703
183	D930015M05Rik	320507	chr2	-4.7644687
184	Chst1	76969	chr2	-4.16318
185	Trp53i11	277414	chr2	-2.7039905
186	B230118H07Rik	68170	chr2	-2.69785
187	Traf6	22034	chr2	-2.3445628
188	Commd9	76501	chr2	-3.660566
189	Cd59a	12509	chr2	-2.0914562
190	Mir1902	100316678	chr2	-42.767265
191	Prrg4	228413	chr2	-2.5767505
192	0610012H03Rik	74088	chr2	-3.9150443
193	Rcn1	19672	chr2	-2.2757435
194	Immp1l	66541	chr2	-3.6087875
195	Aven	74268	chr2	-4.581285
196	Spred1	114715	chr2	-2.001127
197	Bmf	171543	chr2	-3.7181442
198	A430105I19Rik	214239	chr2	-3.4823217
199	Chst14	72136	chr2	-4.777615
200	Jmjd7	433466	chr2	-2.3948686
201	Lcmt2	329504	chr2	-2.6753619
202	Zscan29	99334	chr2	-2.2691624
203	Ppip5k1	327655	chr2	-2.68083
204	Casc4	319996	chr2	-2.033281
205	Shf	435684	chr2	-2.3842378
206	Sqrdl	59010	chr2	-2.0814972
207	Shc4	271849	chr2	-4.5008373
208	Secisbp2l	70354	chr2	-2.0514581

209	Blvra	109778	chr2	-2.3607714
210	Dusp2	13537	chr2	-3.0903075
211	Adra2b	11552	chr2	-2.0365367
212	Zfp661	72180	chr2	-2.4727561
213	Zc3h8	57432	chr2	-2.4532208
214	Zc3h6	78751	chr2	-3.1770222
215	Ubox5	140629	chr2	-2.1514528
216	4930402H24Rik	228602	chr2	-3.5883148
217	Hspa12b	72630	chr2	-2.7886086
218	Smox	228608	chr2	-2.170761
219	Slc23a2	54338	chr2	-2.2226512
220	Gpcpd1	74182	chr2	-2.28022
221	Crls1	66586	chr2	-5.9239483
222	Plcb4	18798	chr2	-2.4761863
223	Zfp442	668923	chr2	-2.449279
224	Srxn1	76650	chr2	-4.588725
225	Trib3	228775	chr2	-2.7643676
226	Mcts2	66405	chr2	-4.1806636
227	2500004C02Rik	72326	chr2	-7.5710235
228	Map1lc3a	66734	chr2	-11.038223
229	Trp53inp2	68728	chr2	-6.013322
230	Acss2	60525	chr2	-2.8953154
231	Procr	19124	chr2	-2.177036
232	6430550D23Rik	320095	chr2	-6.502039
233	Romo1	67067	chr2	-3.4136233
234	Sla2	77799	chr2	-3.0849202
235	Dsn1	66934	chr2	-2.8803687
236	9830001H06Rik	320706	chr2	-3.3228586
237	Tgm2	21817	chr2	-10.3683605
238	Actr5	109275	chr2	-2.471316
239	Dhx35	71715	chr2	-3.086089
240	Plcg1	18803	chr2	-5.1148562
241	Ada	11486	chr2	-3.9083266
242	Sys1	66460	chr2	-3.7316315
243	Trp53rk	76367	chr2	-3.679069

244	Pard6b	58220	chr2	-5.349279
245	Kcng1	241794	chr2	-2.3799329
246	Nfatc2	18019	chr2	-2.6911807
247	Zfp64	22722	chr2	-2.3560708
248	Cstf1	67337	chr2	-2.6232295
249	Zbp1	58203	chr2	-3.255444
250	Ppp4r1l-ps	100043911	chr2	-2.3201742
251	Gm14393	664987	chr2	-2.8623202
252	Lsm14b	241846	chr2	-2.1694586
253	Psma7	26444	chr2	-2.4424615
254	Gtpbp5	52856	chr2	-4.2135572
255	Osbpl2	228983	chr2	-2.5219579
256	Rps21	66481	chr2	-3.3326702
257	Cables2	252966	chr2	-2.6952796
258	Gm14318	100036520	chr2	-2.7258782
259	B230312C02Rik	320485	chr2	-2.160833
260	Tcea2	21400	chr2	-3.7978187
261	Pcmtd2	245867	chr2	-2.109101
262	1700010I02Rik	75476	chr3	-4.394441
263	C030034L19Rik	320088	chr3	-3.2233684
264	Snx16	74718	chr3	-6.109657
265	Car2	12349	chr3	-2.7019553
266	Cpa3	12873	chr3	-5.570867
267	Pld1	18805	chr3	-3.9837546
268	Rpl22l1	68028	chr3	-3.6482537
269	Pik3ca	18706	chr3	-2.082452
270	Gnb4	14696	chr3	-4.293273
271	Ttc14	67120	chr3	-2.2190807
272	Bbs7	71492	chr3	-3.9131603
273	Spry1	24063	chr3	-4.6349125
274	Intu	380614	chr3	-2.9216301
275	Mfsd8	72175	chr3	-2.2210639
276	Gm2011	100039027	chr3	-5.293969
277	Pabpc4l	241989	chr3	-2.5103493
278	Spg20	229285	chr3	-3.3877

279	Wwtr1	97064	chr3	-2.3907168
280	Siah2	20439	chr3	-2.419965
281	Med12l	329650	chr3	-2.4762151
282	Gpr171	229323	chr3	-2.777303
283	P2ry14	140795	chr3	-8.249554
284	P2ry1	18441	chr3	-2.3777866
285	E130311K13Rik	329659	chr3	-4.858091
286	Trim59	66949	chr3	-2.0208526
287	Rapgef2	76089	chr3	-2.2056918
288	Ctso	229445	chr3	-5.5871763
289	Gucy1b3	54195	chr3	-2.5257814
290	Gucy1a3	60596	chr3	-2.4785008
291	Arfip1	99889	chr3	-2.0957863
292	Tigd4	403175	chr3	-2.525563
293	Tmem154	320782	chr3	-4.0533214
294	Fbxw7	50754	chr3	-2.7453012
295	Lrba	80877	chr3	-2.2696931
296	Cd1d2	12480	chr3	-3.7517946
297	Cd1d1	12479	chr3	-2.1148376
298	Cd5l	11801	chr3	-4.0310826
299	Pear1	73182	chr3	-2.2806451
300	Apoa1bp	246703	chr3	-5.5618944
301	Bglap	12096	chr3	-5.63964
302	Mex3a	72640	chr3	-3.1544456
303	Ubqln4	94232	chr3	-8.807744
304	Rit1	19769	chr3	-2.367116
305	Pklr	18770	chr3	-2.0206344
306	Gba	14466	chr3	-3.072815
307	Trim46	360213	chr3	-2.0759697
308	Cks1b	54124	chr3	-3.2865746
309	Hax1	23897	chr3	-2.6142082
310	1700094D03Rik	73545	chr3	-4.9871144
311	Rps27	57294	chr3	-2.2536397
312	Rab13	68328	chr3	-4.016027
313	Gatad2b	229542	chr3	-2.1042347

314	Snapin	20615	chr3	-2.2177677
315	S100a13	20196	chr3	-3.273576
316	S100a6	20200	chr3	-15.77112
317	Them4	75778	chr3	-4.2943845
318	Selenbp2	20342	chr3	-2.8170614
319	Mllt11	56772	chr3	-2.217492
320	Anxa9	71790	chr3	-12.438159
321	Bola1	69168	chr3	-2.1666272
322	Hist2h2be	319190	chr3	-6.19829
323	Hist2h4	97122	chr3	-14.045079
324	Txnip	56338	chr3	-3.5110464
325	Pias3	229615	chr3	-11.812569
326	Chd1l	68058	chr3	-2.47674
327	Fmo5	14263	chr3	-2.0614514
328	Igsf3	78908	chr3	-2.907274
329	Magi3	99470	chr3	-2.1067786
330	Lrig2	269473	chr3	-2.4483323
331	Dennd2d	72121	chr3	-6.246413
332	Dram2	67171	chr3	-2.8517613
333	Amigo1	229715	chr3	-3.497451
334	Taf13	99730	chr3	-2.8846173
335	Stxbp3a	20912	chr3	-3.4646025
336	S1pr1	13609	chr3	-2.7065904
337	Alg14	66789	chr3	-13.830999
338	Cnn3	71994	chr3	-3.9178846
339	Fnbp1l	214459	chr3	-2.6681335
340	Pde5a	242202	chr3	-2.8738284
341	Egf	13645	chr3	-3.164616
342	Casp6	12368	chr3	-2.3736372
343	Ints12	71793	chr3	-2.617135
344	Dnajb14	70604	chr3	-7.0767164
345	Gbp2	14469	chr3	-5.131669
346	Gng5	14707	chr3	-2.0345457
347	Lphn2	99633	chr3	-2.0849705
348	Eltd1	170757	chr3	-2.5810175

349	Ifi44	99899	chr3	-2.9173007
350	Dnajb4	67035	chr3	-3.5161126
351	5730460C07Rik	70594	chr3	-3.9442627
352	Tyw3	209584	chr3	-2.199724
353	Fpgt	75540	chr3	-3.425689
354	Lrrc40	67144	chr3	-2.4929628
355	Chchd7	66433	chr4	-4.747276
356	Fam110b	242297	chr4	-3.2069
357	Pdp1	381511	chr4	-4.184898
358	Fam92a	68099	chr4	-2.6421483
359	Tmem55a	72519	chr4	-4.9955926
360	Gm11837	100038514	chr4	-2.6184042
361	Ccnc	51813	chr4	-4.1497154
362	Casp8ap2	26885	chr4	-2.612406
363	Lyrm2	108755	chr4	-29.39963
364	Ankrd6	140577	chr4	-3.8963506
365	Pnrc1	108767	chr4	-2.5644124
366	Slc35a1	24060	chr4	-2.4401784
367	Ddx58	230073	chr4	-2.9064934
368	2010003O02Rik	100504309	chr4	-11.728562
369	Chmp5	76959	chr4	-2.9749167
370	Aqp3	11828	chr4	-3.358463
371	1700022I11Rik	67317	chr4	-3.5680382
372	Pigo	56703	chr4	-6.0461473
373	Tesk1	21754	chr4	-2.4346046
374	Cd72	12517	chr4	-3.3975687
375	Car9	230099	chr4	-2.5448909
376	Tpm2	22004	chr4	-4.225595
377	Hint2	68917	chr4	-7.9393916
378	Reck	53614	chr4	-2.0728276
379	Melk	17279	chr4	-2.818972
380	Shb	230126	chr4	-2.4224691
381	Tdrd7	100121	chr4	-5.869474
382	Trim14	74735	chr4	-2.9758818
383	Alg2	56737	chr4	-2.3626373

384	Acnat1	230161	chr4	-2.6788042
385	Abca1	11303	chr4	-3.7795608
386	Slc44a1	100434	chr4	-6.638996
387	Epb4.1l4b	54357	chr4	-2.2652538
388	D630039A03Rik	242484	chr4	-4.7687554
389	Slc31a2	20530	chr4	-2.0997562
390	Hdhd3	72748	chr4	-8.12358
391	Pole3	59001	chr4	-2.1067781
392	Trim32	69807	chr4	-2.2633848
393	3110001D03Rik	66928	chr4	-3.0583405
394	Ttc39b	69863	chr4	-3.3822722
395	Cntln	338349	chr4	-2.0481546
396	Mllt3	70122	chr4	-2.179675
397	Tek	21687	chr4	-5.083027
398	Jun	16476	chr4	-2.0457244
399	Fggy	75578	chr4	-4.522552
400	Cyp2j9	74519	chr4	-13.328884
401	Nfia	18027	chr4	-2.8514073
402	Tm2d1	94043	chr4	-33.708138
403	Inadl	12695	chr4	-7.746605
404	Wdr78	242584	chr4	-4.329721
405	Ttc4	72354	chr4	-2.9210322
406	Ssbp3	72475	chr4	-2.6811523
407	Hspb11	72938	chr4	-5.0066957
408	Cpt2	12896	chr4	-2.928039
409	Echdc2	52430	chr4	-2.0941892
410	Txndc12	66073	chr4	-2.1008003
411	9130206I24Rik	100040736	chr4	-3.2629902
412	Mob3c	100465	chr4	-2.1768801
413	Faah	14073	chr4	-6.7050734
414	Gpbp1l1	77110	chr4	-2.0420485
415	Mpl	17480	chr4	-5.1456456
416	Slc2a1	20525	chr4	-2.2278736
417	Ermap	27028	chr4	-2.2436743
418	AU022252	230696	chr4	-2.3260498

419	AA415398	433752	chr4	-4.655981
420	Zfp69	381549	chr4	-2.7291658
421	Smap2	69780	chr4	-2.024183
422	Inpp5b	16330	chr4	-2.3285823
423	9930104L06Rik	194268	chr4	-2.4741066
424	Grik3	14807	chr4	-2.026375
425	AU040320	100317	chr4	-2.1313665
426	Gm12942	100039968	chr4	-3.7776713
427	Gjb3	14620	chr4	-2.33958
428	Iqcc	230767	chr4	-3.2865746
429	Ccdc28b	66264	chr4	-2.821567
430	1700003M07Rik	72225	chr4	-2.1023612
431	Col16a1	107581	chr4	-2.8586361
432	Snord85	100217460	chr4	-45.40976
433	Xkr8	381560	chr4	-2.1171043
434	Stx12	100226	chr4	-2.1278462
435	Ahdc1	230793	chr4	-2.0658467
436	Mir5122	100628620	chr4	-7.688498
437	Gpn2	100210	chr4	-3.0277712
438	Cd52	23833	chr4	-3.1235216
439	Sepn1	74777	chr4	-2.3429387
440	Rhd	19746	chr4	-2.3243957
441	Id3	15903	chr4	-10.462626
442	C1qc	12262	chr4	-5.7502203
443	C1qa	12259	chr4	-9.757305
444	Pink1	68943	chr4	-2.757158
445	Padi4	18602	chr4	-2.0723372
446	Epha2	13836	chr4	-2.4345002
447	Rsc1a1	69994	chr4	-2.097779
448	Dnajc16	214063	chr4	-2.6018715
449	Pramef8	242736	chr4	-2.5230267
450	Fv1	14349	chr4	-5.60403
451	Mad2l2	71890	chr4	-2.1910484
452	Casz1	69743	chr4	-3.4501326
453	Nmnat1	66454	chr4	-3.2865748

454	Ctnnbip1	67087	chr4	-2.4344997
455	Slc25a33	70556	chr4	-2.2554915
456	Thap3	69876	chr4	-2.3806596
457	Tnfrsf25	85030	chr4	-3.5369418
458	Dffb	13368	chr4	-2.1823752
459	Tprgl	67808	chr4	-2.6452913
460	5930403L14Rik	320939	chr4	-3.6791196
461	Tnfrsf14	230979	chr4	-11.165601
462	Pex10	668173	chr4	-2.7127283
463	2610002J02Rik	67513	chr4	-2.7388144
464	2010015L04Rik	544678	chr4	-4.046909
465	B930041F14Rik	230991	chr4	-7.5397887
466	Aurkaip1	66077	chr4	-2.070992
467	Pusl1	433813	chr4	-2.5969298
468	Fam132a	67389	chr4	-3.551991
469	Tnfrsf18	21936	chr4	-6.9715266
470	Isg15	100038882	chr4	-4.4112463
471	AW011738	100382	chr4	-4.0597186
472	Plekhn1	231002	chr4	-4.20774
473	Gatad1	67210	chr5	-2.249996
474	Abcb1a	18671	chr5	-5.79412
475	Abcb4	18670	chr5	-2.3866796
476	Hgf	15234	chr5	-2.008955
477	Pmpcb	73078	chr5	-2.4105158
478	Gm15421	100042049	chr5	-3.7058492
479	Tomm7	66169	chr5	-2.2477133
480	Mir671	735264	chr5	-13.964822
481	2900005J15Rik	67261	chr5	-2.1091847
482	Galnt11	231050	chr5	-3.024601
483	Insig1	231070	chr5	-2.3051665
484	Slc5a6	330064	chr5	-4.324327
485	Krtcap3	69815	chr5	-3.6367137
486	Mrpl33	66845	chr5	-3.7247858
487	Fam53a	74504	chr5	-2.8953154
488	Tmem129	68366	chr5	-2.4394877

489	Hgfac	54426	chr5	-3.2538102
490	Wfs1	22393	chr5	-7.1639924
491	Jakmip1	76071	chr5	-2.6984525
492	Clnk	27278	chr5	-2.436985
493	Pi4k2b	67073	chr5	-2.3621228
494	Tbc1d19	67249	chr5	-4.1082144
495	Fam114a1	68303	chr5	-2.4421906
496	Lias	79464	chr5	-3.4991384
497	Commd8	27784	chr5	-12.809934
498	Ociad2	433904	chr5	-2.355173
499	Usp46	69727	chr5	-2.2563326
500	C530008M17Rik	320827	chr5	-3.2523384
501	Ythdc1	231386	chr5	-2.1893466
502	Igj	16069	chr5	-4.1376276
503	Mob1b	68473	chr5	-3.7471004
504	Cox18	231430	chr5	-2.6380327
505	Gm9958	791294	chr5	-6.1587486
506	Rassf6	73246	chr5	-4.3934274
507	Cxcl5	20311	chr5	-8.400457
508	Pf4	56744	chr5	-17.289778
509	Mthfd2l	665563	chr5	-2.3475528
510	Gm19619	100503265	chr5	-2.9435654
511	Thap6	381650	chr5	-2.1421578
512	Cxcl10	15945	chr5	-9.597642
513	Ccdc158	320696	chr5	-2.2583375
514	Mir703	735265	chr5	-9.311961
515	Mrps18c	68735	chr5	-2.8775964
516	BC005561	100042165	chr5	-2.3553784
517	Abcg3	27405	chr5	-3.5441375
518	Gbp6	100702	chr5	-2.861253
519	Lrrc8c	100604	chr5	-2.4539752
520	Brdt	114642	chr5	-2.9180224
521	A830010M20Rik	231570	chr5	-2.4308217
522	Evi5	14020	chr5	-2.387162
523	Ccdc18	73254	chr5	-3.6422622

524	Tmem175	72392	chr5	-4.945895
525	Zfp932	69504	chr5	-11.658565
526	Mn1	433938	chr5	-3.9590304
527	Tpst2	22022	chr5	-3.0276325
528	2900026A02Rik	243219	chr5	-3.9104517
529	Ficd	231630	chr5	-2.5132627
530	Ssh1	231637	chr5	-4.804532
531	4930515G01Rik	67642	chr5	-3.281901
532	Oasl1	231655	chr5	-4.2978277
533	Msi1	17690	chr5	-2.268945
534	Ccdc64	75665	chr5	-4.7478895
535	Fbxo21	231670	chr5	-2.0206347
536	Sdsl	257635	chr5	-2.066558
537	Rasal1	19415	chr5	-3.9613416
538	Oas3	246727	chr5	-2.1237228
539	Vps29	56433	chr5	-13.033837
540	P2rx7	18439	chr5	-3.1065788
541	P2rx4	18438	chr5	-2.7544594
542	A930024E05Rik	109202	chr5	-2.273343
543	Rhof	23912	chr5	-6.0253973
544	Diablo	66593	chr5	-2.138882
545	Ccdc62	208908	chr5	-5.186934
546	Rilpl1	75695	chr5	-2.4340641
547	Aacs	78894	chr5	-2.817064
548	Stx2	13852	chr5	-8.689206
549	Zfp11	22648	chr5	-2.158954
550	Mrps17	66258	chr5	-2.4824197
551	Sumf2	67902	chr5	-2.2184317
552	Tpst1	22021	chr5	-3.3214648
553	Clip2	269713	chr5	-4.160221
554	Lat2	56743	chr5	-2.0645154
555	Tbl2	27368	chr5	-2.0124571
556	Por	18984	chr5	-2.1063135
557	Orai2	269717	chr5	-3.0280793
558	Prkrip1	66801	chr5	-2.25952

559	Mir702	735283	chr5	-2.7540333
560	Pop7	74097	chr5	-12.589498
561	Sap25	751865	chr5	-4.242283
562	Ppp1r35	69871	chr5	-2.4831893
563	Zcwpw1	381678	chr5	-3.0663726
564	Gjc3	118446	chr5	-2.0005584
565	Zscan21	22697	chr5	-2.5618422
566	Nxpe5	381680	chr5	-2.1910493
567	Zfp157	72154	chr5	-2.3143413
568	Lfng	16848	chr5	-2.6241636
569	Iqce	74239	chr5	-3.7110898
570	Rbak	57782	chr5	-4.281768
571	Zfp12	231866	chr5	-2.7131739
572	Daglb	231871	chr5	-5.0681643
573	Tecpr1	70381	chr5	-3.3809347
574	Tmem130	243339	chr5	-2.157012
575	Atp5j2	57423	chr5	-4.1424522
576	Zkscan14	67235	chr5	-7.5992312
577	Zkscan5	22757	chr5	-2.2677603
578	Rnf6	74132	chr5	-3.4668236
579	Flt3	14255	chr5	-2.0536005
580	Ubl3	24109	chr5	-2.312477
581	Fry	320365	chr5	-3.004686
582	N4bp2l2	381695	chr5	-2.995059
583	Rfc3	69263	chr5	-3.262768
584	Sgce	20392	chr6	-9.654312
585	Glcci1	170772	chr6	-2.235224
586	Ica1	15893	chr6	-2.019874
587	Mdfic	16543	chr6	-2.5402484
588	Tes	21753	chr6	-2.0322387
589	Cadps2	320405	chr6	-5.661729
590	Wasl	73178	chr6	-2.0754108
591	Ccdc136	232664	chr6	-3.752221
592	Atp6v1f	66144	chr6	-2.3375452
593	Irf5	27056	chr6	-2.0246403

594	Tspan33	232670	chr6	-2.7886076
595	1700023L04Rik	76419	chr6	-4.1346
596	Ube2h	22214	chr6	-2.020551
597	2210408F21Rik	73652	chr6	-2.4414964
598	Tmem140	68487	chr6	-5.9569173
599	Wdr91	101240	chr6	-3.7070062
600	Cnot4	53621	chr6	-2.6449099
601	D630045J12Rik	330286	chr6	-3.0428681
602	Gstk1	76263	chr6	-20.18142
603	Arhgef5	54324	chr6	-3.568281
604	Tpk1	29807	chr6	-5.0364933
605	Rn4.5s	19799	chr6	-3.8202605
606	Zfp398	272347	chr6	-2.7315085
607	Zfp282	101095	chr6	-2.9747097
608	Zfp783	232785	chr6	-3.7497807
609	Zfp467	68910	chr6	-3.2314718
610	Zfp775	243372	chr6	-3.9264307
611	AI854703	243373	chr6	-2.8453374
612	Gimap8	243374	chr6	-2.9156933
613	Gimap9	317758	chr6	-4.7842536
614	Gimap4	107526	chr6	-9.397323
615	Gimap6	231931	chr6	-4.6650634
616	Gimap7	231932	chr6	-8.627597
617	Gimap1	16205	chr6	-2.8622305
618	Gimap5	317757	chr6	-27.1678
619	Tmem176b	65963	chr6	-2.6569216
620	Tmem176a	66058	chr6	-3.5554762
621	Hoxa5	15402	chr6	-2.4998295
622	Mira	100736249	chr6	-2.8829598
623	Mir196b	723820	chr6	-12.730721
624	Chn2	69993	chr6	-4.479902
625	Ggct	110175	chr6	-9.855487
626	Ppm1k	243382	chr6	-3.4402895
627	Abcg2	26357	chr6	-4.993067
628	Tigd2	68140	chr6	-5.228641

629	Mmrn1	70945	chr6	-6.528128
630	Hpgds	54486	chr6	-3.2469194
631	Tacstd2	56753	chr6	-3.1153984
632	Thnsl2	232078	chr6	-5.4776263
633	Gm20362	100504701	chr6	-5.4073553
634	Pcgf1	69837	chr6	-12.079402
635	Bola3	78653	chr6	-2.9735675
636	Stambp	70527	chr6	-6.9791737
637	Nagk	56174	chr6	-2.390236
638	Paip2b	232164	chr6	-4.1906476
639	Exoc6b	75914	chr6	-2.364025
640	Rab11fip5	52055	chr6	-3.9901228
641	Tprkb	69786	chr6	-2.813086
642	C87436	232196	chr6	-2.0272384
643	2310040G24Rik	381792	chr6	-8.829372
644	Podxl2	319655	chr6	-2.1505826
645	Tpra1	24100	chr6	-3.8472133
646	Chst13	71797	chr6	-8.407652
647	Hdac11	232232	chr6	-3.8841333
648	1810044D09Rik	69798	chr6	-8.850134
649	Fgd5	232237	chr6	-4.9381123
650	Mitf	17342	chr6	-4.644153
651	Setmar	74729	chr6	-4.4182487
652	Srgap3	259302	chr6	-3.780834
653	Gt(ROSA)26Sor	14910	chr6	-2.4365983
654	Il17re	57890	chr6	-3.8047945
655	Ift122	81896	chr6	-3.1727307
656	Tmcc1	330401	chr6	-2.5841668
657	Cxcl12	20315	chr6	-2.2933433
658	Ankrd26	232339	chr6	-2.0033312
659	Dcp1b	319618	chr6	-2.3553774
660	Slc2a3	20527	chr6	-2.8149104
661	Foxj2	60611	chr6	-2.6629677
662	Pex5	19305	chr6	-2.0710342
663	C1ra	50909	chr6	-3.6676266

664	Grcc10	14790	chr6	-2.2926958
665	Lag3	16768	chr6	-4.8188334
666	Ptms	69202	chr6	-3.7005467
667	Acrbp	54137	chr6	-2.251912
668	Tapbpl	213233	chr6	-2.3915825
669	Cd27	21940	chr6	-2.1305432
670	Cd9	12527	chr6	-2.561843
671	Vwf	22371	chr6	-2.6336193
672	D6Wsu163e	28040	chr6	-2.0801098
673	Klrb1c	17059	chr6	-3.6397674
674	Clec2i	93675	chr6	-21.198399
675	Clec2d	93694	chr6	-4.9923663
676	Clec1a	243653	chr6	-3.8287885
677	Tas2r123	353167	chr6	-3.811593
678	Cdkn1b	12576	chr6	-2.062164
679	1190002F15Rik	381822	chr6	-2.3561826
680	Atf7ip	54343	chr6	-2.011192
681	Plbd1	66857	chr6	-2.2779498
682	Hist4h4	320332	chr6	-2.037244
683	Art4	109978	chr6	-3.8343363
684	Pyroxd1	232491	chr6	-2.3451285
685	Ldhb	16832	chr6	-5.0050445
686	Gm15706	100039495	chr6	-7.5025454
687	Rassf8	71323	chr6	-5.9398556
688	Itpr2	16439	chr6	-3.446402
689	Mrps35	232536	chr6	-4.1499524
690	Dennd5b	320560	chr6	-2.594038
691	Mettl20	320204	chr6	-2.7271593
692	2810474O19Rik	67246	chr6	-2.1850657
693	Ndufa3	66091	chr7	-2.7577012
694	Lilrb3	18733	chr7	-5.542247
695	Eps8l1	67425	chr7	-3.6993654
696	Tnni3	21954	chr7	-3.0674694
697	Tmem86b	68255	chr7	-7.8953123
698	Isoc2b	67441	chr7	-2.5334013

699	Zfp628	232816	chr7	-6.181891
700	Vmn2r55	100042499	chr7	-5.3335385
701	Zfp329	67230	chr7	-2.8750565
702	Zfp324	243834	chr7	-2.7388117
703	Zbtb45	232879	chr7	-3.2865741
704	6330408A02Rik	321008	chr7	-4.731427
705	Cabp5	29865	chr7	-2.4689603
706	Crx	12951	chr7	-4.718174
707	Prr24	66300	chr7	-2.4729204
708	Fkrp	243853	chr7	-2.4101546
709	Prkd2	101540	chr7	-2.9087057
710	Ccdc8	434130	chr7	-2.2863126
711	Pglyrp1	21946	chr7	-5.454687
712	Ccdc61	232933	chr7	-3.1262536
713	Six5	20475	chr7	-7.019473
714	A930016O22Rik	100503495	chr7	-11.451256
715	Pvr	52118	chr7	-9.680613
716	Zfp235	56525	chr7	-3.4169939
717	Zfp109	56869	chr7	-5.6917133
718	Cd79a	12518	chr7	-5.303898
719	Grik5	14809	chr7	-2.7964709
720	Zfp526	210172	chr7	-2.0399425
721	4732471J01Rik	654804	chr7	-2.0243704
722	Bckdha	12039	chr7	-2.475074
723	B9d2	232987	chr7	-2.9962204
724	Axl	26362	chr7	-3.8311698
725	Itpkc	233011	chr7	-3.1222456
726	Sertad1	55942	chr7	-2.095786
727	Hipk4	233020	chr7	-2.2676928
728	Pld3	18807	chr7	-5.085307
729	Map3k10	269881	chr7	-4.079695
730	Zfp60	22718	chr7	-3.0945745
731	Gm4636	100043772	chr7	-2.6990986
732	Dyrk1b	13549	chr7	-8.309992
733	Zfp36	22695	chr7	-2.1225896

734	Sars2	71984	chr7	-3.1344218
735	Ech1	51798	chr7	-3.1136813
736	Spred3	101809	chr7	-3.2676497
737	Ggn	243897	chr7	-2.8003337
738	Ppp1r14a	68458	chr7	-6.2591863
739	Sipa1l3	74206	chr7	-2.925408
740	Zfp84	74352	chr7	-2.1636622
741	Zfp790	233056	chr7	-3.0755484
742	Zfp940	233057	chr7	-2.1910496
743	Zfp74	72723	chr7	-4.706872
744	Zfp82	330502	chr7	-2.4179375
745	Clip3	76686	chr7	-2.3105433
746	Alkbh6	233065	chr7	-2.1910496
747	Hcst	23900	chr7	-5.7502203
748	Arhgap33	233071	chr7	-3.3257
749	BC053749	333193	chr7	-4.24405
750	U2af1l4	233073	chr7	-4.1579123
751	Sbsn	282619	chr7	-10.861445
752	Pdcd2l	68079	chr7	-3.019373
753	4931406P16Rik	233103	chr7	-3.3661761
754	C230052I12Rik	101831	chr7	-12.598535
755	Pop4	66161	chr7	-4.4259195
756	Gm5595	434179	chr7	-3.1076272
757	Zfp715	69930	chr7	-3.3039634
758	Zfp658	210104	chr7	-2.1400948
759	Klk8	259277	chr7	-5.1760693
760	Snord88a	100217433	chr7	-9.472266
761	Nr1h2	22260	chr7	-6.061905
762	Vrk3	101568	chr7	-2.1208239
763	Bcl2l12	75736	chr7	-2.9344418
764	Irf3	54131	chr7	-2.7787435
765	Prrg2	65116	chr7	-6.461816
766	Fcgrt	14132	chr7	-3.5995815
767	Tead2	21677	chr7	-3.6975412
768	Dbp	13170	chr7	-2.2137322

769	Cyth2	19158	chr7	-2.1832366
770	Tsg101	22088	chr7	-2.5385003
771	Spty2d1	101685	chr7	-2.618045
772	Tmem86a	67893	chr7	-4.958691
773	Gas2	14453	chr7	-11.069914
774	Nipa1	233280	chr7	-2.2348707
775	Ndn	17984	chr7	-4.618288
776	Tm2d3	68634	chr7	-2.7289183
777	Lins	72635	chr7	-3.0309515
778	Fam174b	100038347	chr7	-2.6890156
779	Klhl25	207952	chr7	-4.4493003
780	Mfge8	17304	chr7	-2.2783031
781	Polg	18975	chr7	-2.629307
782	Alpk3	116904	chr7	-2.6092227
783	2900076A07Rik	100504421	chr7	-2.6170876
784	Zfand6	65098	chr7	-2.2941625
785	Rab38	72433	chr7	-2.779387
786	AI314278	101521	chr7	-3.1396673
787	Snord15a	449630	chr7	-56.03483
788	Ppme1	72590	chr7	-2.5715904
789	Atg16l2	73683	chr7	-2.922569
790	Stard10	56018	chr7	-15.813661
791	Rnf121	75212	chr7	-2.0363877
792	Trim21	20821	chr7	-2.611653
793	Trim68	101700	chr7	-2.0056548
794	Hbb-b1	15129	chr7	-2.9494905
795	Beta-s	100503605	chr7	-3.1354668
796	Trim30b	244183	chr7	-2.398865
797	Trim30a	20128	chr7	-2.5542755
798	Trim30d	209387	chr7	-3.1631339
799	Apbb1	11785	chr7	-2.2776368
800	Tpp1	12751	chr7	-2.3189626
801	Gm4759	209380	chr7	-3.09395
802	Snora3	100302499	chr7	-85.43796
803	Dennd5a	19347	chr7	-2.1451104

804	Sbf2	319934	chr7	-2.5656438
805	Ampd3	11717	chr7	-4.719809
806	Sox6	20679	chr7	-5.6675525
807	Gprc5b	64297	chr7	-3.6517494
808	2610020H08Rik	434234	chr7	-2.1371715
809	Tmem159	233806	chr7	-4.161295
810	Palb2	233826	chr7	-2.0897777
811	Dctn5	59288	chr7	-2.328851
812	Gsg1l	269994	chr7	-6.0357637
813	Sbk1	104175	chr7	-2.1266067
814	Ccdc101	75565	chr7	-2.393626
815	Ino80e	233875	chr7	-19.532835
816	Qprt	67375	chr7	-2.776631
817	Gm4532	100043580	chr7	-11.076568
818	Zfp553	233887	chr7	-2.670767
819	9130019O22Rik	78921	chr7	-3.218126
820	Zfp688	69234	chr7	-3.6561444
821	Snora30	100217442	chr7	-6.710089
822	Zfp629	320683	chr7	-2.1354387
823	Ctf1	13019	chr7	-5.5772176
824	Cox6a2	12862	chr7	-11.570247
825	Bag3	29810	chr7	-2.4609613
826	Tacc2	57752	chr7	-2.9109852
827	Pstk	214580	chr7	-3.3896968
828	Bub3	12237	chr7	-3.1698387
829	Dhx32	101437	chr7	-2.1034071
830	9430038I01Rik	77252	chr7	-3.6494734
831	Mapk1ip1	69546	chr7	-2.4154272
832	Lrrc27	76612	chr7	-2.8125389
833	Mtg1	212508	chr7	-3.4691617
834	Sirt3	64384	chr7	-2.2570572
835	Athl1	212974	chr7	-2.2916589
836	Ifitm1	68713	chr7	-5.927068
837	Ifitm3	66141	chr7	-2.713641
838	Pddc1	213350	chr7	-3.919582

839	Cd151	12476	chr7	-2.1061249
840	Tspan4	64540	chr7	-2.05359
841	Mrpl23	19935	chr7	-2.8971496
842	Tspan32	27027	chr7	-2.574783
843	Cdkn1c	12577	chr7	-2.6175053
844	Tnfrsf22	79202	chr7	-6.1015525
845	Dhcr7	13360	chr7	-2.873169
846	Cttn	13043	chr7	-2.967548
847	Fadd	14082	chr7	-2.085295
848	Fgf3	14174	chr7	-10.479464
849	Arhgef18	102098	chr8	-2.2143283
850	Zfp358	140482	chr8	-4.466367
851	Camsap3	69697	chr8	-3.5497198
852	Snapc2	102209	chr8	-2.1333904
853	Lig4	319583	chr8	-3.3999043
854	Col4a1	12826	chr8	-5.086368
855	Col4a2	12827	chr8	-2.373634
856	Cars2	71941	chr8	-2.1910505
857	Ing1	26356	chr8	-2.0513651
858	Cdc16	69957	chr8	-2.9202063
859	Arhgef10	234094	chr8	-2.9643996
860	Kbtbd11	74901	chr8	-2.246047
861	Nek5	330721	chr8	-2.4397774
862	Nek3	23954	chr8	-2.956455
863	Polb	18970	chr8	-2.8757527
864	A930013F10Rik	68074	chr8	-2.1910496
865	Ap3m2	64933	chr8	-2.413578
866	Ank1	11733	chr8	-2.3347256
867	Letm2	270035	chr8	-2.4384267
868	Purg	75029	chr8	-4.3384576
869	Ubxn8	108159	chr8	-2.8089154
870	Rbpms	19663	chr8	-4.755574
871	Leprotl1	68192	chr8	-2.2790833
872	Dusp4	319520	chr8	-3.160387
873	Vps37a	52348	chr8	-2.4269154

874	4933411K20Rik	66756	chr8	-2.3595922
875	Rwdd4a	192174	chr8	-2.3986228
876	Aga	11593	chr8	-2.013832
877	Nek1	18004	chr8	-2.254741
878	Sh3rf1	59009	chr8	-2.4150705
879	Tmem192	73067	chr8	-2.1472287
880	Csgalnact1	234356	chr8	-9.372824
881	Slc18a1	110877	chr8	-2.5676363
882	Lzts1	211134	chr8	-3.6929195
883	Zfp930	234358	chr8	-4.5099106
884	D130040H23Rik	211135	chr8	-4.749342
885	Gm10033	378466	chr8	-2.6157746
886	Slc25a42	73095	chr8	-3.3609858
887	Crtc1	382056	chr8	-2.8911853
888	Klhl26	234378	chr8	-2.4649305
889	Pgpep1	66522	chr8	-2.0393615
890	Jund	16478	chr8	-2.009482
891	Pik3r2	18709	chr8	-3.1481702
892	Ccdc124	234388	chr8	-2.1412532
893	Use1	67023	chr8	-2.3889968
894	Nr2f6	13864	chr8	-3.3550446
895	Gtpbp3	70359	chr8	-4.4049525
896	Slc27a1	26457	chr8	-2.7987733
897	Insl3	16336	chr8	-3.810851
898	Zfp882	382019	chr8	-3.1794105
899	1700030K09Rik	72254	chr8	-20.058249
900	Tmem38a	74166	chr8	-2.6117415
901	F2rl3	14065	chr8	-2.6659474
902	Hmox1	15368	chr8	-3.3494327
903	0610038B21Rik	70345	chr8	-3.561521
904	Zfp827	622675	chr8	-2.0138364
905	Mmaa	109136	chr8	-3.7822416
906	Rnf150	330812	chr8	-2.6730018
907	Il27ra	50931	chr8	-6.573148
908	Zswim4	212168	chr8	-4.944667

909	Ier2	15936	chr8	-2.6256402
910	Syce2	71846	chr8	-2.1799831
911	Klf1	16596	chr8	-4.71563
912	Dhps	330817	chr8	-3.1010847
913	BC056474	414077	chr8	-2.1133528
914	4921524J17Rik	66714	chr8	-3.307642
915	Gm10638	666945	chr8	-6.6030436
916	Nkd1	93960	chr8	-2.1484342
917	Snx20	71607	chr8	-3.14368
918	Mmp2	17390	chr8	-7.7791543
919	Ccdc102a	234582	chr8	-2.4530222
920	Gpr56	14766	chr8	-2.213626
921	Katnb1	74187	chr8	-3.4235168
922	Setd6	66083	chr8	-4.3344674
923	Fam96b	68523	chr8	-4.789011
924	Tradd	71609	chr8	-2.122579
925	4931428F04Rik	74356	chr8	-2.3073382
926	Acd	497652	chr8	-6.7288513
927	Pard6a	56513	chr8	-3.177022
928	Dpep3	71854	chr8	-8.839126
929	Dus2l	66369	chr8	-3.8919961
930	Pdf	68023	chr8	-2.6055725
931	Nqo1	18104	chr8	-5.3993716
932	Txnl4b	234723	chr8	-11.154435
933	Fuk	234730	chr8	-3.0007849
934	Gabarapl2	93739	chr8	-4.255343
935	Terf2ip	57321	chr8	-3.617151
936	Nudt7	67528	chr8	-2.3622258
937	Bcmo1	63857	chr8	-2.1987479
938	Sdr42e1	74032	chr8	-2.8118472
939	Mphosph6	68533	chr8	-3.9954429
940	Hsbp1	68196	chr8	-2.2647855
941	4632415K11Rik	74347	chr8	-2.539625
942	1190005I06Rik	68918	chr8	-3.995385
943	Banp	53325	chr8	-2.8672044

944	Cbfa2t3	12398	chr8	-2.4895358
945	Rab4a	19341	chr8	-3.1648493
946	Ttc13	234875	chr8	-2.4557247
947	Slc35f3	210027	chr8	-2.0372684
948	Alkbh8	67667	chr9	-2.2636642
949	Kbtbd3	69149	chr9	-2.7996745
950	Casp4	12363	chr9	-3.856848
951	Casp12	12364	chr9	-2.4335964
952	Birc2	11797	chr9	-4.9655848
953	Trpc6	22068	chr9	-4.259292
954	Cep57	74360	chr9	-2.458561
955	Sesn3	75747	chr9	-4.405917
956	Zfp317	244713	chr9	-2.457193
957	Ubl5	66177	chr9	-2.4010816
958	Ap1m2	11768	chr9	-2.6997585
959	Kank2	235041	chr9	-2.5164285
960	Pigyl	66268	chr9	-2.2634733
961	Rp9	55934	chr9	-2.2650712
962	Ets1	23871	chr9	-4.3099384
963	Tirap	117149	chr9	-2.2129886
964	Pus3	67049	chr9	-2.130493
965	Gm5615	434396	chr9	-2.091506
966	Tmem218	66279	chr9	-18.592361
967	Esam	69524	chr9	-7.4658003
968	Vsig2	57276	chr9	-4.678811
969	Siae	22619	chr9	-11.853837
970	Zfp202	80902	chr9	-3.8776383
971	Gramd1b	235283	chr9	-2.3352265
972	Arhgef12	69632	chr9	-3.1377995
973	Tmem136	235300	chr9	-2.908603
974	Pvrl1	58235	chr9	-2.4723337
975	Thy1	21838	chr9	-9.127439
976	Usp2	53376	chr9	-2.3179178
977	Ccdc84	382073	chr9	-7.149841
978	Sik3	70661	chr9	-3.37724

979	Zfp259	22687	chr9	-2.7231627
980	Htr3b	57014	chr9	-2.432694
981	Pts	19286	chr9	-2.2133589
982	Il18	16173	chr9	-4.289426
983	Alg9	102580	chr9	-2.4945197
984	Layn	244864	chr9	-3.4691606
985	Pou2af1	18985	chr9	-2.7045767
986	Fdx1	14148	chr9	-9.129373
987	Dnaja4	58233	chr9	-2.6674902
988	Wdr61	66317	chr9	-2.0949502
989	Scaper	244891	chr9	-4.0293026
990	Man2c1	73744	chr9	-2.8301058
991	Cyp11a1	13070	chr9	-3.9674578
992	Stoml1	69106	chr9	-2.5946639
993	Cd276	102657	chr9	-2.8236563
994	Adpgk	72141	chr9	-2.404762
995	Bbs4	102774	chr9	-2.6149707
996	Celf6	76183	chr9	-2.3478873
997	Parp6	67287	chr9	-6.6848106
998	Lrrc49	102747	chr9	-2.0577736
999	Uaca	72565	chr9	-3.5393894
1000	Coro2b	235431	chr9	-3.5743754
1001	Uchl4	93841	chr9	-9.169672
1002	Parp16	214424	chr9	-2.2640843
1003	Mtfmt	69606	chr9	-2.421682
1004	Usp3	235441	chr9	-4.4397616
1005	Aph1c	68318	chr9	-2.8352976
1006	Fam63b	235461	chr9	-4.903417
1007	Aqp9	64008	chr9	-6.604599
1008	2310009A05Rik	66364	chr9	-17.484413
1009	Myo5c	208943	chr9	-2.4046524
1010	4933433G15Rik	71274	chr9	-3.899976
1011	Lysmd2	70082	chr9	-5.2135334
1012	Lrrc1	214345	chr9	-4.354758
1013	Gclc	14629	chr9	-2.1242874

1014	Phip	83946	chr9	-2.180646
1015	Ube2cbp	70348	chr9	-2.7807415
1016	Snx14	244962	chr9	-2.2544272
1017	Zfp949	71640	chr9	-3.330396
1018	Gm2382	100039707	chr9	-11.383938
1019	9330159M07Rik	319673	chr9	-3.678916
1020	Bcl2a1b	12045	chr9	-2.6188645
1021	Mthfs	107885	chr9	-37.039516
1022	Plscr4	235527	chr9	-2.895316
1023	1190002N15Rik	68861	chr9	-2.6292593
1024	Spsb4	211949	chr9	-2.5040565
1025	4930579K19Rik	75881	chr9	-3.9033618
1026	Mras	17532	chr9	-3.103294
1027	Msl2	77853	chr9	-2.077674
1028	Ppp2r3a	235542	chr9	-7.3836555
1029	Rab6b	270192	chr9	-9.705167
1030	Trf	22041	chr9	-2.4495013
1031	Bfsp2	107993	chr9	-2.6124055
1032	Nudt16	75686	chr9	-2.678185
1033	Aste1	66595	chr9	-3.0063236
1034	Glyctk	235582	chr9	-3.5337832
1035	Rrp9	27966	chr9	-2.3607788
1036	Cacna2d2	56808	chr9	-2.2648075
1037	Nprl2	56032	chr9	-12.068364
1038	Tusc2	80385	chr9	-5.532121
1039	Nat6	56441	chr9	-2.8633037
1040	Camkv	235604	chr9	-2.3225124
1041	Traip	22036	chr9	-2.3595922
1042	Uba7	74153	chr9	-3.3011398
1043	Tcta	102791	chr9	-4.08332
1044	Ccdc36	434438	chr9	-2.2430243
1045	Dalrd3	67789	chr9	-2.593279
1046	Ip6k2	76500	chr9	-4.751192
1047	Shisa5	66940	chr9	-2.21033
1048	Ccdc51	66658	chr9	-2.123632

1049	Kif9	16578	chr9	-3.9224782
1050	4930520O04Rik	75116	chr9	-4.7733574
1051	Tgfbr2	21813	chr9	-2.7284768
1052	Eomes	13813	chr9	-6.9716845
1053	Itga9	104099	chr9	-2.9926786
1054	Csrnp1	215418	chr9	-2.5014477
1055	1700048O20Rik	69430	chr9	-6.5592346
1056	Gtdc2	215494	chr9	-3.0976908
1057	Ccr9	12769	chr9	-2.0569038
1058	4930526I15Rik	75135	chr9	-8.451191
1059	Esr1	13982	chr10	-2.507994
1060	Myct1	68632	chr10	-2.5812361
1061	Cnksr3	215748	chr10	-2.4119954
1062	Katna1	23924	chr10	-2.018226
1063	Ginm1	215751	chr10	-2.0719702
1064	Aig1	66253	chr10	-5.5108213
1065	Vta1	66201	chr10	-2.4130669
1066	Tnfaip3	21929	chr10	-4.8744154
1067	Olig3	94222	chr10	-3.1008523
1068	Slc35d3	76157	chr10	-4.2532134
1069	Gm6251	621697	chr10	-2.929892
1070	Tbpl1	237336	chr10	-3.045626
1071	Arg1	11846	chr10	-5.4270873
1072	Echdc1	52665	chr10	-2.1250668
1073	Trmt11	73681	chr10	-2.652073
1074	Fam26f	215900	chr10	-3.7945082
1075	Tspyl4	72480	chr10	-3.0355163
1076	Marcks	17118	chr10	-3.1189327
1077	Tube1	71924	chr10	-2.2628875
1078	Fyn	14360	chr10	-3.5179622
1079	E130307A14Rik	327744	chr10	-2.4673424
1080	Sesn1	140742	chr10	-2.5805125
1081	Scml4	268297	chr10	-2.972945
1082	Pdss2	71365	chr10	-2.4065425
1083	1700021F05Rik	67851	chr10	-5.8782883

1084	Rtn4ip1	170728	chr10	-2.3977532
1085	Gp49a	14727	chr10	-9.7692795
1086	Gopc	94221	chr10	-2.7923973
1087	Asf1a	66403	chr10	-2.0332196
1088	Ccdc138	76138	chr10	-2.786149
1089	Ascc1	69090	chr10	-2.5370016
1090	Chst3	53374	chr10	-3.2069743
1091	Pald1	27355	chr10	-2.6073766
1092	Tspan15	70423	chr10	-2.4614408
1093	Supv3l1	338359	chr10	-2.5324452
1094	Tet1	52463	chr10	-6.6205044
1095	Slc25a16	73132	chr10	-2.2525957
1096	1700040L02Rik	73287	chr10	-6.3168817
1097	Rhobtb1	69288	chr10	-2.2858553
1098	Cisd1	52637	chr10	-2.8914232
1099	Gnaz	14687	chr10	-3.5327847
1100	Gm5779	544707	chr10	-2.6944253
1101	Ggt1	14598	chr10	-2.0304933
1102	Gstt3	103140	chr10	-4.820309
1103	Gstt2	14872	chr10	-4.491651
1104	Derl3	70377	chr10	-2.926759
1105	Mmp11	17385	chr10	-2.95828
1106	Prmt2	15468	chr10	-5.346161
1107	Lss	16987	chr10	-4.1915736
1108	Aire	11634	chr10	-2.5089808
1109	D10Jhu81e	28295	chr10	-2.5894215
1110	Syde1	71709	chr10	-5.054178
1111	2610008E11Rik	72128	chr10	-2.3835065
1112	Ppap2c	50784	chr10	-4.2532163
1113	Med16	216154	chr10	-2.5029693
1114	Abca7	27403	chr10	-2.082128
1115	Adat3	100113398	chr10	-3.041865
1116	Mob3a	208228	chr10	-2.9426303
1117	Mir1982	100316778	chr10	-89.82776
1118	Nmrk2	69564	chr10	-4.51966

1119	Sirt6	50721	chr10	-4.164716
1120	Gm10778	100233208	chr10	-2.2748835
1121	Zfp433	73610	chr10	-3.5498753
1122	Zfp781	331188	chr10	-2.5718346
1123	AU041133	216177	chr10	-7.408721
1124	Nuak1	77976	chr10	-3.2727444
1125	Tcp11l2	216198	chr10	-6.696613
1126	Btbd11	74007	chr10	-2.330988
1127	Timp3	21859	chr10	-2.334426
1128	Slc17a8	216227	chr10	-2.2089205
1129	Cradd	12905	chr10	-6.2614098
1130	Dusp6	67603	chr10	-2.786359
1131	Glipr1	73690	chr10	-2.5430546
1132	Tmem19	67226	chr10	-3.055297
1133	Rab3ip	216363	chr10	-2.7990656
1134	Tmbim4	68212	chr10	-2.9606657
1135	Msrb3	320183	chr10	-7.198526
1136	Gns	75612	chr10	-2.233592
1137	Gm9079	668272	chr10	-3.8703408
1138	Gm4489	100043513	chr10	-5.63964
1139	A130077B15Rik	319272	chr10	-2.4133232
1140	Xrcc6bp1	68876	chr10	-4.382099
1141	Tsfm	66399	chr10	-7.9528475
1142	Agap2	216439	chr10	-2.219453
1143	Dtx3	80904	chr10	-2.2173853
1144	Nab2	17937	chr10	-2.7100916
1145	Rbms2	56516	chr10	-2.410896
1146	Coq10a	210582	chr10	-2.2487085
1147	Suox	211389	chr10	-2.2519119
1148	Dgka	13139	chr10	-3.962335
1149	Tmem198b	73827	chr10	-6.0284967
1150	Patz1	56218	chr11	-2.2704334
1151	Pik3ip1	216505	chr11	-2.9810429
1152	Selm	114679	chr11	-2.9146755
1153	4921536K21Rik	67430	chr11	-4.0525327

1154	Emid1	140703	chr11	-2.415772
1155	Ccdc117	104479	chr11	-2.0188174
1156	Camk2b	12323	chr11	-2.9096172
1157	Nudcd3	209586	chr11	-2.4045916
1158	Upp1	22271	chr11	-8.5502615
1159	Grb10	14783	chr11	-2.1119912
1160	Plek	56193	chr11	-2.9395978
1161	C1d	57316	chr11	-2.1045606
1162	Meis1	17268	chr11	-2.2933407
1163	Cep68	216543	chr11	-6.8833876
1164	Peli1	67245	chr11	-3.4356637
1165	Vps54	245944	chr11	-3.557511
1166	Pus10	74467	chr11	-2.0822363
1167	Bcl11a	14025	chr11	-2.3858585
1168	Ccdc88a	108686	chr11	-2.0472424
1169	Asb3	65257	chr11	-10.608935
1170	Ubtd2	327900	chr11	-2.6351383
1171	Rnf145	74315	chr11	-2.1291301
1172	Ebf1	13591	chr11	-4.267202
1173	Lsm11	72290	chr11	-2.5430849
1174	Nipal4	214112	chr11	-2.384025
1175	Itk	16428	chr11	-5.537549
1176	Med7	66213	chr11	-4.199511
1177	Snord96a	100216534	chr11	-6.970948
1178	Irgm1	15944	chr11	-2.2294068
1179	Tgtp1	21822	chr11	-2.2914362
1180	Tgtp2	100039796	chr11	-2.9651685
1181	Mapk9	26420	chr11	-2.2425578
1182	Zfp2	22678	chr11	-10.193234
1183	N4bp3	212706	chr11	-3.0811632
1184	Ube2b	22210	chr11	-2.246845
1185	Shroom1	71774	chr11	-4.856593
1186	Kif3a	16568	chr11	-3.2959673
1187	Irf1	16362	chr11	-2.37981
1188	Slc22a5	20520	chr11	-4.9427824

1189	Ccdc69	52570	chr11	-3.067469
1190	4921508A21Rik	74045	chr11	-2.3591986
1191	Atox1	11927	chr11	-3.2179737
1192	Irgm2	54396	chr11	-14.159553
1193	Zfp39	22698	chr11	-3.1216395
1194	Hist3h2bb-ps	382522	chr11	-2.4101543
1195	Mrpl55	67212	chr11	-5.6602116
1196	Nlrp3	216799	chr11	-2.5365613
1197	Flcn	216805	chr11	-3.0152533
1198	Rasd1	19416	chr11	-8.262591
1199	Atpaf2	246782	chr11	-3.360568
1200	Dhrs7b	216820	chr11	-6.4948974
1201	Gm16516	24082	chr11	-2.9318411
1202	B9d1	27078	chr11	-10.958384
1203	Prpsap2	212627	chr11	-2.0053682
1204	Trpv2	22368	chr11	-2.280117
1205	Zfp287	170740	chr11	-2.8010564
1206	Zkscan6	52712	chr11	-3.5121238
1207	Stx8	55943	chr11	-4.430361
1208	Pik3r6	104709	chr11	-2.4234338
1209	Myh10	77579	chr11	-2.0402486
1210	2310047M10Rik	71923	chr11	-2.233185
1211	Vamp2	22318	chr11	-2.3884883
1212	Tmem88	67020	chr11	-3.9729736
1213	Fxr2	23879	chr11	-2.0613215
1214	Mpdu1	24070	chr11	-24.698816
1215	Zbtb4	75580	chr11	-6.396974
1216	Nlgn2	216856	chr11	-2.1825244
1217	Kctd11	216858	chr11	-2.2690084
1218	2810408A11Rik	70419	chr11	-6.773856
1219	Rnf167	70510	chr11	-4.7172704
1220	Eno3	13808	chr11	-4.15016
1221	Camta2	216874	chr11	-3.2676864
1222	Zfp3	193043	chr11	-2.1448987
1223	Rpain	69723	chr11	-2.0982082

1224	4933427D14Rik	74477	chr11	-2.0275383
1225	Spns2	216892	chr11	-2.3860793
1226	Cyb5d2	192986	chr11	-2.0968902
1227	P2rx1	18436	chr11	-21.384884
1228	1200014J11Rik	66874	chr11	-2.180823
1229	Gsg2	14841	chr11	-2.002756
1230	Ovca2	246257	chr11	-3.5279615
1231	Rtn4rl1	237847	chr11	-2.1180143
1232	Tlcd2	380712	chr11	-2.2595196
1233	Scarf1	380713	chr11	-3.2427533
1234	Inpp5k	19062	chr11	-2.1805155
1235	Pipox	19193	chr11	-4.1878724
1236	Dhrs13	70451	chr11	-6.7261753
1237	Traf4	22032	chr11	-3.046702
1238	Tlcd1	68385	chr11	-2.1333632
1239	Nlk	18099	chr11	-2.3655581
1240	Ksr1	16706	chr11	-2.9523635
1241	Rhbdl3	246104	chr11	-5.879135
1242	Slfn2	20556	chr11	-5.511859
1243	Pex12	103737	chr11	-2.3886032
1244	1700020L24Rik	66330	chr11	-6.0675216
1245	Ccl3	20302	chr11	-3.1989322
1246	Ccl4	20303	chr11	-3.895199
1247	Wfdc17	100034251	chr11	-6.3296995
1248	Rps6kb1	72508	chr11	-2.310941
1249	Mks1	380718	chr11	-2.599205
1250	Vezf1	22344	chr11	-2.8822875
1251	C030037D09Rik	193280	chr11	-2.5919557
1252	4930405P13Rik	73921	chr11	-5.205009
1253	Scpep1	74617	chr11	-2.0736716
1254	Hlf	217082	chr11	-2.4768999
1255	Stxbp4	20913	chr11	-4.138767
1256	Tom1l1	71943	chr11	-12.143325
1257	Tob1	22057	chr11	-2.7596128
1258	Xylt2	217119	chr11	-2.3815756

1259	Samd14	217125	chr11	-3.9802744
1260	Pdk2	18604	chr11	-2.505399
1261	Gngt2	14710	chr11	-7.5548143
1262	Skap1	78473	chr11	-2.231821
1263	Snx11	74479	chr11	-2.4591625
1264	Snora21	100302498	chr11	-93.49123
1265	Ormdl3	66612	chr11	-12.6307535
1266	Rapgefl1	268480	chr11	-2.421065
1267	Ghdc	80860	chr11	-2.984157
1268	Stat5a	20850	chr11	-2.29385
1269	Ptrf	19285	chr11	-3.098472
1270	Atp6v0a1	11975	chr11	-4.32444
1271	Fam134c	67998	chr11	-2.4967418
1272	Aoc2	237940	chr11	-3.393424
1273	Rundc1	217201	chr11	-3.8764725
1274	Dhx8	217207	chr11	-2.0504594
1275	Mpp2	50997	chr11	-2.104803
1276	Itga2b	16399	chr11	-2.0271292
1277	Dcakd	68087	chr11	-2.464931
1278	Tanc2	77097	chr11	-3.8082664
1279	Snord104	100216537	chr11	-30.830273
1280	Polg2	50776	chr11	-2.8514094
1281	Helz	78455	chr11	-2.6629775
1282	Axin2	12006	chr11	-3.6732268
1283	Gm11696	100036768	chr11	-3.3602543
1284	Map2k6	26399	chr11	-2.2791948
1285	2610035D17Rik	72386	chr11	-2.6448526
1286	Ttyh2	117160	chr11	-2.0419283
1287	Cd300lh	382551	chr11	-7.417629
1288	Armc7	276905	chr11	-3.5443444
1289	Caskin2	140721	chr11	-2.0701768
1290	Unc13d	70450	chr11	-3.2508132
1291	Wbp2	22378	chr11	-3.2205777
1292	Trim47	217333	chr11	-3.577552
1293	Trim65	338364	chr11	-2.9643607

1294	Ten1	69535	chr11	-2.6392188
1295	Foxj1	15223	chr11	-2.1992698
1296	Rnf157	217340	chr11	-2.619733
1297	Rhbdf2	217344	chr11	-2.9328606
1298	Jmjd6	107817	chr11	-2.901112
1299	Mettl23	74319	chr11	-10.250976
1300	Mfsd11	69900	chr11	-2.0112772
1301	Afmid	71562	chr11	-7.6291513
1302	Timp2	21858	chr11	-2.099756
1303	Engase	217364	chr11	-2.043806
1304	Cbx2	12416	chr11	-5.28599
1305	Cbx8	30951	chr11	-4.9958715
1306	Ccdc40	207607	chr11	-2.6308503
1307	0610009L18Rik	66838	chr11	-3.7901297
1308	Aspscr1	68938	chr11	-2.029943
1309	Rac3	170758	chr11	-2.1763358
1310	Ccdc57	71276	chr11	-5.1935987
1311	Cd7	12516	chr11	-4.323724
1312	1110002L01Rik	100043040	chr12	-2.4290328
1313	Efr3b	668212	chr12	-2.3341486
1314	Ncoa1	17977	chr12	-2.5623105
1315	Itsn2	20403	chr12	-2.4418707
1316	Fkbp1b	14226	chr12	-3.5909538
1317	Sdc1	20969	chr12	-2.1154962
1318	Ttc32	75516	chr12	-2.712728
1319	Vsnl1	26950	chr12	-4.350783
1320	Lpin1	14245	chr12	-3.3219151
1321	Asap2	211914	chr12	-5.171865
1322	Itgb1bp1	16413	chr12	-2.4644387
1323	Klf11	194655	chr12	-2.4869394
1324	Gm20187	100504347	chr12	-2.4663532
1325	Rnaseh1	19819	chr12	-2.3365972
1326	Hbp1	73389	chr12	-2.916809
1327	Tspan13	66109	chr12	-2.6588016
1328	Gm5434	432649	chr12	-2.2232075

1329	Agmo	319660	chr12	-3.0845742
1330	Arhgap5	11855	chr12	-4.0690923
1331	Prpf39	328110	chr12	-2.3755455
1332	Fancm	104806	chr12	-2.6481233
1333	5830428M24Rik	76062	chr12	-5.095272
1334	Mnat1	17420	chr12	-2.7633386
1335	Slc38a6	625098	chr12	-4.188419
1336	2210039B01Rik	73644	chr12	-3.831095
1337	Zbtb25	109929	chr12	-3.157572
1338	Gm10451	100041694	chr12	-9.852002
1339	Mpp5	56217	chr12	-5.0317707
1340	Rdh12	77974	chr12	-2.3279903
1341	Zfyve26	211978	chr12	-2.1956985
1342	Actn1	109711	chr12	-2.349748
1343	Zfyve1	217695	chr12	-2.8082461
1344	Aldh6a1	104776	chr12	-3.6315837
1345	Lin52	217708	chr12	-2.2214804
1346	Fos	14281	chr12	-2.0261757
1347	Angel1	68737	chr12	-2.6890154
1348	Pomt2	217734	chr12	-2.8562386
1349	Tmed8	382620	chr12	-3.1312356
1350	Gpr65	14744	chr12	-2.0127084
1351	Ptpn21	24000	chr12	-3.4871628
1352	Tdp1	104884	chr12	-2.2807117
1353	Golga5	27277	chr12	-2.3971384
1354	AK010878	100233175	chr12	-3.9449358
1355	Wdr20a	69641	chr12	-2.9769695
1356	Snora28	100316932	chr12	-3.4230545
1357	Ppp1r13b	21981	chr12	-2.2943997
1358	BC022687	217887	chr12	-3.4711516
1359	Gpr132	56696	chr12	-2.04275
1360	Pacs2	217893	chr12	-2.1960042
1361	Tmem121	69195	chr12	-4.2725463
1362	Zfp386	56220	chr12	-2.2707002
1363	Vipr2	22355	chr12	-5.775315

1364	Rapgef5	217944	chr12	-2.4324257
1365	Akr1c12	622402	chr13	-2.5721018
1366	Akr1e1	56043	chr13	-4.605266
1367	Dip2c	208440	chr13	-4.775607
1368	Gm10336	328186	chr13	-2.124788
1369	Ggps1	14593	chr13	-2.2086194
1370	AW209491	105351	chr13	-3.0455587
1371	Epdr1	105298	chr13	-2.4571533
1372	Aoah	27052	chr13	-2.1526625
1373	Hist1h2bm	319186	chr13	-2.9305496
1374	Hist1h4m	100041230	chr13	-2.499647
1375	Hist1h4n	319161	chr13	-2.4996471
1376	Prss16	54373	chr13	-3.3271492
1377	Hist1h4i	319158	chr13	-3.409438
1378	Hist1h3c	319148	chr13	-2.0324063
1379	Irf4	16364	chr13	-2.170914
1380	Serpinb6b	20708	chr13	-7.15143
1381	Serpinb9	20723	chr13	-9.381139
1382	Tubb2b	73710	chr13	-4.851609
1383	Slc35b3	108652	chr13	-2.106778
1384	Gcnt2	14538	chr13	-3.9331641
1385	Nedd9	18003	chr13	-2.36501
1386	Id4	15904	chr13	-2.9443903
1387	Ptpdc1	218232	chr13	-3.6734188
1388	Fgd3	30938	chr13	-2.75743
1389	Cenpp	66336	chr13	-2.596873
1390	Fbxw17	109082	chr13	-10.952803
1391	Gadd45g	23882	chr13	-4.525637
1392	Nfil3	18030	chr13	-2.1710887
1393	Cplx2	12890	chr13	-2.80093
1394	Tspan17	74257	chr13	-6.0253863
1395	Unc5a	107448	chr13	-2.300602
1396	Pdlim7	67399	chr13	-6.005138
1397	Txndc15	69672	chr13	-2.1497087
1398	Pcbd2	72562	chr13	-2.3279898

1399	Ctla2b	13025	chr13	-4.0042076
1400	Ctla2a	13024	chr13	-3.77447
1401	Zfp935	71508	chr13	-2.6225572
1402	Mir27b	387221	chr13	-60.726303
1403	Ctsl	13039	chr13	-3.638122
1404	Zfp369	170936	chr13	-2.0962446
1405	Gm10324	628709	chr13	-4.908053
1406	Zfp874a	238692	chr13	-7.587522
1407	Zfp87	170763	chr13	-3.0122828
1408	Zfp493	72958	chr13	-4.381791
1409	Papd7	210106	chr13	-3.7447028
1410	Srd5a1	78925	chr13	-2.8610854
1411	Zfp825	235956	chr13	-2.3890872
1412	Ell2	192657	chr13	-3.9533525
1413	Rhobtb3	73296	chr13	-2.8398552
1414	Arrdc3	105171	chr13	-2.3880436
1415	Xrcc4	108138	chr13	-8.86707
1416	Msh3	17686	chr13	-2.501104
1417	Fam151b	73942	chr13	-2.685931
1418	Serinc5	218442	chr13	-2.1558836
1419	Gm9776	328309	chr13	-3.5182464
1420	Zbed3	72114	chr13	-2.2214808
1421	F2rl1	14063	chr13	-2.283364
1422	F2r	14062	chr13	-5.358585
1423	Enc1	13803	chr13	-2.1266072
1424	Fcho2	218503	chr13	-4.273905
1425	Ptcd2	68927	chr13	-2.7388132
1426	Mccc2	78038	chr13	-3.1822386
1427	Serf1	20365	chr13	-2.1910496
1428	Marveld2	218518	chr13	-2.2753205
1429	Mrps36	66128	chr13	-9.859722
1430	Sgtb	218544	chr13	-2.0237377
1431	Trim23	81003	chr13	-2.4282248
1432	Cenpk	60411	chr13	-2.3214686
1433	Srek1ip1	67288	chr13	-2.1606183

1434	Plk2	20620	chr13	-2.6528006
1435	Ddx4	13206	chr13	-8.852394
1436	Gzma	14938	chr13	-12.024378
1437	Arl15	218639	chr13	-2.2348707
1438	Mocs2	17434	chr13	-3.2695274
1439	Itga2	16398	chr13	-5.330337
1440	Isl1	16392	chr13	-2.39934
1441	4833420G17Rik	67392	chr13	-2.3937347
1442	Abhd6	66082	chr14	-2.8095727
1443	Rpp14	67053	chr14	-2.1018794
1444	Kcnk5	16529	chr14	-4.0745826
1445	Nudt13	67725	chr14	-2.1266074
1446	Fam149b	105428	chr14	-3.5906096
1447	Mrps16	66242	chr14	-14.507625
1448	Ndst2	17423	chr14	-2.1910503
1449	Pde12	211948	chr14	-2.1218584
1450	Arhgef3	71704	chr14	-2.4373052
1451	Nek4	23955	chr14	-7.4197755
1452	Phf7	71838	chr14	-6.208152
1453	Btd	26363	chr14	-3.3222182
1454	Vstm4	320736	chr14	-3.2073781
1455	Tspan14	52588	chr14	-2.234642
1456	Ear1	13586	chr14	-2.8848822
1457	Ktn1	16709	chr14	-3.2028883
1458	Rnase6	78416	chr14	-5.0535493
1459	AY358078	278676	chr14	-2.007264
1460	Zfp219	69890	chr14	-2.0235806
1461	Rpgrip1	77945	chr14	-2.4703004
1462	Rab2b	76338	chr14	-10.320996
1463	Oxa1l	69089	chr14	-2.6546202
1464	Mrpl52	68836	chr14	-15.65958
1465	4931414P19Rik	74359	chr14	-2.9031403
1466	Slc7a8	50934	chr14	-6.3963513
1467	Homez	239099	chr14	-3.9492996
1468	Ap1g2	11766	chr14	-2.1110785

1469	Rec8	56739	chr14	-8.490316
1470	Ltb4r1	16995	chr14	-2.4539757
1471	Nynrin	277154	chr14	-2.3991992
1472	Mcpt8	17231	chr14	-5.2764063
1473	Zmym5	219105	chr14	-2.2846968
1474	Zmym2	76007	chr14	-2.0714433
1475	Mrp63	67840	chr14	-6.7387342
1476	Atp8a2	50769	chr14	-2.0399415
1477	Blk	12143	chr14	-5.6065083
1478	Kif13b	16554	chr14	-2.0855596
1479	R3hcc1	71843	chr14	-2.9394777
1480	9930012K11Rik	268759	chr14	-3.6901886
1481	Pdlim2	213019	chr14	-6.9542017
1482	Slc39a14	213053	chr14	-4.3055296
1483	Bmp1	12153	chr14	-2.347553
1484	Dok2	13449	chr14	-2.4263844
1485	Rcbtb2	105670	chr14	-2.8479445
1486	5031414D18Rik	271221	chr14	-10.1772995
1487	Tsc22d1	21807	chr14	-2.3929608
1488	Lacc1	210808	chr14	-2.529666
1489	Dach1	13134	chr14	-2.9135518
1490	Uchl3	50933	chr14	-2.2541425
1491	Ndfip2	76273	chr14	-3.4567528
1492	Tgds	76355	chr14	-4.781452
1493	Dzip1	66573	chr14	-2.0529163
1494	Card6	239319	chr15	-3.5768337
1495	Prkaa1	105787	chr15	-2.2472303
1496	Ttc33	67515	chr15	-2.0693247
1497	5430437J10Rik	71432	chr15	-2.183952
1498	Fyb	23880	chr15	-3.526468
1499	Sdc2	15529	chr15	-3.2865741
1500	Laptm4b	114128	chr15	-2.1910489
1501	Matn2	17181	chr15	-3.000968
1502	Stk3	56274	chr15	-2.200399
1503	Fbxo43	78803	chr15	-4.9193

1504	Slc25a32	69906	chr15	-3.962536
1505	Angpt1	11600	chr15	-3.2278013
1506	Samd12	320679	chr15	-3.9771335
1507	Mtbp	105837	chr15	-2.148916
1508	Zhx2	387609	chr15	-2.3764458
1509	Fbxo32	67731	chr15	-2.8240755
1510	Trmt12	68260	chr15	-2.885545
1511	Mtss1	211401	chr15	-4.686411
1512	Sqle	20775	chr15	-2.3843942
1513	Pvt1	19296	chr15	-2.3532133
1514	St3gal1	20442	chr15	-2.1340282
1515	Khdrbs3	13992	chr15	-2.2440603
1516	Peg13	353342	chr15	-6.4847193
1517	Ptk2	14083	chr15	-3.7821686
1518	Ly6a	110454	chr15	-2.2855234
1519	9030619P08Rik	105892	chr15	-10.198339
1520	Top1mt	72960	chr15	-3.4995928
1521	Naprt1	223646	chr15	-4.5514975
1522	Grina	66168	chr15	-2.8197525
1523	Sharpin	106025	chr15	-2.354239
1524	Hsf1	15499	chr15	-3.0062451
1525	Dgat1	13350	chr15	-2.116173
1526	Kifc2	16581	chr15	-2.6747766
1527	Ppp1r16a	73062	chr15	-3.2389429
1528	C030006K11Rik	223665	chr15	-2.6452913
1529	Zfp647	239546	chr15	-4.083708
1530	Rbfox2	93686	chr15	-2.550791
1531	Kctd17	72844	chr15	-2.1472282
1532	Il2rb	16185	chr15	-5.8014092
1533	Mfng	17305	chr15	-2.1339395
1534	Pdxp	57028	chr15	-2.128503
1535	Gcat	26912	chr15	-2.7582524
1536	Pla2g6	53357	chr15	-4.0938487
1537	Csnk1e	27373	chr15	-2.0240357
1538	D730005E14Rik	109361	chr15	-2.5475056

1539	Pdgfb	18591	chr15	-3.1233356
1540	Syngr1	20972	chr15	-2.320694
1541	Srebf2	20788	chr15	-2.140333
1542	Fam109b	338368	chr15	-2.0621645
1543	Tbrg3	21378	chr15	-2.1649787
1544	Parvb	170736	chr15	-5.17541
1545	Gramd4	223752	chr15	-2.5744832
1546	Pim3	223775	chr15	-2.1592958
1547	1300018J18Rik	223776	chr15	-2.0336847
1548	Mapk12	29857	chr15	-5.039844
1549	Ppp6r2	71474	chr15	-2.9414089
1550	BC090627	100134861	chr15	-2.2187843
1551	Chkb	12651	chr15	-2.277447
1552	Cpne8	66871	chr15	-2.2282836
1553	Prickle1	106042	chr15	-3.3023298
1554	Pced1b	239647	chr15	-8.264636
1555	Rapgef3	223864	chr15	-2.1216228
1556	Adcy6	11512	chr15	-2.0792663
1557	Lmbr1l	74775	chr15	-5.9388976
1558	Bcdin3d	75284	chr15	-11.2289915
1559	Mettl7a3	668178	chr15	-3.5604196
1560	Mettl7a2	393082	chr15	-3.388506
1561	Tfcp2	21422	chr15	-9.248063
1562	Pou6f1	19009	chr15	-2.7777023
1563	Grasp	56149	chr15	-3.9468918
1564	Krt80	74127	chr15	-2.5347648
1565	Tenc1	209039	chr15	-2.1183095
1566	Soat2	223920	chr15	-3.8807228
1567	Npff	54615	chr15	-3.6517494
1568	Calcoco1	67488	chr15	-2.0180712
1569	Crebbp	12914	chr16	-2.120495
1570	Gm5766	436332	chr16	-6.2301326
1571	Adcy9	11515	chr16	-2.2219095
1572	Glis2	83396	chr16	-3.1282358
1573	Carhsp1	52502	chr16	-7.7371435

1574	Socs1	12703	chr16	-2.7741916
1575	Snx29	74478	chr16	-3.4034312
1576	Cpped1	223978	chr16	-2.4037998
1577	Fopnl	66086	chr16	-2.064643
1578	Ypel1	106369	chr16	-4.7878494
1579	P2rx6	18440	chr16	-3.7501438
1580	Dgcr14	27886	chr16	-6.1764917
1581	Slc25a1	13358	chr16	-4.256386
1582	Vpreb2	22363	chr16	-4.557285
1583	Dgcr6	13353	chr16	-2.6336193
1584	Klhl24	75785	chr16	-2.2100716
1585	Abcf3	27406	chr16	-2.0224378
1586	Ece2	107522	chr16	-2.2753208
1587	1300002E11Rik	100043489	chr16	-2.1850965
1588	Igf2bp2	319765	chr16	-2.1060054
1589	Etv5	104156	chr16	-2.6655257
1590	St6gal1	20440	chr16	-3.1509378
1591	Lpp	210126	chr16	-3.6237338
1592	Mb21d2	239796	chr16	-2.4040682
1593	Hes1	15205	chr16	-5.2548532
1594	AI480653	268880	chr16	-2.5406852
1595	Dlg1	13383	chr16	-2.2818506
1596	0610012G03Rik	106264	chr16	-7.0441184
1597	Cep19	66994	chr16	-3.797819
1598	Wdr53	68980	chr16	-2.2141135
1599	Slc12a8	171286	chr16	-2.313633
1600	Muc13	17063	chr16	-2.040632
1601	Itgb5	16419	chr16	-5.7186837
1602	Mylk	107589	chr16	-2.4535453
1603	Parp14	547253	chr16	-2.5960217
1604	Wdr5b	69544	chr16	-17.086372
1605	Cd86	12524	chr16	-8.316192
1606	Eaf2	106389	chr16	-2.6728387
1607	Gtf2e1	74197	chr16	-2.2601082
1608	Zbtb20	56490	chr16	-3.362231

1609	Tigit	100043314	chr16	-6.487077
1610	2610015P09Rik	212153	chr16	-7.156682
1611	Cd200r1	57781	chr16	-4.2360296
1612	Cd200r3	74603	chr16	-5.137941
1613	Cd96	84544	chr16	-3.1628833
1614	Nfkbiz	80859	chr16	-2.7295456
1615	Nxpe3	385658	chr16	-2.0462544
1616	Cpox	12892	chr16	-2.1810653
1617	Rwdd2b	53858	chr16	-3.153335
1618	Il10rb	16155	chr16	-2.3855853
1619	A930006K02Rik	100503120	chr16	-2.764245
1620	Donson	60364	chr16	-2.5091047
1621	Itsn1	16443	chr16	-4.304937
1622	Pigp	56176	chr16	-3.9146242
1623	Wrb	71446	chr16	-2.3475528
1624	Tmem242	70544	chr17	-2.3170838
1625	Tulp4	68842	chr17	-2.061521
1626	Dynlt1b	21648	chr17	-3.4338121
1627	Rsph3a	66832	chr17	-2.271927
1628	Mpc1	55951	chr17	-2.3315015
1629	Prr18	320111	chr17	-2.3110936
1630	1700010I14Rik	66931	chr17	-5.6216216
1631	C030013G03Rik	77414	chr17	-4.581285
1632	Igf2r	16004	chr17	-8.976235
1633	Phf10	72057	chr17	-2.4472375
1634	Gm3435	100041621	chr17	-4.9915347
1635	2210404J11Rik	381062	chr17	-5.777106
1636	Tbp	21374	chr17	-2.9494877
1637	Zfp97	22759	chr17	-2.6499503
1638	Zfp677	210503	chr17	-10.016228
1639	Zfp54	22712	chr17	-2.5942361
1640	Zfp53	24132	chr17	-3.2189987
1641	Zfp942	73233	chr17	-2.0252795
1642	Zfp945	240041	chr17	-3.1467457
1643	Zfp213	449521	chr17	-3.8001018

1644	Flywch1	224613	chr17	-2.9636269
1645	1600002H07Rik	72016	chr17	-2.3032696
1646	Ccnf	12449	chr17	-2.034548
1647	Eci1	13177	chr17	-3.646378
1648	Dnase1l2	66705	chr17	-2.3726633
1649	Pkd1	18763	chr17	-2.0352166
1650	Gfer	11692	chr17	-2.5599623
1651	Snora78	100306952	chr17	-17.433792
1652	Snora64	104366	chr17	-2.0599608
1653	Rpl3l	66211	chr17	-3.677257
1654	Ift140	106633	chr17	-2.0706623
1655	Rpusd1	106707	chr17	-4.8216763
1656	Metrn	70083	chr17	-22.519941
1657	0610011F06Rik	68347	chr17	-4.8213935
1658	Rab40c	224624	chr17	-4.468198
1659	Mrpl28	68611	chr17	-2.937998
1660	Phf1	21652	chr17	-2.0372243
1661	Zbtb9	474156	chr17	-2.7997966
1662	Snrpc	20630	chr17	-3.0518196
1663	Scube3	268935	chr17	-5.1779156
1664	Mapk13	26415	chr17	-6.1976438
1665	Brpf3	268936	chr17	-2.3098555
1666	4930539E08Rik	207819	chr17	-2.9033513
1667	Zfand3	21769	chr17	-2.4719532
1668	Slc37a1	224674	chr17	-3.5056834
1669	Cyp4f16	70101	chr17	-3.3157887
1670	Gm9705	677156	chr17	-2.626995
1671	Zfp870	240066	chr17	-3.0758843
1672	Zfp952	240067	chr17	-2.9877949
1673	Zfp81	224694	chr17	-2.6730814
1674	H2-K2	630499	chr17	-2.7868314
1675	BC051537	414076	chr17	-5.157272
1676	H2-DMb1	14999	chr17	-7.340015
1677	H2-Ob	15002	chr17	-2.6844532
1678	H2-Ab1	14961	chr17	-3.5604556

1679	H2-Aa	14960	chr17	-6.900099
1680	H2-Eb1	14969	chr17	-3.6735253
1681	Fkbpl	56299	chr17	-2.0084627
1682	Zbtb12	193736	chr17	-2.5101342
1683	1110038B12Rik	68763	chr17	-3.3084857
1684	Msh5	17687	chr17	-4.5168233
1685	Lst1	16988	chr17	-8.459459
1686	Ltb	16994	chr17	-4.601204
1687	Tnf	21926	chr17	-2.7710333
1688	H2-Q10	15007	chr17	-2.143784
1689	Pou5f1	18999	chr17	-3.2516842
1690	Cchcr1	240084	chr17	-9.411419
1691	Dpcr1	268949	chr17	-2.0073602
1692	2310061I04Rik	69662	chr17	-2.5562246
1693	Atat1	73242	chr17	-2.272199
1694	Mir1894	100316810	chr17	-17.007977
1695	H2-T22	15039	chr17	-2.1954029
1696	Gm11127	100529082	chr17	-2.3670132
1697	H2-Bl	14963	chr17	-4.229594
1698	C920025E04Rik	667803	chr17	-2.0623252
1699	Trim39	79263	chr17	-3.8824275
1700	Gabbr1	54393	chr17	-2.3696826
1701	Rhag	19743	chr17	-2.7206597
1702	Cd2ap	12488	chr17	-2.8403804
1703	Slc25a27	74011	chr17	-4.465041
1704	Enpp5	83965	chr17	-9.092856
1705	Nfkbie	18037	chr17	-2.5815864
1706	Tmem63b	224807	chr17	-3.177022
1707	Mad2l1bp	66591	chr17	-6.7628384
1708	Yipf3	28064	chr17	-3.5604548
1709	Taf8	63856	chr17	-3.114759
1710	Mdfi	17240	chr17	-2.6214318
1711	Tbc1d5	72238	chr17	-2.0332386
1712	Satb1	20230	chr17	-2.4967186
1713	Zfp119a	104349	chr17	-2.8390868

1714	Ebi3	50498	chr17	-7.513098
1715	Stap2	106766	chr17	-4.421625
1716	2410015M20Rik	224904	chr17	-2.4375396
1717	Slc25a23	66972	chr17	-4.907038
1718	Gpr108	78308	chr17	-2.5777059
1719	Nudt12	67993	chr17	-4.2135572
1720	Ankrd12	106585	chr17	-3.0980268
1721	Rab12	19328	chr17	-5.1695065
1722	Arhgap28	268970	chr17	-2.538103
1723	Epb4.1l3	13823	chr17	-2.4983306
1724	C030034I22Rik	77533	chr17	-2.665777
1725	Snord53	100217456	chr17	-13.105156
1726	Ypel5	383295	chr17	-2.2491078
1727	1110001A16Rik	68554	chr17	-3.9351826
1728	Galm	319625	chr17	-3.4834301
1729	Gemin6	67242	chr17	-2.5562243
1730	Morn2	378462	chr17	-4.382101
1731	Dync2li1	213575	chr17	-5.3038945
1732	Camkmt	73582	chr17	-4.619357
1733	Rhoq	104215	chr17	-4.238789
1734	Socs5	56468	chr17	-4.8284245
1735	4833418N02Rik	74597	chr17	-2.5556538
1736	4930480K15Rik	78800	chr17	-3.0673811
1737	Gm6225	633947	chr18	-2.990115
1738	Bambi	68010	chr18	-2.747858
1739	Map3k8	26410	chr18	-13.02162
1740	9430020K01Rik	240185	chr18	-2.614125
1741	Zeb1	21417	chr18	-2.1085591
1742	Arhgap12	75415	chr18	-4.246606
1743	Mpp7	75739	chr18	-4.2954164
1744	Colec12	140792	chr18	-3.1042836
1745	Thoc1	225160	chr18	-2.2168906
1746	Cables1	63955	chr18	-6.733666
1747	Impact	16210	chr18	-2.0121279
1748	Zfp521	225207	chr18	-3.962536

1749	Kctd1	106931	chr18	-2.2525802
1750	2700062C07Rik	68046	chr18	-4.36395
1751	Map3k2	26405	chr18	-2.153702
1752	Gypc	71683	chr18	-2.418599
1753	2410004N09Rik	69749	chr18	-2.4029088
1754	Cdc25c	12532	chr18	-3.5366316
1755	Egr1	13653	chr18	-3.7075992
1756	Snhg4	100503380	chr18	-2.693855
1757	Spata24	71242	chr18	-3.9438891
1758	Dnajc18	76594	chr18	-2.5351274
1759	Tmem173	72512	chr18	-3.888646
1760	Pura	19290	chr18	-3.825995
1761	6330403M23Rik	109169	chr18	-13.620262
1762	Hbegf	15200	chr18	-3.1436806
1763	Eif4ebp3	108112	chr18	-8.492941
1764	Apbb3	225372	chr18	-5.3096123
1765	Pcdhgb4	93701	chr18	-4.653624
1766	A930012L18Rik	626275	chr18	-3.4014144
1767	Ccdc112	240261	chr18	-5.3732886
1768	Dtwd2	68857	chr18	-2.1135948
1769	Csnk1g3	70425	chr18	-2.2173853
1770	Aldh7a1	110695	chr18	-2.1325116
1771	Phax	56698	chr18	-2.2768254
1772	Iigp1	60440	chr18	-2.7822852
1773	Cd74	16149	chr18	-3.5633378
1774	Camk2a	12322	chr18	-2.257266
1775	Afap1l1	106877	chr18	-3.636236
1776	Adrb2	11555	chr18	-2.2069268
1777	Apcdd1	494504	chr18	-6.426113
1778	Spire1	68166	chr18	-4.979252
1779	Ska1	66468	chr18	-2.4898288
1780	Slc14a1	108052	chr18	-2.5093026
1781	Nfatc1	18018	chr18	-3.0923324
1782	Atp9b	50771	chr18	-2.8389544
1783	Mbp	17196	chr18	-4.498362

1784	Zfp236	329002	chr18	-2.7005959
1785	2210420H20Rik	76961	chr18	-2.8360243
1786	Tshz1	110796	chr18	-2.697135
1787	Ighmbp2	20589	chr19	-3.175872
1788	Chka	12660	chr19	-2.582308
1789	Nudt8	66387	chr19	-2.0541089
1790	Rps6kb2	58988	chr19	-3.9060519
1791	Clcf1	56708	chr19	-5.258148
1792	Lrfn4	225875	chr19	-2.1532726
1793	Ctsf	56464	chr19	-4.2566195
1794	AI837181	107242	chr19	-4.8086
1795	Ccdc85b	240514	chr19	-2.0598638
1796	Ctsw	13041	chr19	-13.806131
1797	Snx32	225861	chr19	-3.199214
1798	Kat5	81601	chr19	-2.0646915
1799	Ltbp3	16998	chr19	-3.4269705
1800	Tigd3	332359	chr19	-4.7504997
1801	Syvn1	74126	chr19	-2.4383965
1802	Zfpl1	81909	chr19	-5.862057
1803	Vegfb	22340	chr19	-2.2062526
1804	Pla2g16	225845	chr19	-4.2255936
1805	Ttc9c	70387	chr19	-2.8945289
1806	5730408K05Rik	67531	chr19	-2.176245
1807	Rom1	19881	chr19	-6.811306
1808	Stxbp3b	619371	chr19	-2.7753308
1809	Rab3il1	74760	chr19	-5.1124477
1810	Fads3	60527	chr19	-2.5000432
1811	Ms4a4b	60361	chr19	-8.314508
1812	Gm5512	433224	chr19	-2.3570383
1813	Cep78	208518	chr19	-4.2161098
1814	Gnaq	14682	chr19	-2.0101912
1815	Aldh1a1	11668	chr19	-3.8631656
1816	Zfand5	22682	chr19	-2.0779037
1817	Vldlr	22359	chr19	-2.069997
1818	9930021J03Rik	240613	chr19	-2.3964083

1819	Ranbp6	240614	chr19	-3.334112
1820	Uhrf2	109113	chr19	-3.1515055
1821	Dkk1	13380	chr19	-3.700163
1822	Prkg1	19091	chr19	-4.401274
1823	Asah2	54447	chr19	-3.740777
1824	Ifit3	15959	chr19	-2.4962456
1825	Slc16a12	240638	chr19	-2.4248993
1826	Rpp30	54364	chr19	-2.9842694
1827	Myof	226101	chr19	-2.16087
1828	Entpd1	12495	chr19	-3.0899415
1829	Blnk	17060	chr19	-2.9584558
1830	Dntt	21673	chr19	-2.1466174
1831	Lcor	212391	chr19	-4.936709
1832	Morn4	226123	chr19	-6.4595
1833	Avpi1	69534	chr19	-2.804411
1834	Nkx2-3	18089	chr19	-4.436874
1835	BC037704	100502982	chr19	-3.04101
1836	Abcc2	12780	chr19	-2.0679028
1837	Dnmbp	71972	chr19	-3.0274181
1838	Bloc1s2a	73689	chr19	-3.2287211
1839	Scd1	20249	chr19	-2.2179694
1840	Dpcd	226162	chr19	-2.1910496
1841	Fgf8	14179	chr19	-2.8903687
1842	Calhm2	72691	chr19	-7.7287016
1843	Dusp5	240672	chr19	-4.9631224
1844	Gpam	14732	chr19	-2.6382246
1845	Tcf7l2	21416	chr19	-2.8938382
1846	Dclre1a	55947	chr19	-2.7326577
1847	Ablim1	226251	chr19	-2.3650393
1848	B230217O12Rik	320879	chr19	-2.4192839
1849	Gfra1	14585	chr19	-2.8218064
1850	Slc18a2	214084	chr19	-5.2768016
1851	Rab11fip2	74998	chr19	-2.1910496
1852	Nanos1	332397	chr19	-3.6831863
1853	Sfxn4	94281	chr19	-9.451663

1854	Gm7102	633057	chr19	-2.4767246
1855	Ppp1r3f	54646	chrX	-5.129048
1856	Gpkow	209416	chrX	-2.149568
1857	Praf2	54637	chrX	-2.3063686
1858	Gm6787	627782	chrX	-3.386561
1859	AA414768	245350	chrX	-2.884882
1860	Gpr34	23890	chrX	-3.1932206
1861	Wdr44	72404	chrX	-2.3656983
1862	Lonrf3	74365	chrX	-2.4689198
1863	Rhox8	434768	chrX	-2.0621438
1864	Fam122b	78755	chrX	-2.05762
1865	Cxx1c	72865	chrX	-3.765026
1866	Ncrna00086	320237	chrX	-4.631082
1867	Ddx26b	236790	chrX	-2.0884695
1868	Slc9a6	236794	chrX	-2.0201504
1869	Mtmr1	53332	chrX	-2.1344428
1870	Cd99l2	171486	chrX	-2.3127742
1871	Xlr3b	574437	chrX	-2.5390584
1872	Zfp275	27081	chrX	-2.5294576
1873	Bgn	12111	chrX	-3.5399964
1874	Dusp9	75590	chrX	-2.753372
1875	Pnck	93843	chrX	-8.368067
1876	Mecp2	17257	chrX	-2.463139
1877	Snora70	104368	chrX	-4.0107775
1878	Plxna3	18846	chrX	-2.5843043
1879	Lage3	66192	chrX	-5.4776225
1880	Fam3a	66294	chrX	-5.649225
1881	Brcc3	210766	chrX	-2.0135624
1882	Pdk3	236900	chrX	-2.4371867
1883	Gm14827	100503393	chrX	-5.0949674
1884	Maged1	94275	chrX	-2.5294874
1885	Gspt2	14853	chrX	-5.295037
1886	Zxdb	668166	chrX	-3.0481045
1887	Zxda	668171	chrX	-2.6248329
1888	Ar	11835	chrX	-2.3468723

1889	Awat2	245532	chrX	-3.9273322
1890	Awat1	245533	chrX	-4.7650576
1891	Pdzd11	72621	chrX	-2.5040565
1892	Il2rg	16186	chrX	-3.2705467
1893	Phka1	18679	chrX	-5.307316
1894	Gm9159	668415	chrX	-2.0836372
1895	Zdhhc15	108672	chrX	-4.8983364
1896	5530601H04Rik	71445	chrX	-2.6386125
1897	Atp7a	11977	chrX	-3.1489632
1898	A630033H20Rik	213438	chrX	-2.118618
1899	Klhl4	237010	chrX	-2.270724
1900	Xkrx	331524	chrX	-2.0432155
1901	Armcx1	78248	chrX	-2.628054
1902	Armcx6	278097	chrX	-2.6890154
1903	Gprasp1	67298	chrX	-3.1683362
1904	Gprasp2	245607	chrX	-2.868446
1905	Bex2	12069	chrX	-2.2906425
1906	Bex4	406217	chrX	-3.5429742
1907	Tceal8	66684	chrX	-2.5105777
1908	Tceal5	331532	chrX	-4.4258614
1909	BC065397	436230	chrX	-3.6968253
1910	Plp1	18823	chrX	-20.410488
1911	Rab9b	319642	chrX	-3.67391
1912	Ammecr1	56068	chrX	-3.708365
1913	Mageb16-ps1	100039436	chrX	-2.189774
1914	Maged2	80884	chrX	-4.642938
1915	Fgd1	14163	chrX	-2.623138
1916	Tsr2	69499	chrX	-3.8006823
1917	Fam120c	207375	chrX	-2.4831896
1918	Kctd12b	207474	chrX	-3.4841464
1919	2210013O21Rik	70123	chrX	-9.251108
1920	Prdx4	53381	chrX	-6.461742
1921	Gm6568	625253	chrX	-2.2232707
1922	Ctps2	55936	chrX	-3.820028
1923	Bmx	12169	chrX	-3.448219

1924	Mospd2	76763	chrX	-6.306693
1925	Arhgap6	11856	chrX	-3.565826
1926	G530011O06Rik	654820	chrX	-3.4598145
