# Supplementary material for: Impaired Repopulating Ability of Uhrf2−/− Hematopoietic Progenitor Cells in Mice
Source: Genes (Basel). 2023 Jul 27;14(8):1531. doi: 10.3390/genes14081531 (PMC10454722; doi:10.3390/genes14081531)
Supplement: Supplementary file 1 [file genes-14-01531-s001.zip › Supplemental materials/Table S3.pdf]

Table S3. Upregulated canonical pathways in *Uhrf2*$^{-/-}$ LSK cells in RNA sequencing determined by IPA™ (Ingenuity Pathways Analysis, Qiagen).

No.	**Ingenuity Canonical Pathways**	**-log(p-value)**	**z-score**
1	PD-1, PD-L1 cancer immunotherapy pathway	2.41	3.273
2	Oxidative Phosphorylation	1.63	3.273
3	Kinetochore Metaphase Signaling Pathway	2.87	2.294
4	Cyclins and Cell Cycle Regulation	1.63	2.111
