# Supplementary material for: Impaired Repopulating Ability of Uhrf2−/− Hematopoietic Progenitor Cells in Mice
Source: Genes (Basel). 2023 Jul 27;14(8):1531. doi: 10.3390/genes14081531 (PMC10454722; doi:10.3390/genes14081531)
Supplement: Supplementary file 1 [file genes-14-01531-s001.zip › Supplemental materials/Table S4.pdf]

Table S4. Downregulated canonical pathways in *Uhrf2*$^{-/-}$ LSK cells in RNA sequencing determined by IPA™.

No.	Ingenuity Canonical Pathways	-log(p-value)	z-score
1	Thrombin Signaling	2.13	-4.642
2	Integrin Signaling	1.30	-3.780
3	Regulation Of The Epithelial Mesenchymal Transition By Growth Factors Pathway	2.10	-3.656
4	CXCR4 Signaling	1.54	-3.545
5	Role Of Osteoclasts In Rheumatoid Arthritis Signaling Pathway	1.41	-3.507
6	HIF1α Signaling	2.75	-3.479
7	Ephrin Receptor Signaling	1.77	-3.441
8	Senescence Pathway	3.06	-3.051
9	Apelin Endothelial Signaling Pathway	1.52	-2.982
10	IL-8 Signaling	2.93	-2.959
11	Reelin Signaling in Neurons	1.87	-2.858
12	HER-2 Signaling in Breast Cancer	2.77	-2.846
13	Glioma Signaling	3.83	-2.840
14	TGF-β Signaling	1.43	-2.840
15	ERBB Signaling	2.17	-2.828
16	Myelination Signaling Pathway	2.00	-2.722
17	UVB-Induced MAPK Signaling	1.37	-2.714
18	GADD45 Signaling	1.98	-2.673
19	Chemokine Signaling	1.51	-2.673
20	Acute Myeloid Leukemia Signaling	1.37	-2.673
21	Leukocyte Extravasation Signaling	1.63	-2.646
22	Signaling by Rho Family GTPases	1.37	-2.611
23	Cardiac Hypertrophy Signaling (Enhanced)	2.19	-2.557
24	Wound Healing Signaling Pathway	1.39	-2.530
25	Neuroinflammation Signaling Pathway	2.68	-2.380
26	Pathogen Induced Cytokine Storm Signaling Pathway	1.65	-2.364
27	IL-7 Signaling Pathway	2.72	-2.357
28	PEDF Signaling	1.38	-2.309
29	RANK Signaling in Osteoclasts	3.42	-2.236
30	Sphingosine-1-phosphate Signaling	1.54	-2.236

31	Superpathway of Inositol Phosphate Compounds	1.85	-2.197
32	Granzyme A Signaling	2.16	-2.183
33	LPS-stimulated MAPK Signaling	1.34	-2.138
34	Macrophage Alternative Activation Signaling Pathway	2.22	-2.137
35	Inhibition of Angiogenesis by TSP1	2.27	-2.121
36	Tumor Microenvironment Pathway	1.85	-2.121
37	Thrombopoietin Signaling	1.45	-2.111
38	Xenobiotic Metabolism General Signaling Pathway	2.79	-2.043
39	Multiple Sclerosis Signaling Pathway	2.47	-2.030
