# Supplementary material for: Impaired Repopulating Ability of Uhrf2−/− Hematopoietic Progenitor Cells in Mice
Source: Genes (Basel). 2023 Jul 27;14(8):1531. doi: 10.3390/genes14081531 (PMC10454722; doi:10.3390/genes14081531)
Supplement: Supplementary file 1 [file genes-14-01531-s001.zip › Supplemental materials/Table S5.pdf]

Table S5. Enriched canonical pathways with undetermined regulation (|z score| ≤ 2.0 or uncalculatable) in *Uhrf2*$^{-/-}$ LSK cells in RNA sequencing determined by IPA™.

No.	Ingenuity Canonical Pathways	-log(p-value)
1	Virus Entry via Endocytic Pathways	5.64
2	Glucocorticoid Receptor Signaling	5.10
3	Pyrimidine Ribonucleotides De Novo Biosynthesis	5.00
4	Small Cell Lung Cancer Signaling	4.79
5	STAT3 Pathway	4.71
6	Th1 and Th2 Activation Pathway	4.55
7	CLEAR Signaling Pathway	4.40
8	Hereditary Breast Cancer Signaling	4.23
9	Th2 Pathway	4.18
10	Pyrimidine Ribonucleotides Interconversion	4.13
11	Sirtuin Signaling Pathway	4.05
12	Molecular Mechanisms of Cancer	3.45
13	Role of BRCA1 in DNA Damage Response	3.41
14	Pancreatic Adenocarcinoma Signaling	3.39
15	PI3K/AKT Signaling	3.37
16	NAD Signaling Pathway	3.34
17	UVA-Induced MAPK Signaling	3.32
18	Ovarian Cancer Signaling	3.29
19	Assembly of RNA Polymerase II Complex	3.25
20	Non-Small Cell Lung Cancer Signaling	3.20
21	Telomerase Signaling	3.05
22	HGF Signaling	3.04
23	B Cell Activating Factor Signaling	2.92
24	Th1 Pathway	2.92
25	IL-10 Signaling	2.87
26	Prostate Cancer Signaling	2.69
27	Purine Ribonucleosides Degradation to Ribose-1-phosphate	2.68
28	Glioma Invasiveness Signaling	2.67
29	Production of Nitric Oxide and Reactive Oxygen Species in Macrophages	2.64
30	Salvage Pathways of Pyrimidine Ribonucleotides	2.63
31	Estrogen-mediated S-phase Entry	2.62

32	NAD biosynthesis II (from tryptophan)	2.61
33	Cholesterol Biosynthesis I	2.61
34	Cholesterol Biosynthesis II (via 24,25-dihydrolanosterol)	2.61
35	Cholesterol Biosynthesis III (via Desmosterol)	2.61
36	Role of JAK1 and JAK3 in γc Cytokine Signaling	2.55
37	April Mediated Signaling	2.51
38	Necroptosis Signaling Pathway	2.48
39	Macropinocytosis Signaling	2.47
40	Xenobiotic Metabolism Signaling	2.43
41	IL-17A Signaling in Fibroblasts	2.39
42	MIF Regulation of Innate Immunity	2.33
43	CD40 Signaling	2.29
44	Role of Tissue Factor in Cancer	2.27
45	NAD Biosynthesis from 2-amino-3-carboxymuconate Semialdehyde	2.27
46	Adenine and Adenosine Salvage III	2.27
47	ID1 Signaling Pathway	2.26
48	IL-6 Signaling	2.25
49	Heme Degradation	2.20
50	Role of IL-17A in Arthritis	2.18
51	RAC Signaling	2.18
52	Caveolar-mediated Endocytosis Signaling	2.16
53	GDNF Family Ligand-Receptor Interactions	2.10
54	NGF Signaling	2.08
55	Antioxidant Action of Vitamin C	2.06
56	Role Of Chondrocytes In Rheumatoid Arthritis Signaling Pathway	2.02
57	Neutrophil Extracellular Trap Signaling Pathway	2.00
58	Bladder Cancer Signaling	1.97
59	Protein Ubiquitination Pathway	1.94
60	Renal Cell Carcinoma Signaling	1.93
61	p53 Signaling	1.92
62	Ribonucleotide Reductase Signaling Pathway	1.92
63	Iron homeostasis signaling pathway	1.87
64	Melanoma Signaling	1.86
65	Lipoate Biosynthesis and Incorporation II	1.84
66	Xanthine and Xanthosine Salvage	1.84

67	Adipogenesis pathway	1.84
68	MSP-RON Signaling In Macrophages Pathway	1.84
69	Vitamin-C Transport	1.81
70	PPAR Signaling	1.80
71	Folate Transformations I	1.80
72	Paxillin Signaling	1.80
73	UVC-Induced MAPK Signaling	1.79
74	Prolactin Signaling	1.76
75	Angiopoietin Signaling	1.76
76	Superpathway of Cholesterol Biosynthesis	1.74
77	Cancer Drug Resistance By Drug Efflux	1.73
78	Unfolded protein response	1.69
79	LPS/IL-1 Mediated Inhibition of RXR Function	1.69
80	Regulation of Cellular Mechanics by Calpain Protease	1.69
81	Pyridoxal 5'-phosphate Salvage Pathway	1.68
82	3-phosphoinositide Degradation	1.68
83	3-phosphoinositide Biosynthesis	1.68
84	Natural Killer Cell Signaling	1.68
85	TR/RXR Activation	1.67
86	Acute Phase Response Signaling	1.66
87	PAK Signaling	1.66
88	Neuregulin Signaling	1.66
89	Role of p14/p19ARF in Tumor Suppression	1.65
90	Apoptosis Signaling	1.65
91	Ceramide Signaling	1.65
92	Mouse Embryonic Stem Cell Pluripotency	1.65
93	BMP signaling pathway	1.65
94	Phagosome Maturation	1.64
95	Prostanoid Biosynthesis	1.62
96	Glycine Betaine Degradation	1.62
97	Fc Epsilon RI Signaling	1.62
98	Role of JAK family kinases in IL-6-type Cytokine Signaling	1.61
99	IL-17 Signaling	1.60
100	DNA Methylation and Transcriptional Repression Signaling	1.59
101	Selenocysteine Biosynthesis II (Archaea and Eukaryotes)	1.58

102	Choline Biosynthesis III	1.57
103	Role of Macrophages, Fibroblasts and Endothelial Cells in Rheumatoid Arthritis	1.55
104	Lymphotoxin β Receptor Signaling	1.55
105	Xenobiotic Metabolism AHR Signaling Pathway	1.54
106	Cell Cycle: G1/S Checkpoint Regulation	1.52
107	IL-17A Signaling in Gastric Cells	1.52
108	Role of MAPK Signaling in Promoting the Pathogenesis of Influenza	1.51
109	IL-2 Signaling	1.50
110	TNFR2 Signaling	1.49
111	Germ Cell-Sertoli Cell Junction Signaling	1.48
112	Cell Cycle Regulation by BTG Family Proteins	1.48
113	Adenosine Nucleotides Degradation II	1.45
114	CD27 Signaling in Lymphocytes	1.44
115	CNTF Signaling	1.44
116	PTEN Signaling	1.42
117	Stearate Biosynthesis I (Animals)	1.42
118	Sumoylation Pathway	1.42
119	GM-CSF Signaling	1.42
120	D-myo-inositol (1,4,5,6)-Tetrakisphosphate Biosynthesis	1.42
121	D-myo-inositol (3,4,5,6)-tetrakisphosphate Biosynthesis	1.42
122	Antigen Presentation Pathway	1.41
123	ERK/MAPK Signaling	1.41
124	Clathrin-mediated Endocytosis Signaling	1.40
125	Methylglyoxal Degradation I	1.40
126	Guanine and Guanosine Salvage I	1.40
127	Uridine-5'-phosphate Biosynthesis	1.40
128	Hypusine Biosynthesis	1.40
129	Geranylgeranyldiphosphate Biosynthesis	1.40
130	Thiosulfate Disproportionation III (Rhodanese)	1.40
131	Adenine and Adenosine Salvage I	1.40
132	Tyrosine Biosynthesis IV	1.40
133	L-serine Degradation	1.40
134	Role of CHK Proteins in Cell Cycle Checkpoint Control	1.39
135	Phosphatidylcholine Biosynthesis I	1.38

136	Glycoaminoglycan-protein Linkage Region Biosynthesis	1.38
137	Autophagy	1.38
138	D-myo-inositol-5-phosphate Metabolism	1.37
139	Ferroptosis Signaling Pathway	1.36
140	NF-κB Activation by Viruses	1.36
141	Activation of IRF by Cytosolic Pattern Recognition Receptors	1.36
142	Assembly of RNA Polymerase I Complex	1.33
143	Insulin Receptor Signaling	1.33
